# Comparative Genomic Analysis of *TCP* Genes in Six Rosaceae Species and Expression Pattern Analysis in *Pyrus bretschneideri*

**DOI:** 10.3389/fgene.2021.669959

**Published:** 2021-05-17

**Authors:** Yu Zhao, Xueqiang Su, Xinya Wang, Mengna Wang, Xujing Chi, Muhammad Aamir Manzoor, Guohui Li, Yongping Cai

**Affiliations:** ^1^School of Life Sciences, Anhui Agricultural University, Hefei, China; ^2^Institute of Sericulture, Anhui Academy of Agricultural Sciences, Hefei, China

**Keywords:** *TCP* genes, flowers, development of fruit, expression patterns, genome-wide

## Abstract

*TCP* is a plant-specific transcription factor that plays an important role in flowering, leaf development and other physiological processes. In this study, we identified a total of 155 *TCP* genes: 34 in *Pyrus bretschneideri*, 19 in *Fragaria vesca*, 52 in *Malus domestica*, 19 in *Prunus mume*, 17 in *Rubus occidentalis* and 14 in *Prunus avium*. The evolutionary relationship of the *TCP* gene family was examined by constructing a phylogenetic tree, tracking gene duplication events, performing a sliding window analysis. The expression profile analysis and qRT-PCR results of different tissues showed that *PbTCP10* were highly expressed in the flowers. These results indicated that *PbTCP10* might participated in flowering induction in pear. Expression pattern analysis of different developmental stages showed that *PbTCP14* and *PbTCP15* were similar to the accumulation pattern of fruit lignin and the stone cell content. These two genes might participate in the thickening of the secondary wall during the formation of stone cells in pear. Subcellular localization showed that *PbTCPs* worked in the nucleus. This study explored the evolution of *TCP* genes in six Rosaceae species, and the expression pattern of *TCP* genes in different tissues of “Dangshan Su” pear. Candidate genes related to flower induction and stone cell formation were identified. In summary, our research provided an important theoretical basis for improving pear fruit quality and increasing fruit yield by molecular breeding.

## Introduction

*TCP* (TEOSINTE BRANCHED I, CYCLOIDEA, PROLIFERATING CELL FACTOR I) transcription factors are unique to plants and play an important role in all aspects of plant growth and development (Uberti-Manassero et al., 2016; Lucero et al., 2017). The amino acid sequences encoded by members of the *TCP* family generally have a basic helix loop helix structure. The second helical region has a specific LXXLL motif, which can interact with DNA or protein. Based on their structures, the *TCP* family can be divided into two subfamilies. Class I, the TCP-P subfamily, is also called PCF subfamily. Class II, the TCP-C subfamily, includes CYC/TB1, and CIN. The most significant difference between the two subfamilies is that PCF subfamily lacks four amino acids in the basic region, and the members of CYC/TB1 subfamily specifically contain a hydrophilic α helix (R domain) rich in polar amino acids which does not exist in other members (Cubas et al., 1999).

The *TCP* gene was first identified in maize (*Zea mays*) (teosinte branched 1, *TB1*), snapdragon (*Antirrhinum majus*) (cycloidea, *CYC*) and rice (*Oryza sativa*) (proliferating cell factors 1 and 2, *PCF1/PCF2*) (Luo et al., 1996; Doebley et al., 1997; Kosugi and Ohashi, 1997; Cubas et al., 1999). Class I transcription factors can promote cell differentiation and plant growth (Aguilar-Martinez and Sinha, 2013). For example, *TCP14* and *TCP15* can regulate *Arabidopsis* seed germination by activating the gibberellin-dependent cell cycle (Resentini et al., 2015). At the same time, it has been reported that *TCP14* and *TCP15* regulate cell proliferation in leaves and flowers, thus affecting the length between nodes and leaf traits (Kieffer et al., 2011). Overexpression of *TCP16* can regulate the process of plant differentiation, resulting in the formation of ectopic meristems (Uberti-Manassero et al., 2016). Class II, compared with Class I, mainly inhibit cell differentiation and plant growth (Manassero et al., 2013; Huang and Irish, 2015). The *CYC* gene affects the symmetry of flowers in many plants, such as *Antirrhinum majus* (Luo et al., 1996, 1999), *Lotus corniculatus* (Feng et al., 2006), and *Gerbera happipot* (Broholm et al., 2008). It inhibits the formation of floral organs by inhibiting the differentiation of cells, and ultimately affects floral symmetry. The transcription factors of the CIN subfamily can regulate the development of plant leaves. Compared with the wild type, the leaf area of snapdragon mutant (*cin*) and *Arabidopsis* mutant (*cin*) increased, and the leaves were curled and wrinkled (Nath et al., 2003; Palatnik et al., 2003). There were many leaflets on the compound leaves of the tomato mutant (*cin*), and the excessive growth of the leaf edge caused bending deformation (Ori et al., 2007). The *TCP* gene of maize (*TB1*) and *Arabidopsis* (*BRC1*) can inhibit the growth of axillary buds and reduce the number of branches above ground (Hubbard et al., 2002; Aguilar-Martinez and Sinha, 2013).

Flowering is an important life activity in plants and is a key step in the transformation from vegetative growth to reproductive growth. The *TCP* transcription factor plays an important role in flower induction (Zhao et al., 2018; Li et al., 2019). Previous studies found that *TCP15* can regulate flowering by binding to the promoter of SUPPRESSOR OF OVEREXPRESSION OF CONSTANS 1 (*SOC1*) (Lucero et al., 2017). In contrast to *TCP15*, *TCP20*, and *TCP22* delay flowering by CIRCADIAN CLOCK ASSOCIATED 1 (*CCA1*) (Wu et al., 2016). CIN-TCP subfamily, represented by *TCP4*, can interact with FLOWERING BHLH (*FBH*) and its co-promoter to regulate the flowering process (Liu et al., 2017). *TCP5* can regulate petal growth by ethylene (Van Es et al., 2018). In conclusion, two subfamily members of the *TCP* are involved in regulating flower growth and development (Li et al., 2019). Among the Rosaceae species, fruit trees make up for the majority. Flowering is the starting point of the reproductive stage of fruit trees. The quantity and quality of flowering is an important factor directly affecting the yield of fruit.

With a long history of cultivation, “Dangshan Su” pear (*Pyrus bretschneideri* cv. Dangshan Su) is one of the most important pear resources in China and occupies an important position in the fruit market (Su et al. 2019). The stone cell content and the size in pear are important factors affecting the quality of fruit (Zhang et al., 2017). Thickening of the secondary wall is an important step in the formation of stone cells (Cheng et al., 2019). Therefore, the thickening of the secondary wall and the deposition of lignin have a great influence on the quality of pear. In the previous study, *TCP4* can activate the promoter of *VND7* to increase the formation of the secondary wall and up-regulate genes related to lignin and cellulose synthesis (Nag et al., 2009). *TCP24* negatively regulates the secondary wall thickness of anther endothecium, resulting in anther dehiscence and pollen release and eventually male sterility (Wang et al., 2015). *GbTCP5* is involved in the formation of secondary wall (Wang et al., 2020). Up-regulation of *GhTCP4* expression in cotton can activate the synthesis of secondary walls in fibrocyte, thus obtaining fiber with thicker cell walls (Cao et al., 2020). In conclusion, we speculate that the *TCP* family members may be involved in flower induction and stone cell formation during fruit development of “Dangshan Su” pear. Systematic study of the *TCP* family is of great importance for improving pear fruit quality.

Although the identification and functions of *TCP* genes have been studied in *Arabidopsis*, snapdragon, the *TCP* genes in pear remain unstudied. In this study, 155 *TCP* genes were identified in pear (*Pyrus bretschneideri*), strawberry (*Fragaria vesca*), plum (*Prunus mume*), raspberry (*Rubus occidentalis*), cherry (*Prunus avium*), apple (*Malus domestica*). The phylogenetic relationships of *TCP* genes in six Rosaceae species were elucidated by constructing phylogenetic tree, tracking gene duplication events, performing a sliding window analysis. Candidate genes related to flowering regulation (*PbTCP10*) were identified by qRT-PCR and expression profile analysis. In addition, based on the analysis of expression patterns in pear and bioinformatics analysis results, we predicted that *PbTCP14* and *PbTCP15* were the key factors of pear fruit stone cell development. This study provided important theoretical basis and gene resources for improving pear fruit quality.

## Materials and Methods

### Identification of *TCP* Genes in Rosaceae

In this study, the *Pyrus bretschneideri* genome was downloaded from GIGADB datasets^1^. In addition, Rosaceae genomes (*Fragaria vesca*, *Rubus occidentalis*, *Prunus avium*, *Malus domestica*, *Prunus mume*) were obtained from the following website (see text footnote 1)^2^. Bioedit software was used to construct the local protein database. The conserved domain of *TCP* was used as the query sequence for Blastp search (*E* = 0.001) from the local protein database (Supplementary Table 1). The SMART online software was used to search and analyze *TCP* conserved region (Letunic et al., 2012). ExPASY online website was used to predict the molecular weight and basic information of *TCP* genes (Artimo et al., 2012). Wolf PSORT was used to the predicted subcellular localization of all *TCP* genes^3^ (Horton et al., 2007). Blast2GO sofware was used to implement Gene Ontology (GO) annotation analysis. Visualization of GO classifcations was used the WEGO online tool (Ye et al., 2006). The data of different tissues of Chinese white pear were downloaded from NCBI under the following accession numbers SRR8119889, SRR8119890, SRR8119891, SRR8119892, SRR8119893, SRR8119894, SRR8119895, SRR8119896, SRR8119897, SRR8119898, SRR8119899, SRR8119900, and SRR8119901 (Cao et al., 2019).

### Phylogenetic Construction and Conserved Structure Analysis of *TCP* Genes

All TCP proteins sequenced were analyzed by ClustalW tool in MEGA7.0 software. The phylogenetic tree was constructed by MEGA7.0 software with the Neighbor-Joining method and other default parameters (Kumar et al., 2016). The *TCP* genes of *Arabidopsis* were obtained from previous study (Yao et al., 2007). Subsequently, the TCP protein sequence was used to obtain the conserved motif region by MEME online software (Bailey et al., 2015). In the conservative region prediction, we chose the interval range of 6–200, and the number of conservative regions was generally not less than 20.

### Chromosomal Localization and Gene Duplication Events

The chromosome information of six Rosaceae species was obtained from the public genomic database, and MapInspect software was used to display the members of *TCP* gene family on their respective chromosomes (Ma et al., 2015; Zhu et al., 2015). The determination of gene duplication events mainly depended on the following principles: (1) Two genes were located in the same branch of the evolutionary tree, and the similarity of amino acid sequence was more than 80% (2) Two genes were located on the same chromosome and the distance between them was at least 200 kb, we considered these two genes tandem duplicated events (3) Two genes located on different chromosomes were defined as fragment duplication events (4) The non-synonymous substitution (Ka) and synonymous substitution (Ks) values of a replicated gene pair were calculated by DnaSP v5.0 software (Ka/Ks > 1 was positive selection, Ka/Ks < 1 was purification selection, Ka/Ks = 1 was neutral selection). Finally, DnaSP v5.0 software was used to analyze the gene duplication events by sliding window to determine the selection modest each amino acid site (Librado and Rozas, 2009). The specific parameters were as follows: the window size was 150 bp, and each step moved 9 bp.

### Chinese White Pear *TCP* Gene Promoter *cis-*Acting Element Analysis

We obtained the promoter sequence of *TCP* genes from the pear genome database. In the database, we found promoter about 1,500–2,000 bp upstream of the initiation codon (ATG) of each *TCP* genes. The online Plantcare database was used to analyze *cis*-acting elements^4^ (Rombauts et al., 1999).

### RNA Extraction and qRT-PCR Analysis

The plant material was collected from the “Dangshan Su” pear, which grown in the Dangshan County (Anhui Province, China). The fruits samples were taken on 15, 39, 47, 55, 63, 79, and 102 DAP (days after pollination), as well as the other tissue samples such as flowers, stems, and leaves were also collected on the same year. The 102 DAP fruit was used for expression analysis in different tissues. The buds of “Dangshan Su” pear were treated with gibberellin (GA) (700 mg⋅L^–1^). Then, the samples of 0, 2, 4, 6, 8, and 12 HPT (h post-treatment) were collected and stored at −80°C. Finally, the RNA was extracted using a plant RNA extraction kit from Tiangen (Beijing, China). Reverse transcribed by PrimeScript^TM^ RT reagent kit (Takara, Kusatsu, Japan) and each reaction consisted of 1 μg of RNA. The qRT-PCR primers of *TCP* genes were designed by Beacon Designer 7 (Supplementary Table 2). The qRT-PCR system consisted of 10 μL SYBR Premix Ex TaqTM II, 2 μL cDNA, 6.4 μL water, and 0.8 μL forward primer and reverse primer. The pear *Tubulin* gene (AB239680.1) used as an internal reference (Su et al. 2019). Introduction manual used for the procedure and repeat 3 times for each sample. The relative expression levels were calculated using the 2^–ΔΔ*CT*^ method (Livak and Schmittgen, 2001).

### Subcellular Localization of *PbTCP6, 13*, and *17*

Full length sequence specific primers and primers with restriction sites were designed using Primer Premier 5.0 software based on the full-length sequences of *PbTCP6*, *13*, and *17* (*PbTCP6*, *PbTCP13*, and *PbTCP17* both used *Ncob* I and *Spe*I restriction endonuclease sites) (Supplementary Table 3). “Dangshan Su” pear fruit cDNA as template was used. Finally, each gene fragment was ligated into the pCAMBIA1304 (GenBank: AF234300.1) vector used T_4_ DNA ligase (Takara, China) at 16°C for 3 h to obtain complete pCAMBIA1304-*PbTCP6*, *13*, and *17* recombinant plasmids.

The pCAMBIA1304-*PbTCP6*, *13*, and *17* recombinant plasmid, and pCAMBIA1304 empty plasmid *Agrobacterium tumefaciens* were cultured. Then mixed the infection liquid (10 mM MES, 10 mM MgCl2, 0.1 mM AS). Finally, the OD_600_ value of bacteria solution was adjusted between 0.6 and 0.8. The growing well and flat tobacco leaves were selected for injection. The infection solution was injected into the lower epidermis of tobacco leaf and cultured in the dark for 48 h (Sufficient water should be kept during dark culture). After dark culture, the tobacco leaf tissue near the injection hole was selected and placed on the glass slide. The fluorescence of GFP protein was observed under confocal laser scanning microscopy.

## Results

### Identification, Characterization, and Phylogenetic Analysis of *TCP* Genes

**FIGURE 1:**
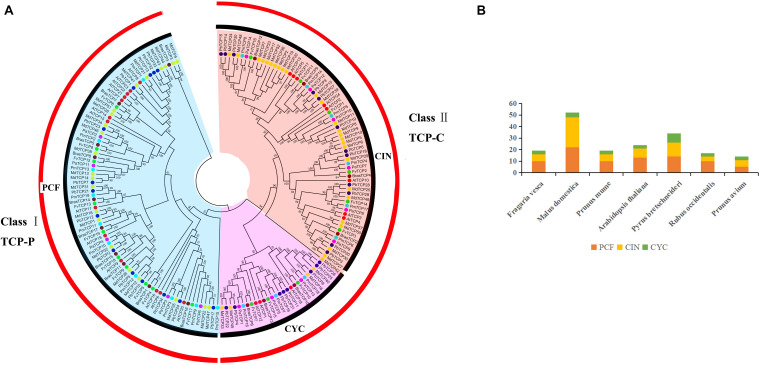
Phylogenetic relationships and subfamily designations in TCP proteins from *Pyrus bretschneideri*, *Fragaria vesca*, *Prunus mume*, *Rubus occidentalis*, *Prunus avium*, *Malus domestica*, and *Arabidopsis thaliana*. **(A)** Interspecific phylogenetic tree of TCP protein sequences from 7 species. **(B)** Number of genes in each subfamily of 7 species.

Firstly, we used the HMM for obtaining PF03634 of the conservative domain as the search criteria to compare six Rosaceae species in the protein database (Supplementary Table 1). Thirty-four *TCP* genes were identified in pear and 121 genes were identified in the other five Rosaceae species, including strawberry (19), apple (52), plum (19), raspberry (17), and cherry (14) (Table 1). Finally, we constructed phylogenetic trees with six Rosaceae species and *Arabidopsis* using the Neighbor-Joining method. The phylogenetic tree was divided into two subgroups: PCF was in Class I, CIN, and CYC were in Class II (Figure 1). Among them, CYC members had the least members, 3 in strawberry, 4 in apple, 3 in plum, 3 in *Arabidopsis*, 8 in pear, 3 in raspberry, 3 in cherry. Compared to CYC, PCF had more members, apple had 22 members, followed by pear (14), *Arabidopsis* (13), strawberry (10), raspberry (10), plum (10) and cherry (5) (Table 1). Additionally, we calculated the physicochemical parameters of *TCP* genes in six Rosaceae species. Among these six Rosaceae species, the pI value was 4.62–10.65 and the molecular weight ranges from 12.82 to 69.01. The GRAVY values of all TCP proteins were negative. 99% of *TCP* genes were located in the nucleus (Table 2 and Supplementary Table 4).

**TABLE 1:** Number of genes in each subfamily of 7 species.

	PCF	CIN	CYC	Total
*Fragaria vesca*	10	6	3	19
*Malus domestica*	22	26	4	52
*Prunus mume*	10	6	3	19
*Arabidopsis thaliana*	13	8	3	24
*Pyrus bretschneideri*	14	12	8	34
*Rubus occidentalis*	10	4	3	17
*Prunus avium*	5	6	3	14
				

**TABLE 2:** Basic information of *TCP* genes in *Pyrus bretschneideri*.

Gene name	Gene ID	Chromosome	AA	KD	pI	GRAVY	Preditced subcellular localization
PbTCP1	Pbr018420.1	Chr1	250	26.93	9.76	–0.410	nucl
PbTCP2	Pbr018814.1	Chr2	307	33.03	5.64	–0.473	nucl
PbTCP3	Pbr025856.1	Chr3	440	47.80	6.53	–0.767	nucl
PbTCP4	Pbr013244.1	Chr3	461	51.33	9.12	–0.866	nucl
PbTCP5	Pbr021770.1	Chr4	380	39.69	5.68	–0.474	nucl
PbTCP6	Pbr000450.1	Chr5	217	24.57	6.75	–0.841	nucl
PbTCP7	Pbr011454.1	Chr6	411	43.73	7.13	–0.668	nucl
PbTCP8	Pbr020246.1	Chr6	462	52.90	6.97	–1.014	nucl
PbTCP9	Pbr020171.1	Chr6	377	42.02	8.00	–0.761	nucl
PbTCP10	Pbr001559.1	Chr6	377	39.36	5.63	–0.411	nucl
PbTCP11	Pbr013717.1	Chr6	376	42.17	8.99	–0.707	nucl
PbTCP12	Pbr013906.1	Chr7	366	37.88	6.25	–0.328	nucl
PbTCP13	Pbr026562.3	Chr8	601	62.82	7.58	–0.705	nucl
PbTCP14	Pbr006457.1	Chr9	383	42.07	7.32	–0.791	nucl
PbTCP15	Pbr006477.1	Chr9	383	42.10	7.97	–0.790	nucl
PbTCP16	Pbr030633.1	Chr9	390	43.73	9.17	–0.758	nucl
PbTCP17	Pbr041545.1	Chr9	323	34.51	9.02	–0.802	nucl
PbTCP18	Pbr016172.1	Chr10	217	24.71	8.71	–0.909	nucl
PbTCP19	Pbr039609.1	Chr10	483	52.57	8.36	–0.828	nucl
PbTCP20	Pbr038238.1	Chr11	430	46.70	6.44	–0.664	nucl
PbTCP21	Pbr020546.1	Chr12	220	24.01	6.60	–0.441	nucl
PbTCP22	Pbr027488.1	Chr13	477	52.91	9.06	–0.823	nucl
PbTCP23	Pbr039105.1	Chr13	401	42.85	6.73	–0.624	nucl
PbTCP24	Pbr035636.1	Chr13	249	26.82	9.67	–0.575	nucl
PbTCP25	Pbr007075.1	Chr14	497	56.49	7.44	–0.978	nucl
PbTCP26	Pbr007125.1	Chr14	471	53.46	6.47	–0.955	nucl
PbTCP27	Pbr007197.1	Chr14	373	41.40	7.30	–0.739	nucl
PbTCP28	Pbr031206.1	Chr15	345	37.83	6.40	–0.725	nucl
PbTCP29	Pbr022498.1	Chr17	351	38.30	5.98	–0.752	nucl
PbTCP30	Pbr006641.1	Chr17	380	41.88	7.03	–0.832	nucl
PbTCP31	Pbr003924.1	scaffold1180.0	307	34.67	9.22	–0.747	Chlo
PbTCP32	Pbr039926.1	scaffold868.0	603	63.39	8.74	–0.722	Nucl
PbTCP33	Pbr039901.1	scaffold868.0	603	63.39	8.74	–0.722	Nucl
PbTCP34	Pbr037196.1	scaffold751.0	247	27.76	10.42	–0.592	Nucl

*The TCP genes of Pyrus bretschneideri identified in this study are listed.*

To further understanding about the potential function of *TCP* family in six Rosaceae species, we analyzed 155 *TCP* genes by GO analysis. The results showed that *TCP* genes could be divided into three categories: cellular component, biological process and molecular function. In molecular function, most genes were enriched in transcriptional regulatory activity. In biological process, *TCP* genes of six Rosaceae species were found in three GO terms (regulation of biological process, biological regulation, metabolic process). Among cellular components, organelle part and membarane enclosed activity were only found in a few genes of apple (Supplementary Figure 1 and Supplementary Table 5).

### Conserved Structure Analysis of *TCP* Genes

**FIGURE 2:**
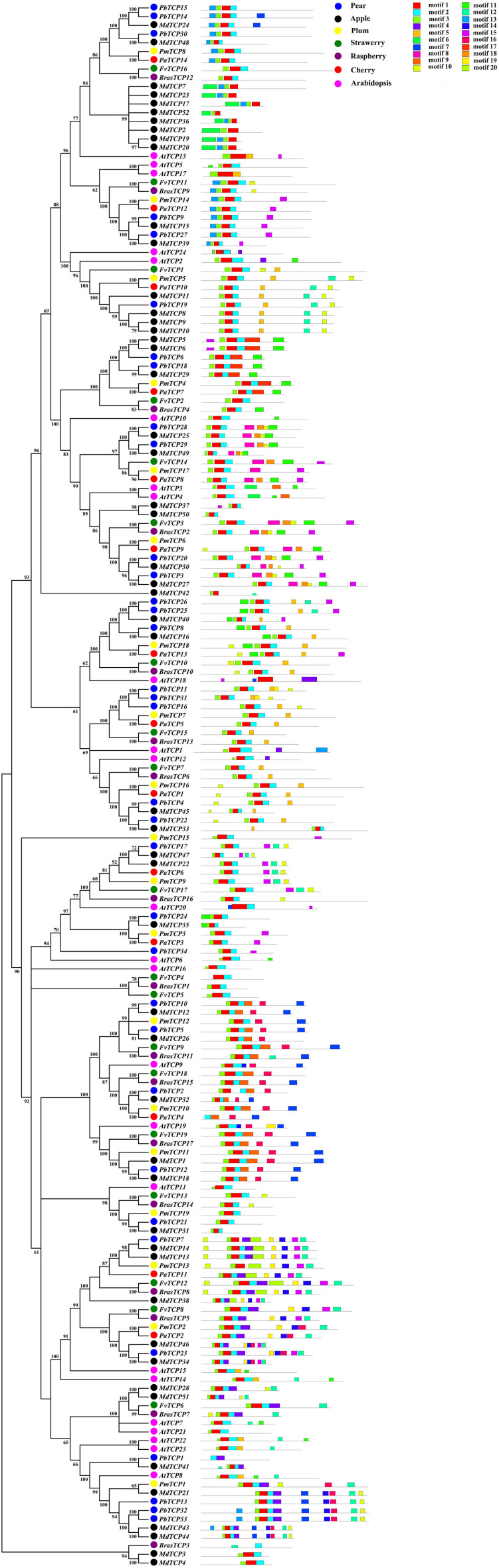
Predicted *Pyrus bretschneideri*, *Fragaria vesca*, *Prunus mume*, *Rubus occidentalis*, *Malus domestica*, *Prunus avium*, and *Arabidopsis thaliana* TCP protein conserved motifs.

In order to study the evolutionary relationship of *TCP* genes, we analyzed the conservative structure in six Rosaceae species. In this study, we used MEME to predict 20 motifs of *TCP* genes, and the results showed that members of *TCP* genes were highly conservative (motif 1, 2, 3) (Figure 2). We used ClustalX 2.0 to align the protein sequences. After alignment, TCP proteins were divided into two subgroups. Most of the *TCP* domains in each species were composed of 55–60 amino acids, which conform to the basic HLH structure (Supplementary Figures 2–7). In the basic region of *TCP*, several specific amino acids could bind to DNA, which was relatively conservative. In the region of helix 1 and 2, the amino acid sequences of TCP-P and TCP-C were different. In the TCP-C subfamily, most of the *CYC* genes contained an R domain (Supplementary Figures 2–7).

### Chromosomal Location and Duplication Events of *TCP* Genes in Six Rosaceae Species

**FIGURE 3:**
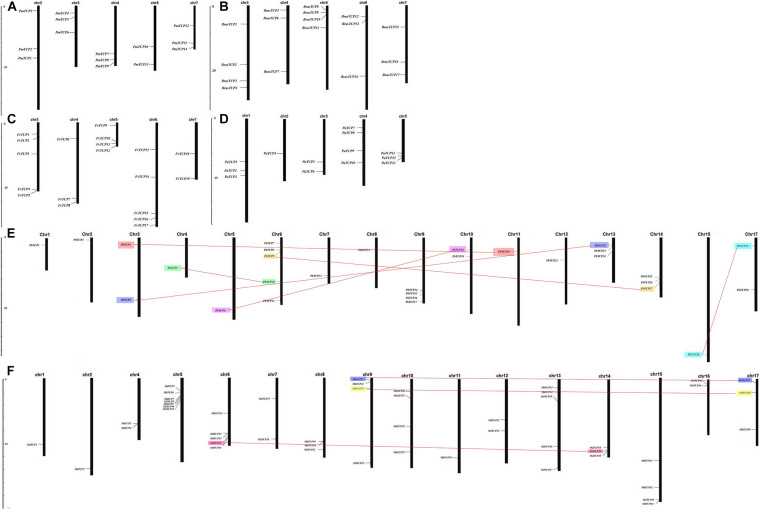
Chromosomal locations of Six Rosaceae species. Chromosomal locations of *TCP* genes in **(A)** *Prunus mume*, **(B)** *Rubus occidentalis*, **(C)** *Fragaria vesca*, **(D)** *Prunus avium*, **(E)** *Pyrus bretschneideri*, and **(F)** *Malus domestica.* Duplicated gene pairs are connected with colored lines.

According to the whole genome data of strawberry, apple, plum, pear, raspberry, cherry, the exact chromosome physical location information of all *TCP* genes were determined (Figure 3). In the pear, 4 out of 34 *TCP* genes were not located on any chromosome, and 30 *TCP* genes were located on 16 chromosomes (except chromosome 16). In strawberry, *TCP* genes were located on chromosome 3, 4, 5, 6, and 7. Five genes in plum were not located on any chromosome, and other genes were located on chromosome 2, 3, 4, 5, and 7. In raspberry, *TCP* genes were mainly distributed on chromosomes 3 and 5, and other genes were distributed on chromosomes 4, 6, and 7. In cherry, four genes were distributed on chromosome 4, three genes on chromosome 1 and 5, and the remaining three genes on chromosome 2 and 3. In apple, there are no genes on chromosome 3 and three genes are not located on any chromosome.

Among the six Rosaceae species, only 11 gene duplication events were identified in pear and apple (Supplementary Table 6). Seven duplication events were identified in pear and 4 in apple. In order to study the effect of duplication events on gene evolution, we counted the values of Ka, Ks, and Ka/Ks of 11 duplicated gene pairs were analyzed them. Among these 11 gene duplication events, Ka/Ks values were <1, with the maximum value of 0.907 (*MdTCP24-MdTCP48*) and the minimum value of 0.097 (*MdTCP28-MdTCP51*). These results indicated that *TCP* family genes were mainly affected by purifying selection during evolution.

**FIGURE 4:**
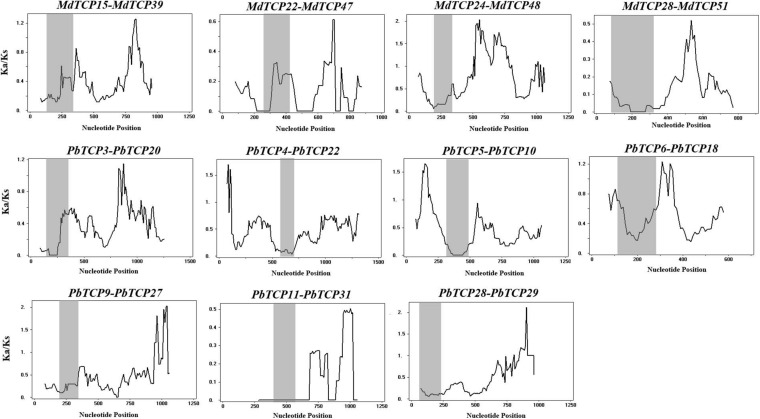
Sliding window plots of duplicated *TCP* genes in *Pyrus bretschneideri* and *Malus domestica.* The gray shaded portion indicates conserved *TCP* domain. The *X*-axis indicates the synonymous distance within each gene.

Among the 11 gene duplication events, 9 pairs were fragment duplication events and two pairs were not located any chromosome. These results indicated that the expansion of *TCP* genes were mainly driven by fragment duplication. To understand the selection pressure of *TCP* family in the evolution process, we performed sliding window analysis (Figure 4). Sliding window analysis, results implied that the Ka/Ks values of *TCP* conservative domains were <1. Most coding site Ka/Ks ratios were <1, with exceptions for one or several distinct peaks (Ka/Ks > 1).

### Analysis of *cis*-Acting Elements in *TCP* Gene Promoter

**FIGURE 5:**
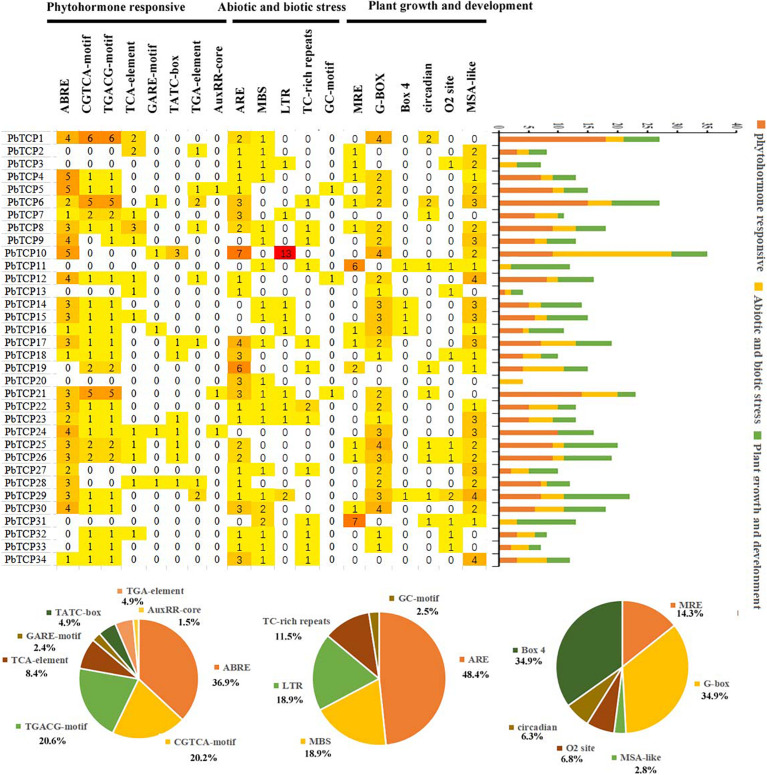
Putative *cis*-acting regulatory elements in the *PbTCP* promoters.

In plant growth and development, gene-specific expression was mainly related to *cis-*acting elements of upstream promoter. In this experiment, we had been analyzed the *cis-*acting elements of 34 members of *TCP* gene promoter in pear. We divided the functional elements into three types: plant growth and development, biological and abiotic stress responses, and phytohormone responses (Figure 5 and Supplementary Table 7). In phytohormone responses, there were many *cis*-acting elements related to the responses to hormones, including responses to methyl jasmonate (CGTCA-motif, TGACG-motif), gibberellin (TATC-box, GARE-motif), auxin (TGA-element, AuxRR-core), abscisic acid (ABRE), and salicylic acid (TCA-element). In 34 members of *TCP* family, the *cis-*acting element related to the responses to abscisic acid appeared 75 times. In plant growth and development, including the light response elements (MRE, Box 4, G-Box), cell cycle regulation (MSA-like), zein metabolism regulation (O_2_-site), day and night control (circadian), in which the proportion of light response elements was more, Box4, G-box each appeared 61 times. In biological and abiotic stress responses mainly included drought (MBS), defense and stress (TC rich repeats), hypoxia specific inducible enhancer like elements (GC motif), anaerobic (ARE), and low temperature (LTR).

### Expression Profile Analysis of *PbTCPs* in Different Tissues of Chinese White Pear

**FIGURE 6:**
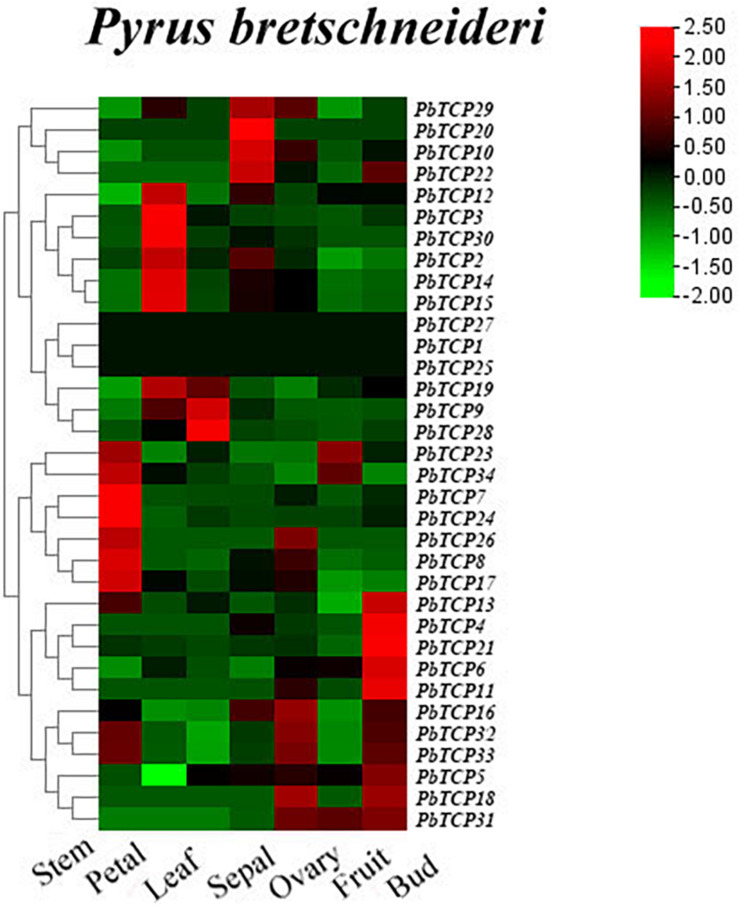
Expression profiles of *PbTCP* genes in different tissues of *Pyrus bretschneideri.* The heatmap was generated by TBtools software according to the RNA-seq database. Log2-based-fold changes were used to create a heatmap. As shown in the bar at the upper right corner, the gene transcription level is expressed in different colors on the map.

In order to further study the function of *PbTCP*s in flower, we analyzed the expression patterns of 34 *TCP* genes in petal, sepal, ovary, bud, stem, leaf according to the RNA-seq database. As shown in Figure 6, three genes (*PbTCP1*, *25*, *27*) were not expressed in all tissues. *PbTCP2*, *3*, *12*, *14*, *15*, and *30* were highly expressed in petals. The expression levels of *PbTCP16*, *18*, *31*, *32*, and *33* were higher in ovary, which might affect the growth and development of fruits in the later stage. Comparing with other tissues, the expression of almost all genes in mature fruit was relatively low. Four genes (*PbTCP10*, *20*, *22*, *29*) were highly expressed in sepal. About 30% genes were highly expressed in buds and stems.

### Expression Characteristics of Chinese White Pear *TCP* Genes

**FIGURE 7:**
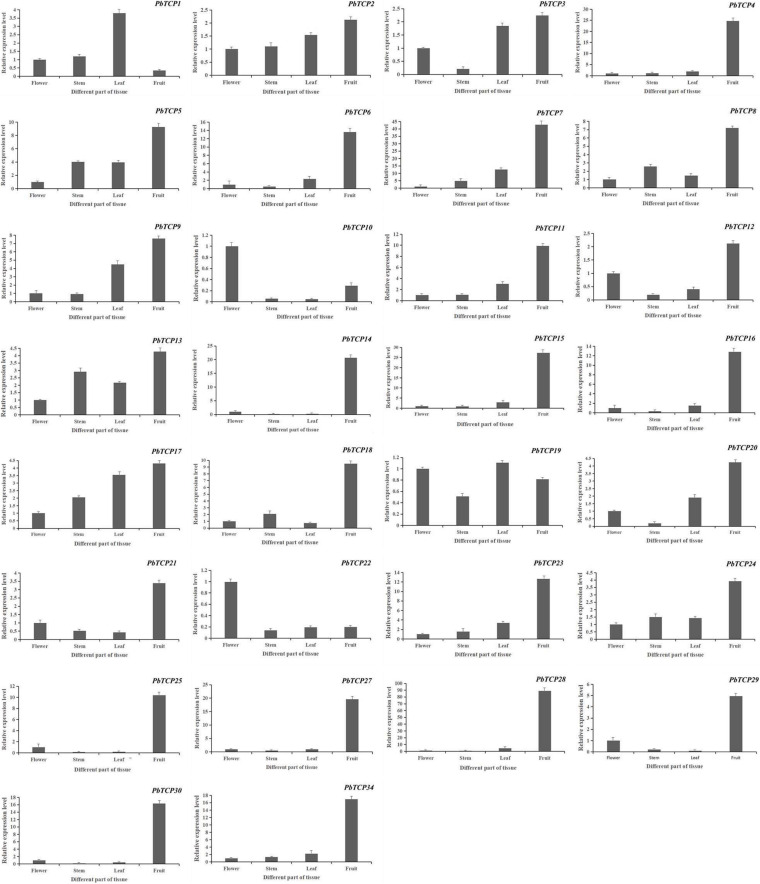
Expression pattern of *PbTCPs* across different tissues (fruit, stems, flowers, and leaves). Error bars show the standard error between three replicates.

In order to further study the function of *TCP* genes in pear, we studied the expression of *TCP* genes in different tissues. As shown in Figure 7, *PbTCP26*, *31*, *32*, and *33* were not expressed in any tissues. *PbTCP10*, *19*, and *22* were highly expressed in flowers, and *PbTCP1* was highly expressed in leaves. Other genes were highly expressed in fruits.

**FIGURE 8:**
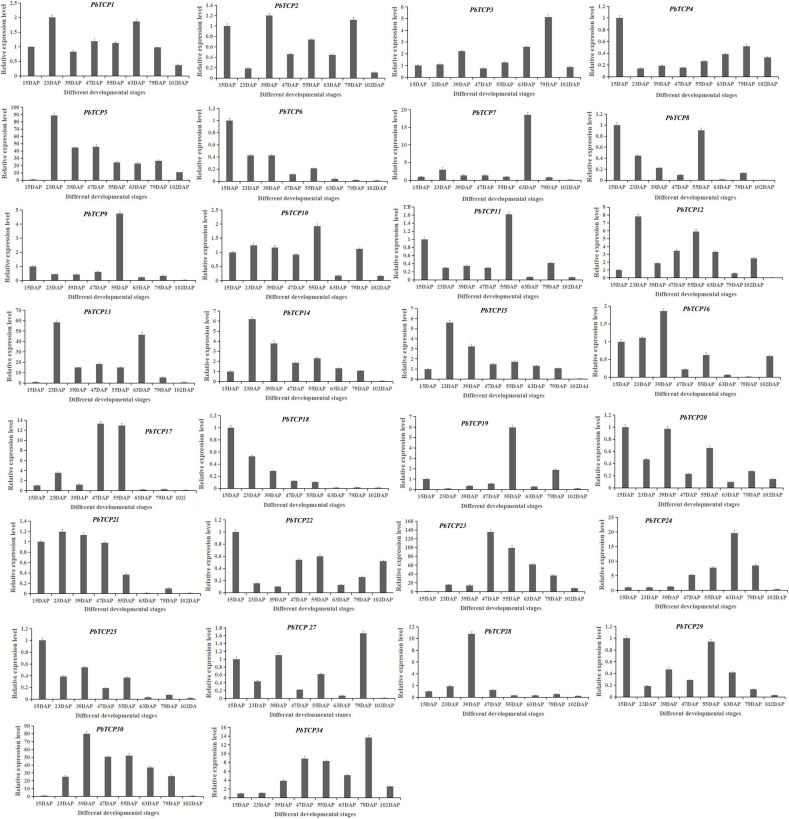
Expression pattern of *PbTCPs* across different developmental stages (15DAP, 23DAP, 39DAP, 47DAP, 55DAP, 63DAP, 79DAP, and 102DAP). Error bars show the standard error between three replicates.

We analyzed the expression level in seven development stages of Chinese white pear (Figure 8). These results showed that the expression pattern of *PbTCP7* reached a peak at 63 DAP. The expression of *PbTCP9* and *PbTCP19* reached a peak only at 55 DAP, but the expression level was very low at other developmental stages. Firstly, the expression level of *PbTCP14*, *15*, *23*, and *24* were increased, then the expression level decreased during fruit development. The expression of *PbTCP6* and *PbTCP18* reached a peak at the 15 DAP.

### Gibberellin Response Pattern Analysis of *PbTCPs*

The results of expression profile analysis and qRT-PCR showed that *PbTCP10*, *19*, and *22* were highly expressed in flowers (Supplementary Figure 8). In this experiment, the buds of “Dangshan Su” pear were treated with exogenous GA, and the expression patterns of *PbTCP10*, *19*, and *22* were analyzed. After GA treatment, these three genes expressed two response patterns. The expression of *PbTCP10* increased significantly at 2 HPT, maintained at a high level at 4–8 HPT, and returned to the initial level at 12 HPT. There was no significant change in the transcriptional level of *PbTCP19* and *PbTCP22* under exogenous GA treatment.

### Subcellular Localization of *PbTCP6, 13*, and *17*

**FIGURE 9:**
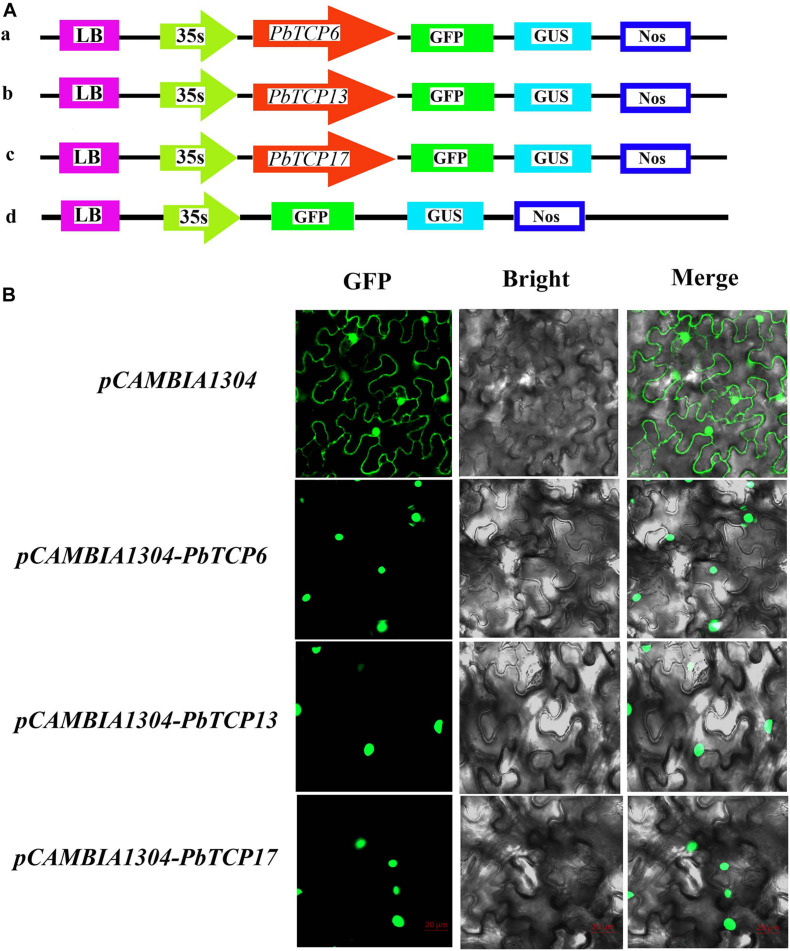
Subcellular localization of three *PbTCP* genes. **(A)** Schematic illustration of vectors pCAMBIA1304 and *PbTCPs*. **(B)** The three pCAMBIA1304-*PbTCPs* fusion proteins (pCAMBIA1304-*PbTCP6*, pCAMBIA1304-*PbTCP13*, pCAMBIA1304-*PbTCP17*), and pCAMBIA1304 as a control were transiently expressed in tobacco leaf and observed under fluorescence microscope.

The main function of transcription factors is to connect with *cis-*acting elements of gene promoter in the nucleus. In order to study the subcellular localization of *TCP* genes in pear, three *TCP* genes were connected with 35S promoter containing green fluorescent protein (GFP). These three genes and empty vector were transiently expressed in tobacco. As shown in Figure 9, these three genes were located in the nucleus, and the empty vector was located in the nucleus and cell membrane, which was consistent with the predicted results.

## Discussion

TCP proteins are transcription factors that are unique in plants and are involved in leaf development, flower symmetry, stem branching, and other biological processes. TCP proteins can also regulate the flowering process and secondary wall formation and ultimately affect plant growth and development (Nag et al., 2009; Wang et al., 2015). In this study, 155 genes were identified in six Rosaceae plants. All genes contained a *TCP* conserved domain, and their proteins were hydrophilic with a negative GRAVY value (Table 2 and Supplementary Table 4). In the six Rosaceae species, all genes were divided into two subgroups, and the number of Class I (TCP-P) members was generally greater than that of Class II (TCP-C) members. However, in apple, pear and cherry, there were more TCP-C members than TCP-P members (Table 1). According to the number of *TCP* genes in each species, there are the most *TCP* genes in apple (52), followed by pear (34). The number of *TCP* family members of apple and pear were more than other species (Table 1). These differences might be related to the evolution of the *TCP* family.

Whole-genome duplication (WGD) or polyploidy is an important driving force shaping plant evolution (Tang et al., 2008). Previous studies indicated that pear, strawberry, apple and other dicotyledons had a whole-genome duplication event before 140 million years ago (Mya). However, apple and pear experienced a whole-genome duplication event 30–40 Mya (Shulaev et al., 2011; Wu et al., 2013). After that, the chromosome number of pear and apple changed to 17, strawberry changed to 7, plum changed to 8 and raspberry changed to 7, and cherry changed to 8. These results indicated that the second WGD, the 9 chromosomes in the common ancestor of Rosaceae underwent doubling, breaking, hybridization and fusion. The conserved domains were closely related to the diversity of gene functions. The structures within a subfamily were similar, which indicated that these genes might have similar functions. In the HLH domain, the second helix region had a specific LXXLL motif, and members of the CYC/TB1 subfamily specifically contained a hydrophilic α helix (R domain) rich in polar amino acids, which did not exist in other members (Supplementary Figures 2–7). The difference in gene number and the retention of conserved structures might be due to the loss of *TCP* family genes, chromosome doubling, and selection pressure in the process of WGDs.

To understand the evolutionary patterns of *TCP* family genes in six Rosaceae species, we calculated the values of Ka and Ks (Figure 4 and Supplementary Table 6). These results showed that collateral gene pairs only existed in apple and pear, and the Ka/Ks values of all gene pairs were <1, which indicated that the *TCP* family had undergone obvious purifying selection in the evolutionary process. Interestingly, there were two gene pairs (*PbTCP28-PbTCP29*, *MdTCP24-MdTCP48*) with relatively high Ka/Ks values (>0.5), which might be due to the rapid evolution and diversification of these two genes after the duplication event.

Plant flowering is an important life activity in the process of plants transitioning from vegetative growth to reproductive growth. Many genes are involved in the flowering process of plants, such as *FLS* (Park et al., 2020), *MADS* (Tang et al., 2020), and *CDF* (Corrales et al., 2014). Recent studies have shown that *TCP* genes also play a regulatory role in plant flowering (Lucero et al., 2017; Li et al., 2019). We used public transcriptome data and qRT-PCR to obtain the expression pattern of *TCP* genes in “Dangshan Su” pear. These results showed that there was expression in all tissues results of *TCP* genes, which indicated that *TCP* genes played an important role during growth and development in pear. The qRT-PCR results in different tissues showed that *PbTCP10*, *19*, and *22* were highly expressed in flowers (Figure 7). According to expression profile analysis, *PbTCP10* and *PbTCP22* were highly expressed in the sepal. *PbTCP19* was highly expressed in the petal (Figure 6). Previous studies showed that hormones (especially GA), sugar and light also play an important role in flowering regulation (Srikanth and Schmid, 2011; Osnato et al., 2012; Cao et al., 2018). In the analysis of *cis*-acting elements, it could be seen that the promoter regions of *PbTCP10* contained GA-responsive elements (TATC-box, GARE-motif). After exogenous GA treatment, the expression patterns of *PbTCP10*, *19*, and *22* showed that the expression of *PbTCP19* and *PbTCP22* were almost not induced by GA, and the expression of *PbTCP10* increased significantly at 2 HPT (Supplementary Figure 8). These phenomenons might be due to the absence of GA response element in the promoter of *PbTCP19* and *PbTCP22*. In addition, we found that the *cis*-acting elements of *PbTCP10* promoter contained light response elements, which indicated that *PbTCP10* might be involved in photoperiodic signal (Figure 5). In conclusion, *PbTCP10* might be involved in GA regulated flowering induction pathway and regulate photoperiod.

Previous studies found that the formation of stone cells in “Dangshan Su” pear mainly occurred in the early stage of fruit formation (15–47 DAP) (Su et al. 2019). Therefore, *TCP* genes with high expression level in the early stage of fruit development might be involved in the formation of stone cells. The genes with high expression in late stage might be involved in the accumulation of sugar and the response of hormone during fruit ripening. In order to determine the effect of *TCP* genes on secondary wall formation during fruit development of “Dangshan Su” pear, qRT-PCR analysis was conducted at different stages of fruit development (Figure 8). The results showed that *PbTCP14*, *15*, *23*, and *24* increased firstly and then decreased during fruit development, which was consistent with the trend of stone cell formation, but only *PbTCP14* and *PbTCP15* were highly expressed in the early stage of fruit development. Therefore, *PbTCP14* and *PbTCP15* might be involved in the stone cell formation during fruit development of “Dangshan Su” pear.

Through comparative genomics analysis, we identified the evolution of *TCP* genes in six Rosaceae species, and screened candidate regulatory genes related to flowering (*PbTCP10*) and stone cell formation (*PbTCP14* and *PbTCP15*). In the following study, we will analysis the biological functions of these genes and provide an important theoretical basis for improving pear quality.

## Conclusion

In this work, 155 *TCP* genes were identified in six Rosaceae species. According to bioinformatics analysis, we explained the possible evolutionary patterns of *TCP* genes in six Rosaceae species. By qRT-PCR analysis of 34 *TCP* genes in different development stages and tissues of pear, we found that *PbTCP14* and *PbTCP15* might be involved in the formation of secondary wall during pear fruit development, and *PbTCP*10 might be involved in the process of flowering induction by GA. In general, these results provided a theoretical basis for improving the quality of pear.

## Data Availability Statement

The datasets presented in this study can be found in online repositories. The names of the repository/repositories and accession number(s) can be found in the article/Supplementary Material.

## Author Contributions

YZ and XS performed the experiments and wrote the manuscript. XW, MW, and XC analyzed the data. MA and GL helped to polish the language. YC conceived and designed the experiments. All authors read and approved the final manuscript.

## Conflict of Interest

The authors declare that the research was conducted in the absence of any commercial or financial relationships that could be construed as a potential conflict of interest. The handling editor declared a past co-authorship with one of the authors YC.

## Footnotes

1 http://gigadb.org/dataset/10008

2 https://www.rosaceae.org/

3 http://www.genscript.com/wolf-psort.html

4 http://bioinformatics.psb.ugent.be/webtools/plantcarere/html/

## Supplementary Material

Supplementary Figure 1 Gene Ontology (GO) analysis of TCP genes in six Rosaceae species (*Prunus mume*, *Rubus occidentalis*, *Fragaria vesca*, *Prunus avium*, *Malus domestica*, *Pyrus bretschneideri*).

Supplementary Figure 2 Multiple sequence alignment of pear *TCP* transcription factors. Alignment of the *TCP* domain for the predicted pear TCP proteins. The basic, helix I, loop, and helix II regions are indicated. Alignment of the R domain of Class II subfamily members. The sequences were aligned with ClustalW.

Supplementary Figure 3 Multiple sequence alignment of apple *TCP* transcription factors. Alignment of the *TCP* domain for the predicted apple TCP proteins. The basic, helix I, loop, and helix II regions are indicated. Alignment of the R domain of Class II subfamily members. The sequences were aligned with ClustalW.

Supplementary Figure 4 Multiple sequence alignment of raspberry *TCP* transcription factors. Alignment of the *TCP* domain for the predicted raspberry TCP proteins. The basic, helix I, loop, and helix II regions are indicated. Alignment of the R domain of Class II subfamily members. The sequences were aligned with ClustalW.

Supplementary Figure 5 Multiple sequence alignment of strawberry *TCP* transcription factors. Alignment of the *TCP* domain for the predicted strawberry TCP proteins. The basic, helix I, loop, and helix II regions are indicated. Alignment of the R domain of Class II subfamily members. The sequences were aligned with ClustalW.

Supplementary Figure 6 Multiple sequence alignment of cherry *TCP* transcription factors. Alignment of the *TCP* domain for the predicted cherry TCP proteins. The basic, helix I, loop, and helix II regions are indicated. Alignment of the R domain of Class II subfamily members. The sequences were aligned with ClustalW.

Supplementary Figure 7 Multiple sequence alignment of plum *TCP* transcription factors. Alignment of the *TCP* domain for the predicted plum TCP proteins. The basic, helix I, loop, and helix II regions are indicated. Alignment of the R domain of Class II subfamily members. The sequences were aligned with ClustalW.

Supplementary Figure 8 Expression modes of candidate *PbTCP10*, *19*, and *22* in Chinese white Pear buds treated with gibberellin. Error bars show the standard error between three replicates.

Supplementary Table 1 All TCP protein sequences list.

Supplementary Table 2 Primer sequences used in qRT-PCR.

Supplementary Table 3 Primers for vector construction.

Supplementary Table 4 Basic information of *TCP* genes in five Rosaceae species (*Prunus mume*, *Rubus occidentalis*, *Fragaria vesca*, *Prunus avium*, and *Malus domestica*).

Supplementary Table 5 Blast2GO annotation details of TCP protein sequences of six Rosaceae species (*Prunus mume*, *Rubus occidentalis*, *Fragaria vesca*, *Prunus avium*, *Malus domestica*, *Pyrus bretschneideri*).

Supplementary Table 6 Ka/Ks analysis of the *TCP* homologous gene pairs from *Pyrus bretschneideri* and *Malus domestica*.

Supplementary Table 7 All *TCP* gene promoter sequences list.
