# Supplementary figures and images for: Comparative Genomic Analysis of TCP Genes in Six Rosaceae Species and Expression Pattern Analysis in Pyrus bretschneideri
Source: Front Genet. 2021 May 17;12:669959. doi: 10.3389/fgene.2021.669959 (PMC8165447; doi:10.3389/fgene.2021.669959)

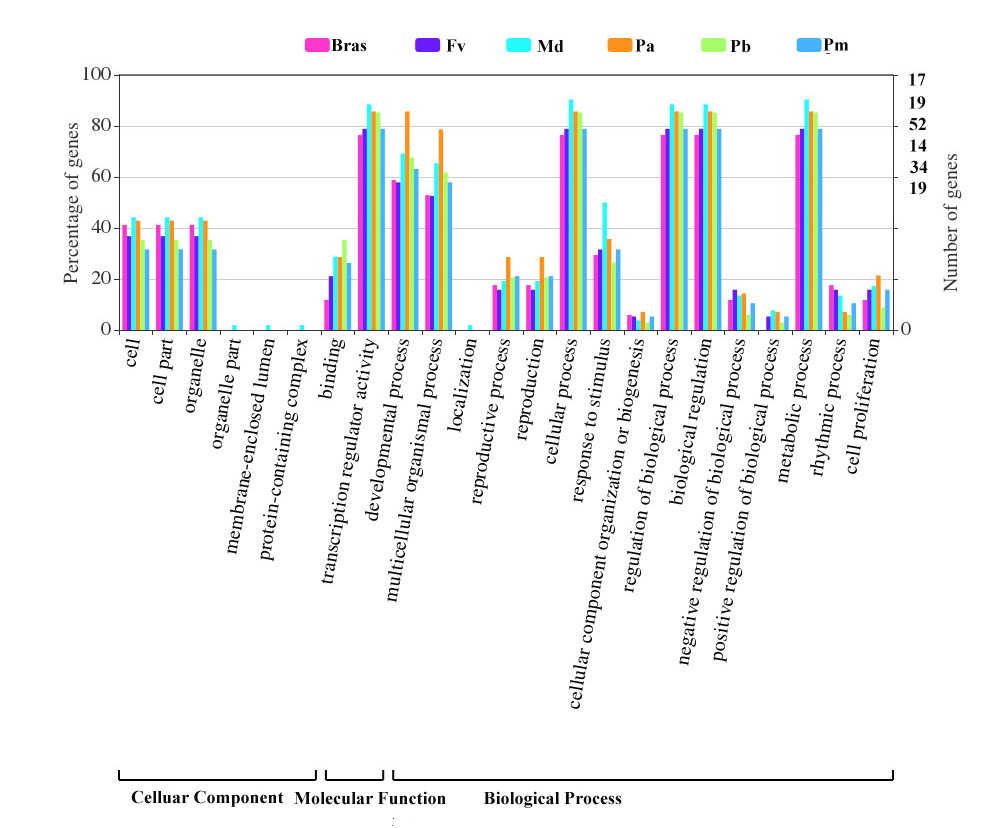

Supplement: Supplementary Figure 1 — Gene Ontology (GO) analysis of TCP genes in six Rosaceae species (Prunus mume, Rubus occidentalis, Fragaria vesca, Prunus avium, Malus domestica, Pyrus bretschneideri). [file Image_1.png]

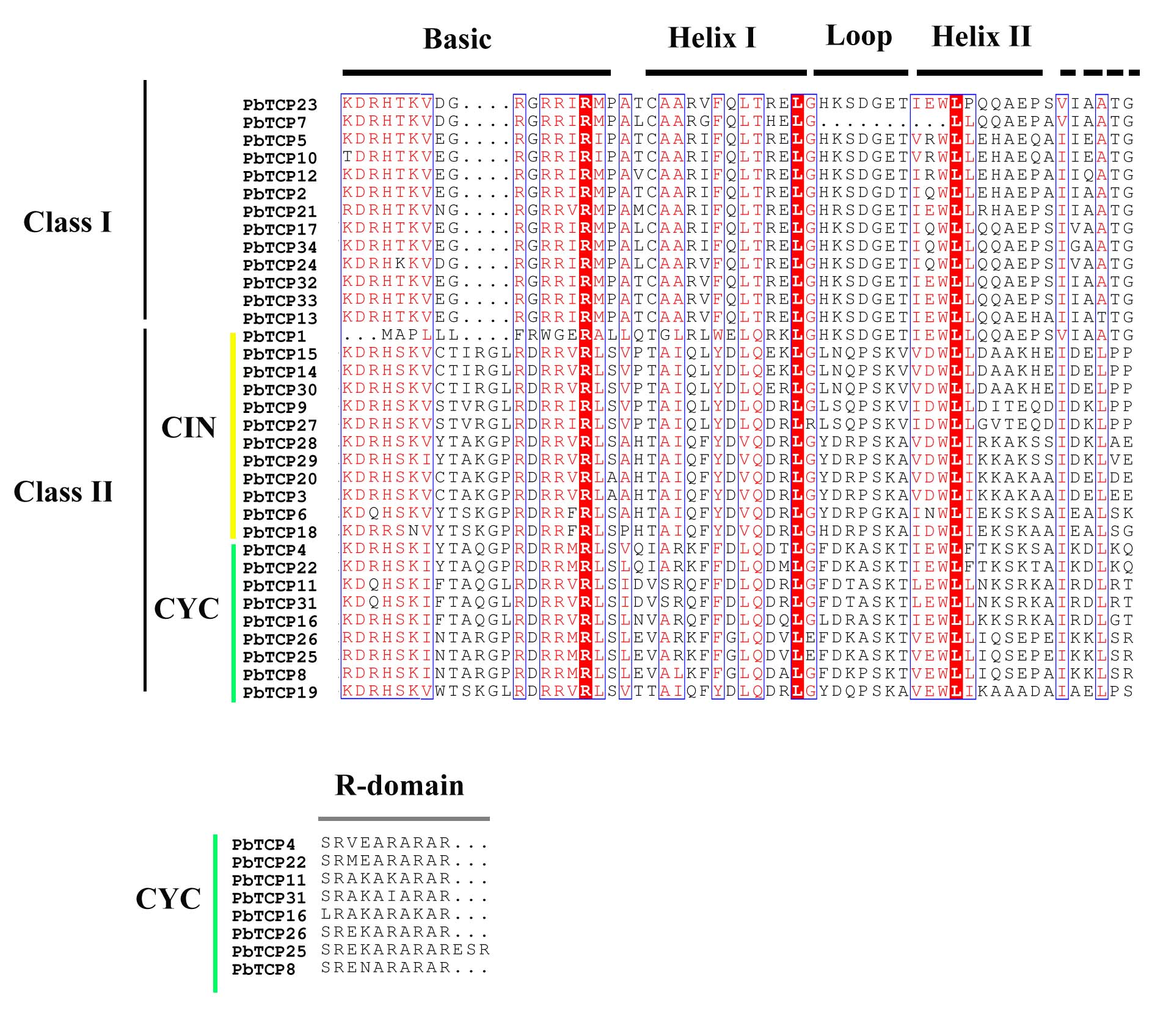

Supplement: Supplementary Figure 2 — Multiple sequence alignment of pear TCP transcription factors. Alignment of the TCP domain for the predicted pear TCP proteins. The basic, helix I, loop, and helix II regions are indicated. Alignment of the R domain of Class II subfamily members. The sequences were aligned with ClustalW. [file Image_2.jpg]

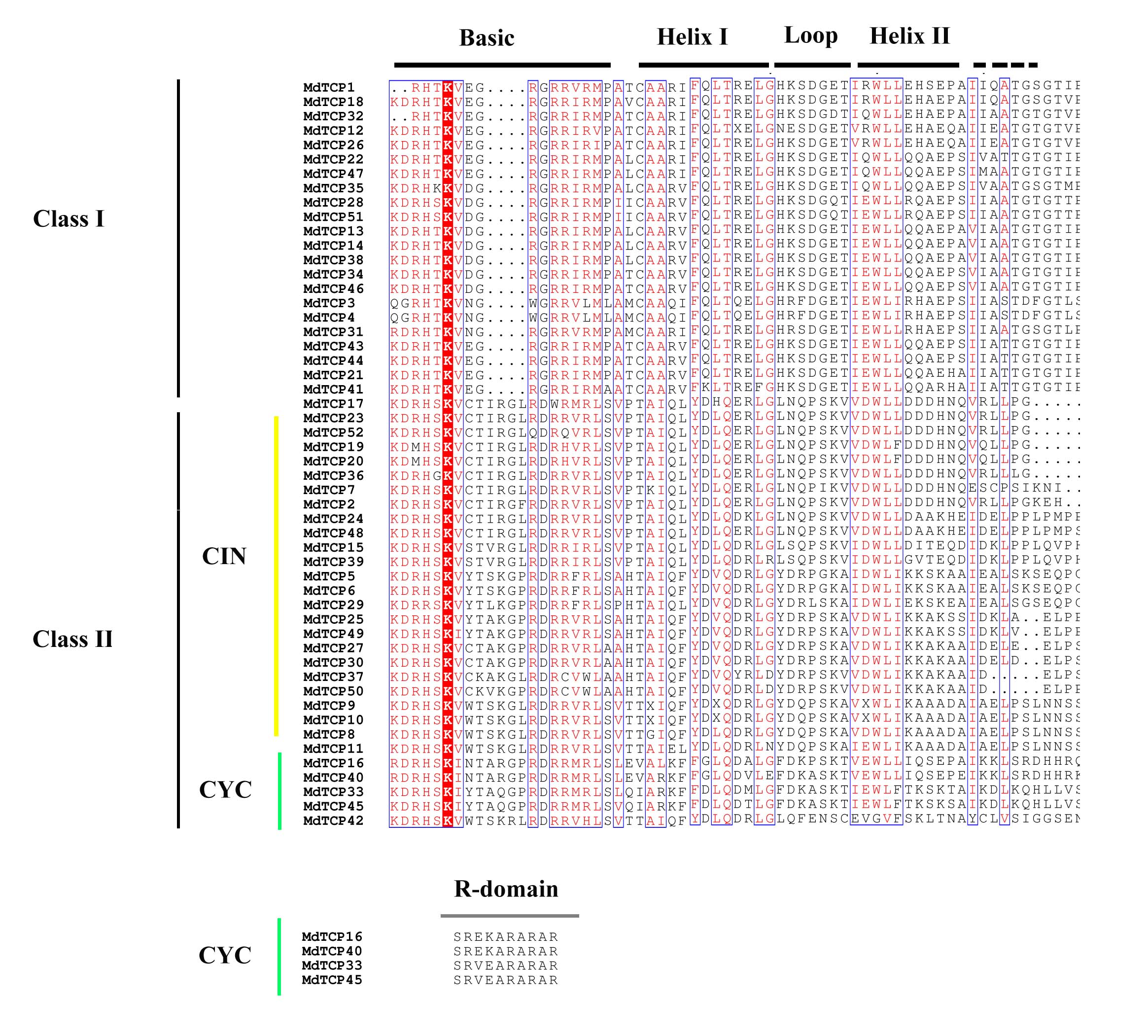

Supplement: Supplementary Figure 3 — Multiple sequence alignment of apple TCP transcription factors. Alignment of the TCP domain for the predicted apple TCP proteins. The basic, helix I, loop, and helix II regions are indicated. Alignment of the R domain of Class II subfamily members. The sequences were aligned with ClustalW. [file Image_3.jpg]

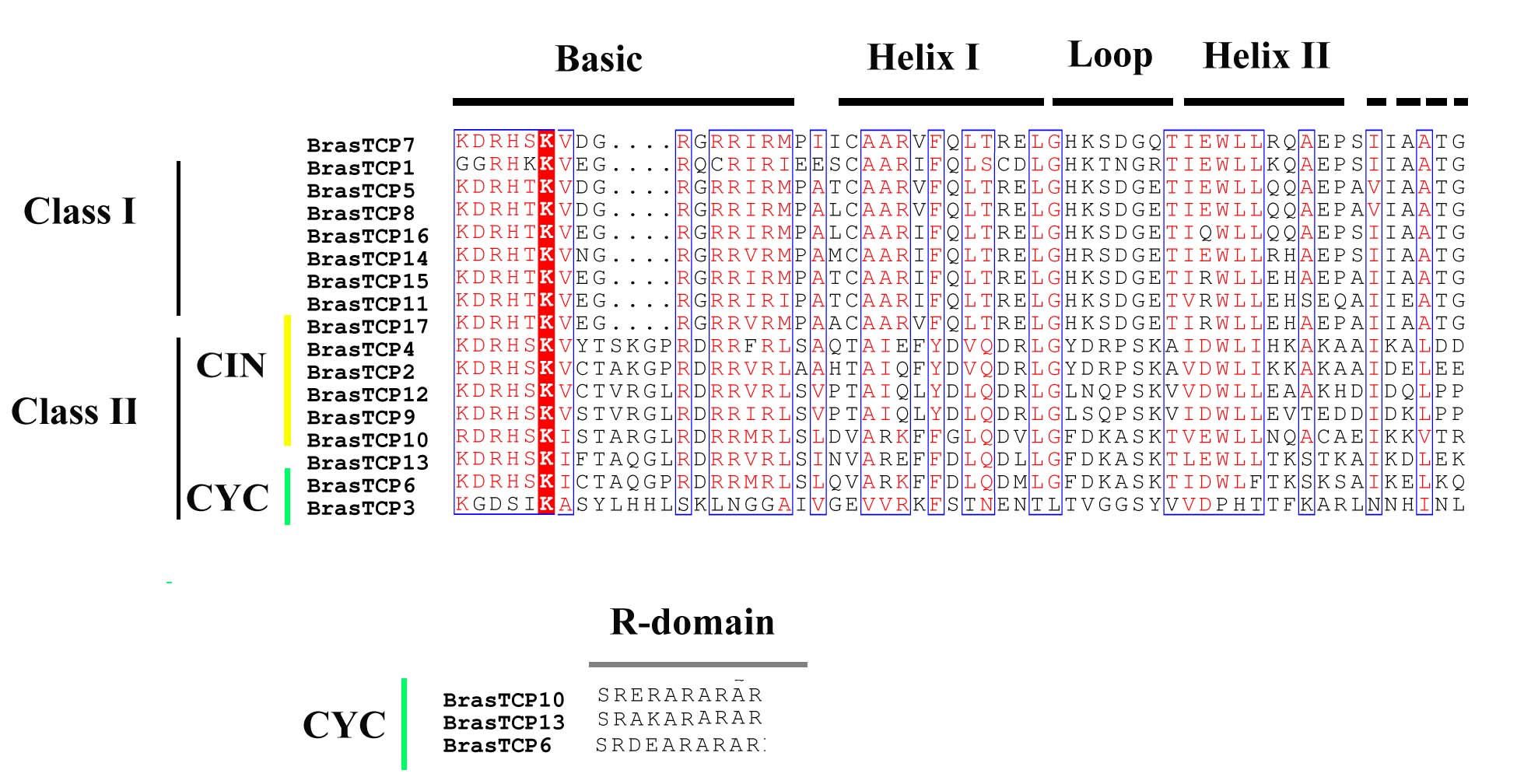

Supplement: Supplementary Figure 4 — Multiple sequence alignment of raspberry TCP transcription factors. Alignment of the TCP domain for the predicted raspberry TCP proteins. The basic, helix I, loop, and helix II regions are indicated. Alignment of the R domain of Class II subfamily members. The sequences were aligned with ClustalW. [file Image_4.jpg]

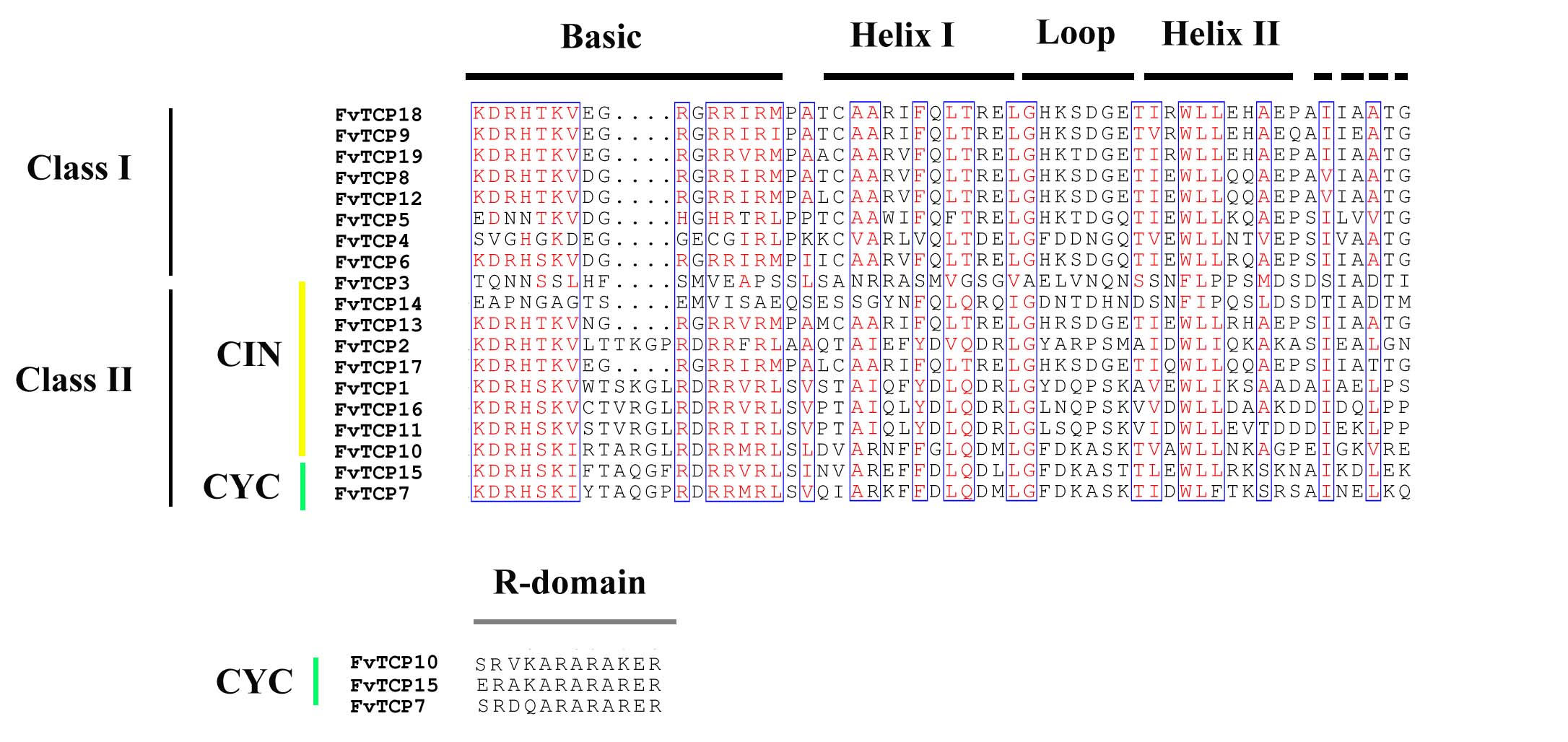

Supplement: Supplementary Figure 5 — Multiple sequence alignment of strawberry TCP transcription factors. Alignment of the TCP domain for the predicted strawberry TCP proteins. The basic, helix I, loop, and helix II regions are indicated. Alignment of the R domain of Class II subfamily members. The sequences were aligned with ClustalW. [file Image_5.jpg]

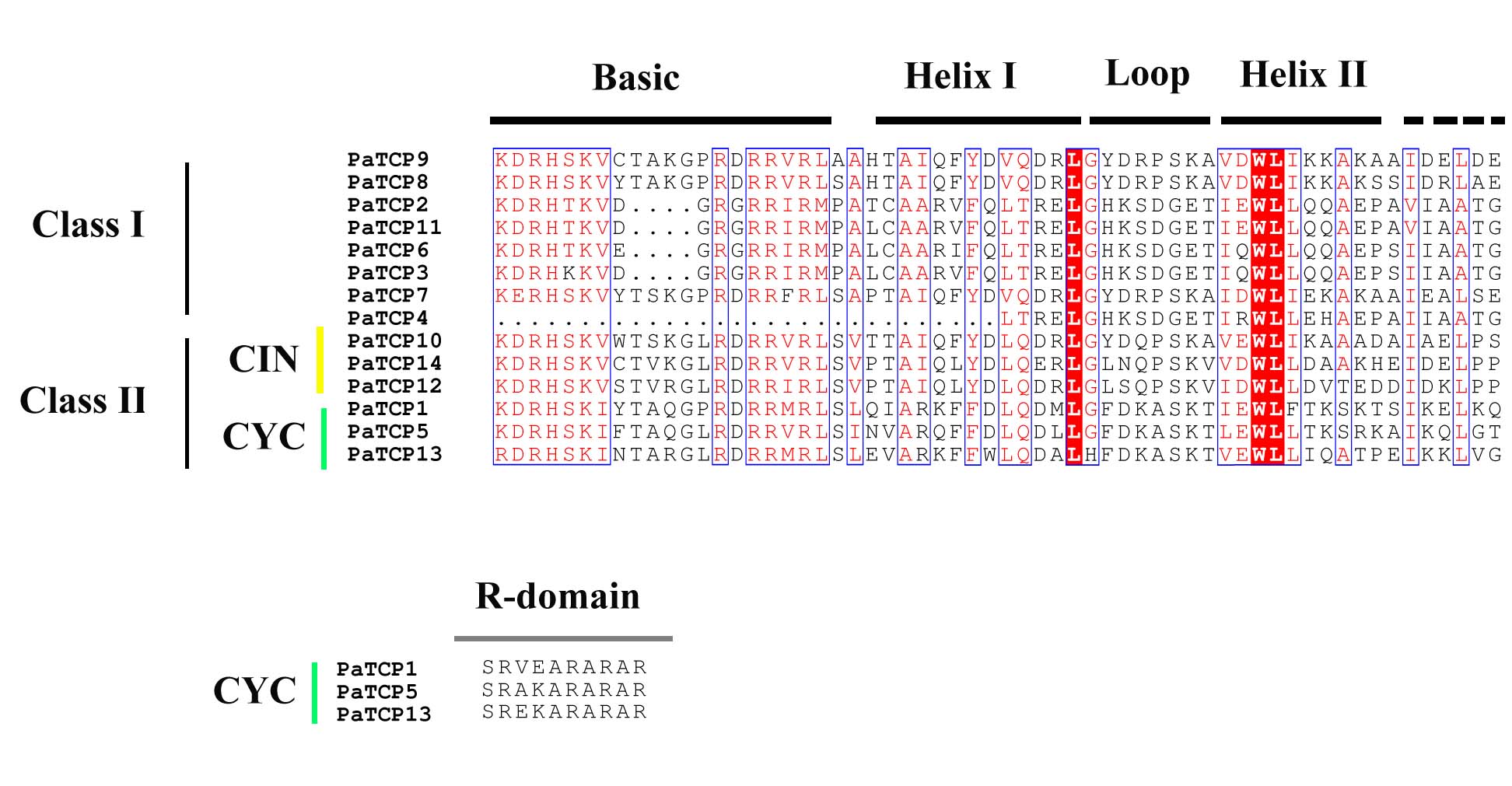

Supplement: Supplementary Figure 6 — Multiple sequence alignment of cherry TCP transcription factors. Alignment of the TCP domain for the predicted cherry TCP proteins. The basic, helix I, loop, and helix II regions are indicated. Alignment of the R domain of Class II subfamily members. The sequences were aligned with ClustalW. [file Image_6.jpg]

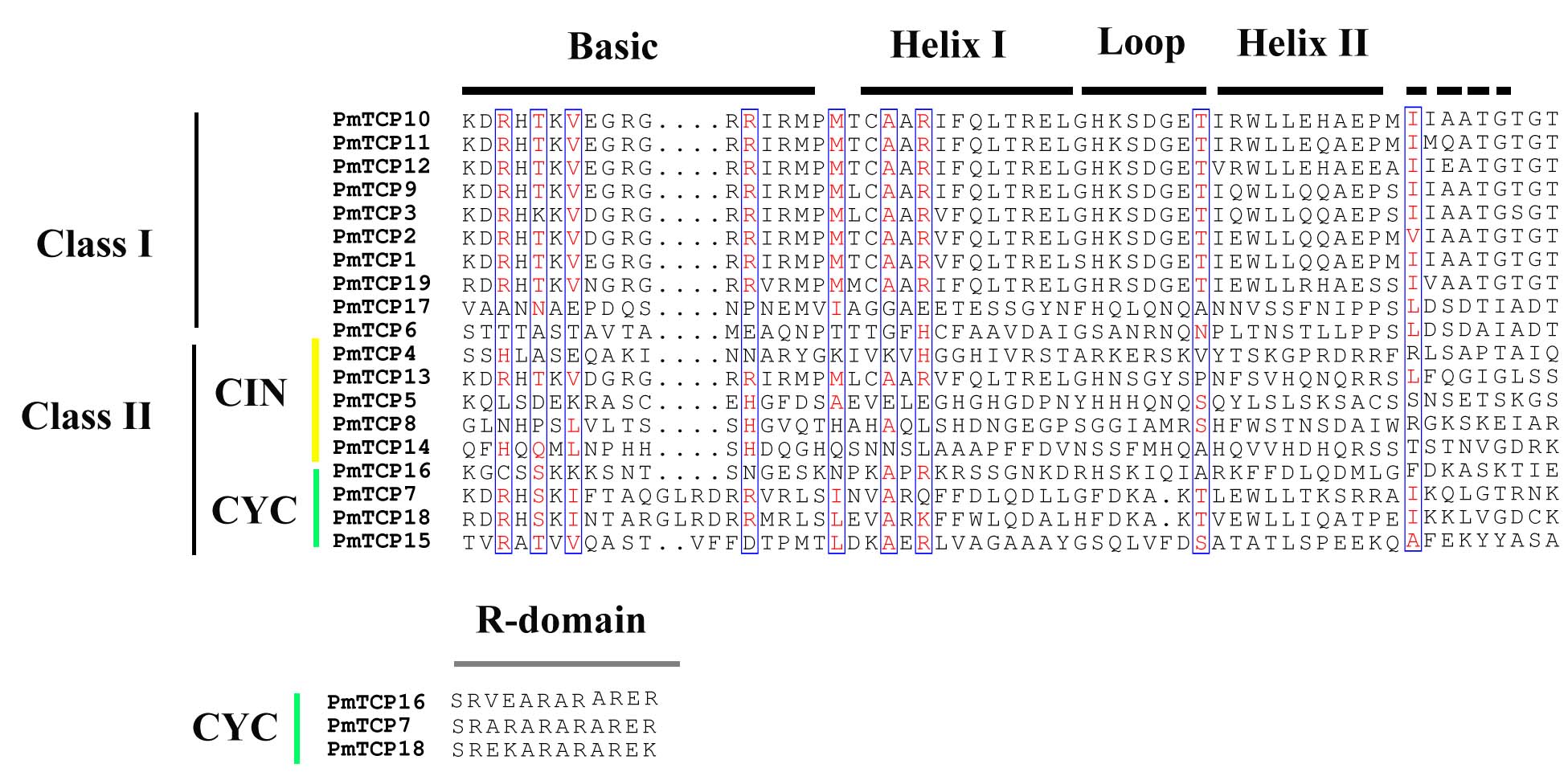

Supplement: Supplementary Figure 7 — Multiple sequence alignment of plum TCP transcription factors. Alignment of the TCP domain for the predicted plum TCP proteins. The basic, helix I, loop, and helix II regions are indicated. Alignment of the R domain of Class II subfamily members. The sequences were aligned with ClustalW. [file Image_7.jpg]

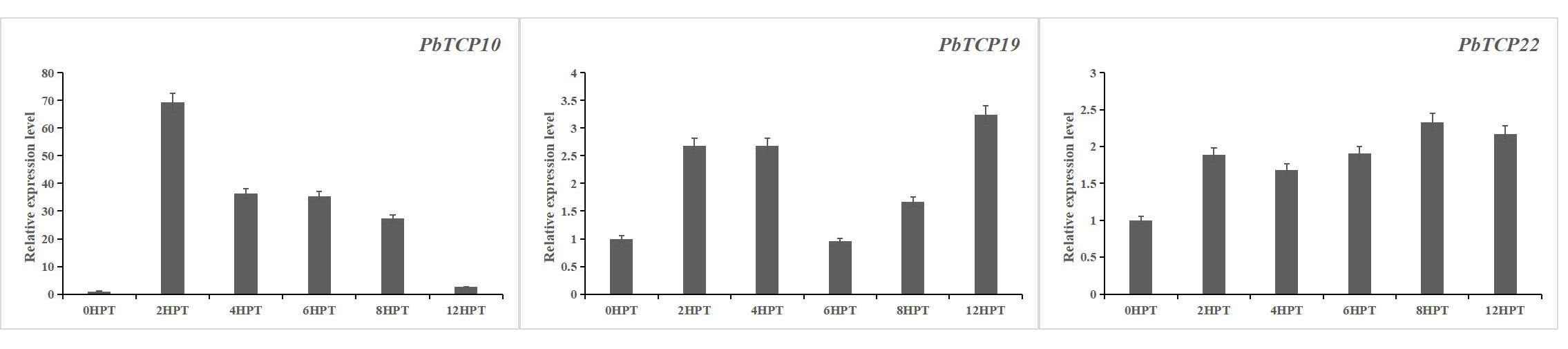

Supplement: Supplementary Figure 8 — Expression modes of candidate PbTCP10, 19, and 22 in Chinese white Pear buds treated with gibberellin. Error bars show the standard error between three replicates. [file Image_8.jpg]
