# Supplementary material for: Comparative Genomic Analysis of TCP Genes in Six Rosaceae Species and Expression Pattern Analysis in Pyrus bretschneideri
Source: Front Genet. 2021 May 17;12:669959. doi: 10.3389/fgene.2021.669959 (PMC8165447; doi:10.3389/fgene.2021.669959)
Supplement: Supplementary Table 1 — All TCP protein sequences list. [file Table_1.docx]

| *Gene ID* | Protein sequence |
| --- | --- |
| *PbTCP30* | MIKSPISEADLQEAGAGSSSRDDQANKISSNPNLSRPSTPWLRLKDPRIVRVSRAFGGKDRHSKVCTIRGLRDRRVRLSVPTAIQLYDLQERLGLNQPSKVVDWLLDAAKHEIDELPPLPMPPSGSFGLNHPSLGLTSSHGDQSNAHAQLSHNHSGEGPRSAIDRSNFWPTGSDALWRAKSKEIVRDTRNEDEGNQKDNLGISDDQEQAGNNVDGNSSNTFLRSNTNPPFFPGLLNSSTNMPYAYHNWDHSHQTSNFPLSQLGSHGFQSETTDLHNFRNVLSLPSTLSLSTTQSYFPSHNTAATGEMDPRQFNHLQMLNSSSSTRTSQNLLPNFLSTPALYPSSQTLRAPHLSMVTKLVHSSDNTGSGHHPNNNQEPPSR |
| *PbTCP15* | MIKSLVSEADLKEAGAGSASRDEQANKISSNPNLSRSSTPWPRLKDPRIVRVSRAFGGKDRHSKVCTIRGLRDRRVRLSVPTAIQLYDLQEKLGLNQPSKVVDWLLDAAKHEIDELPPLPMPPPGTFGLNHPSLVLTSSHDDQTNAHPQLSHNDRGEGPSSGIDRSNVWPTNLDALWRAKSKEIARHTRNEEEEGKRKDNLGISDDQKQAGNNIDGNSSNTFLTRGSTTNPPFFPGLLNSSTNMPYDYQNWDHPHQTSSFPLSQLGSHGFQSQTTDLHNFLNVLSLPSTLSLSTTQSYFPSHNAAATGEIDPRQFNHLQLLNSSSSTSTSQNLLPNSLSTPALYPSSQTLRAPHLSMVTKLVHSSNNTGSGHQPNNDQGPLHR |
| *PbTCP14* | MIKSLVSEADLKEAGAGSASRDEQANKISSNPNLSRSSTPWPRLKDPRIVRVSRAFGGKDRHSKVCTIRGLRDRRVRLSVPTAIQLYDLQEKLGLNQPSKVVDWLLDAAKHEIDELPPLPMPPPGTFGLNHPSLVLTSSHDDQTNAHPQLSHNDRGEGPSSGIDRSNVWPTNLDALWRAKSKEIARDTRNEEEEGKRKDNLGISDDQKQAGNNIDGNSSNTFLTRGSTTNPPFFPGLLNSSTNMPYDYQNWDHPHQTSSFPLSQLGSHGFQSQTTDLHNFLNVLSLPSTLSLSTTQSYFPSHNAAATGEIDPRQFNHLQLLNSSSSTSTSQNLLPNSLSTPALYPSSQTLRAPHLSMVTKLVHSSNNTGSGHQPNNDQGPLHR |
| *PbTCP9* | MITNSRDKGFQAKQEGHNNNNNNDGNNNSNFHKASSSSTTTSRQWSGFRNPRIVRVSRTFGGKDRHSKVSTVRGLRDRRIRLSVPTAIQLYDLQDRLGLSQPSKVIDWLLDITEQDIDKLPPLQVPHGFGHQFHQPMLNPHKASNSLVAPFFDVNSTFMEADQVDHQEVHDQAKGKSIRTNDGQDDQNRHDHEGNVSGQLLAQKLFPIGNHPSSIPGLLNNAMAYNYYHNYSEPSTLSLAQFGSHEFPQVPQIDQHHSHMMSTNALSFSTPLPSVSQLFFCPPTATPTLFGSYPPYITNPIVEGTSTATDQPRQANHFQFLSSPSNSQNFLANNALMPPLHSVSSSLKSFPSLVHPKQQLHLNSQNNNGSQPNKDVP |
| *PbTCP27*  *PbTCP19* | MITNSRDKGFQEKQEGHNNNDGNNNSSFNKASSSSTTTSRQWSVFRNPRIVRVSRAFGGKDRHSKVSTVRGLRDRRIRLSVPTAIQLYDLQDRLRLSQPSKVIDWLLGVTEQDIDKLPPLQVPHGFGHQFHQPMLYPHQASNSLIAPFFDVNSTFMEADQVDEQVRDQAKGKSIKTNDEQDDQNHHDHEGNVSGQLLAQKLFPIGNHPSSIPGLLNNAMAYNYYHNYSEPSTLSLAQFGSPGFPQGPQTDQHRSHMMSTNALSFSTPMASGSQLCFCPPTATPTLFGSYPPYMTNPIVEGTSTATDQPRQASHFQFLSSSSNSQNFLANNALMPSLHSIGSSLKSFPSLVDPKQQLHLDSQNNNASQPNKDVP  MEVEEIQASNKFPRIGNGSSRDHKPSPEDEDNQDPSCLDLKRAAAATAAADAGNRLRGWHHSRIIRVSRASGGKDRHSKVWTSKGLRDRRVRLSVTTAIQFYDLQDRLGYDQPSKAVEWLIKAAADAIAELPSLNNSSFPDTPKQLSDEKRASCERGGFDSAEIEFDQNYHQNQSQPGNQSQHLSLSKSACSSTSETSKGSGLSLSRSEIRVNRSKARERARERAAKDKEKESIESTYPGNINNISQQNGSFTELLTAGIGNIHNNNSPTASAQHHQQNHGGGGEPILFHKAAAPMDYFSPGLLGLSSSARTHHSSGFSGQIHLGMNSIPQTMSVVSPFNVSGEHHHGHHSSELQHFSFVPDLIPVTTSSQPGSGVDYNLNFSISSSGGLAGFNRGTLQSNSSSSPSLLPHHLQRFSPIDGSSNVPFFIGAAAAAASPTMENHHHHHHHQQHQQQFPGGFDRRLQQLYGDGTRHSDHKGKAKN |
| *PbTCP28* | MGMKGCGGEIVQVQGGHIVRSTGRKDRHSKVYTAKGPRDRRVRLSAHTAIQFYDVQDRLGYDRPSKAVDWLIRKAKSSIDKLAELPPWHPITTSNHTAEADDPFRSSNPNPNEMVIAAAEPQSESSAGYNFNFELHRQRQSDNDSNFNIPPSLDSDNIADTMKSFFPTNTSSNAAAASSVVDFQSYAPDPHLISTTTQDLGLSLHSFQDQSLNIHHNHTHTQQSSQTLFAAAMGIGLDSSSYQRMVAWSNENRGLDGGFVFNSHSHSYAQPQPHHHGGTLQSSFTPSVSARTWQQQQQHSSIFGTRFGASDGSSPVFCIQGEEAENGVVSDRTASTSSPNSTRQL |
| *PbTCP29* | MGMEGCGGEIVEVQGGHIVRSTGRKDRHSKIYTAKGPRDRRVRLSAHTAIQFYDVQDRLGYDRPSKAVDWLIKKAKSSIDKLVELPPWHPITTSNHAAEADDPFRSSNPNPNDMAIAAAEQQSESSSSYNFNFELRRQRQSDNDSNFNIPPSLDSDNIADTMKSFFPTNTSSNAAAASSVDDFRSYPTDPHLISATTQDLGLSLLSFQDQGLNIHHNHTHSHTQQPSQALFAAAAATGFDSSSYQRMVAWSNENRGLDGGFVFNSHSHSYAQPQPHNHGGDGNHGSTLQSSFTPSVSTSAWQQQHSSIFGTRFGTSDGTSPAFCIQGDEAENGVISDRPSSTSSLNSTRQL |
| *PbTCP20* | MGDSHHHQATTSSRLGIRPPSSGGGVVSTDIVEVVRGSHIVRATGRKDRHSKVCTAKGPRDRRVRLAAHTAIQFYDVQDRLGYDRPSKAVDWLIKKAKAAIDELDELPSWNLYSTSTTASVPGVETHNPTTTGIHCFAGVDAIGSANRRTTMVGSGVSEQQIVQNPNSNLTFLPPSLDSDAIADTIKSFFPTGASAIAAAAEAPSSTIQFHQNYPPDLLSRTSSQSQDLRLSLQSFQDPILLHQHHAHTHQNEQSLFSGSQNPLGFDGSSAAWAEHHHNQQQQEINRFQRMVAWNSGDGDSGDNGGGSSSSGGFTFNSLLPTQQSSTSSLQQQQSLFGQSQFFFQRGPLQSSNSPSIRAWMMDQQNQQSISHDHHHQISQSIHHQPSFSSMGFTSGGEFSGFHIPARIHGEEEHDGISDKPSSASSNSRH |
| *PbTCP3*  *PbTCP6* | MEDSHHHHLNNHQATTSSRLGIRPPSSGGGVGTDIVEVVRGSHIVRATGRKDRHSKVCTAKGPRDRRVRLAAHTAIQFYDVQDRLGYDRPSKAVDWLIKKAKAAIDELEELPSWNPHSISTTAAVPAMETQNPSATGIHCFAAVDAIGSANRRTMVGSGVSEQQFAQNPNSNSTFLPPSLDSDAIADTIKSFFPMGASEATAAAEAPSSTIQFHQNYPPDLLSKASSQSQDLRLSLQSFQDPILLHHHDSQNQHHHAQTHQNEQTLFSGSQNQLGFDGTSGSWAEHHHNQQQQEMNRYQRMVAWNSGGGDSGGNGGPSSAGGFIFNSLLPTQHGSTSPLQQQQQSLFGQSQFFSQRGPLQ  SSNSPSVRAWMMDQQNQQSISHDHHHQISQSIHHQQSISGMGFASGGGFSGFHIPARIHGEEEHDGTSDKPSSASSNSRH  MPTSKSAFKQPRVDNARYGKIVKVHGGHIVRSLARKDQHSKVYTSKGPRDRRFRLSAHTAIQFYDVQDRLGYDRPGKAINWLIEKSKSAIEALSKSEQPCQEYNDCTNTNVFGQQTEQEIGEQSMHQFWNHPESNGELETMSNVSPMNNVNNLKEPVFDPHQLSSLNYAEGALDSASSLSDSKFKEMGWFQSLVAWNYNANDGGEICPYNSSHVSLH |
| *PbTCP18* | MATSKSAFKQQQVDNTRYGKIVRVQGRHIVRSMARKDRRSNVYTSKGPRDRRFRLSPHTAIQFYDVQDRLGHDRPSKAIDWLIEKSKAAIEALSGSEQPCQYYYDCTNSNVLGQQTEQEIGEQSKHQFWNHPESNRELGTMSNGSPMNNVNNFKEPVFDPHQLSSLNYAEQALDPTSSLSDSKVTEMGWFQSLVAWNYNAGDGGKICPYNSSHVSLQ |
| *PbTCP4* | MFPSSSTTSNFQLPFLCDDNQTNAIIDDFHQNPNSILSHGGHHQYYSQYYYSEDQQQQAPDFLEHDGMLLSYLLSQQQLLLGNSSTTTNVATDNNHTRAHESTEISIAASNSNKAMVVINRDDDDGRNELTVNTAVASKIKKNNVKTSGGGGGGEKKAKVARKRASGKKDRHSKIYTAQGPRDRRMRLSVQIARKFFDLQDTLGFDKASKTIEWLFTKSKSAIKDLKQHLLVSPKEDYSSTNGGATAKVNSSENTTGEVTSRIMEPSSSAANGDISVGFGREKRNRKLCVVARESRVEARARARERTREKMMRIRGFDQDHQHLTKQSPNHHEHQNPNELFETAGMMNSAMMSTNCCNDGQKIVGRTSSSCGGAQRSFLNFDFWRHVSEASRANSEDCGFPGNWGAINYTKNIITGNVLQVEQNPTSYPKQQNPRSIFGTGTQEQNPNSSFLTTFIKAQDP |
| *PbTCP16* | MFSSSSYNSNTVSPFQHNFPFSSSSSSNYPPPPPPPCANLETCTGHTFLHHIPDPLSGQFSHQNIALLAPPSHQTLTHLGVSSNTVPGSINSNISSSCGDHHHRQYYHSNYGSISNDNIIPYFLHSSGEDVVVAPAATKKDRHSKIFTAQGLRDRRVRLSLNVARQFFDLQDQLGLDRASKTIEWLLKKSRKAIRDLGTQKKNLSCSSERSKSLSSTSECDEVVSDINEVENHVLSKEKEMVMMKKLKESKSAGAYVAKELRAKARAKARERTREKKMCTTSRRPHQMLNQLNLMIEQLDDHETNISSSSKVNFGDHRVNQELGSLLAKQAHHHHRHEEDQSALIKRNKMKSFSSVVSDYNTTDQISSNGHLQFPNASQNWDINGAFPMP |
| *PbTCP22* | MFPSSSTTSNSQLPFLCDDSHTNGIIDDFHQNPNSISSHGGHHQYYSQYYYSDDQQQQQAPDFLEHDGMLLSYLLSQQQLLIDNSSTTNVATHSNHIRAHSSAKISIVDSNSNKTMELINHADQITVIPAADSQIKKNNVKMNGEGGGEKKAKFARKRASGKKDRHSKIYTAQGPRDRRMRLSLQIARKFFDLQDMLGFDKASKTIEWLFTKSKTAIKDLKQHLLVSPKEDYSSTNGGATAKVNSSESTTGDVASKIMEPYSSAAKGDISVGLAREKKNRKLSVVARESRMEARARARERTREKLMRIRGFDQDHQSTKQSHKHQEQQNPNELFETAGMMNSMMMSMNRPNDGQKIVGRTSSSCGGAESSFLNFAFSQHAEASGANSEDCDFPGNWGAINNTKNIITGNVLQVVQNPTSYPKQQNPSSIFGTGTQEQNPNSIFLNTLAKAQDQNSTTSSNFEINSNISTLQPLFTGN |
| *PbTCP11* | MFSSSSCNSNTVSPQPNFPFSSTNYHPHPPPPPPPPPPCVNLVTCTGDILFHHIHDSLSG  QFSHQNISLMAPSAHQTLTHLGVSSNTVPPSINAPVSSSCGDLHHHQYSNYVLHSSRQDVVVAPAKKDQHSKIFTAQGLRDRRVRLSIDVSRQFFDLQDRLGFDTASKTLEWLLNKSRKAIRDLRTRKNNLSCSSERSKSLSSTSECKDVVSDINEVDNHVVSKEKEMVMMKLKESESAGAYVAKESRAKAKARARERARAKKVCITSRRPQQILNQMNLFNEQLNDHETNISSSSKVNFGDHHVNQELGSLLDNQAHHHHFQEEDQSALIKRNKMKLISSVVSNYNTTDQSSSNSYLQFTNSSQYWDINGAFPMP |
| *PbTCP31* | MAPSAHQTLTHLGVSSNTVPPSINAPVSSSCGDLHHHQYSNYVLHSSRQDVVVAPAKKDQHSKIFTAQGLRDRRVRLSIDVSRQFFDLQDRLGFDTASKTLEWLLNKSRKAIRDLRTRKNNLSCSSERSKSLSSTSECKDVVSDINEVDNHVVSKEKEMVMMKLKESESAGAYVAKESRAKAIARARERARAKKVCITSRRPQQILNQMNLFNEQLNDHETNISSSSKVNFGDHHVNQELGSLLDNQAHHHHCQEEDQSALIKRNKMKLFSSVVSNYNTTDQSSSNSYLQFTNSSQYWDINGAFPMP |
| *PbTCP8* | MFPSHNNNLNELVPVSYPHVDQSIFHSWPFHHDNSTLTPNSLTILNPNPNSRQQEGGEENLHQQHQHLPFSLLYFPSPFEDDDVLLFDQQHHHQEPDHIELSLHESQEPPYFMKEAAAAAADDNTVVGNDHQKTTTTSVNRKMVDWDSNKKGHRMNMDDQPQIPRRRTCKRDRHSKINTARGPRDRRMRLSLEVALKFFGLQDALGFDKPSKTVEWLLIQSEPAIKKLSRDHHRQFNYKHMVRCAKTTSPATSESCEVLSGVDEAPTNVNISSNGKVRSRGIKPSAKERKIVHRQSRKSAFHPLAKASRENARARARERTREKMQRSKKPSNDQANSSRLSSWNPFETEEESPIHNNNMTSTTNDQPNSRAVLRDYPDEVEEPLSSSQAGDIQDMVVDHGTTHDAMVVLGKWSPPPVFTRLQQNTGISQEHQQFADFQFFGKPWEVDNNTHNQSILSGNFLS |
| *PbTCP26* | MFPSDNTDNVNELVPVSYPHVDQPFFHSRPFDHDITTVTPNFFTNSNPNPNSGQQRGGEENLHQQHHHPPLSLLYFPSPFEDDDVLLFQQHHHQEPDHIEISLHESQVPPYFMKEAAAAATATAAAAATAADDNTGTVVGDDHQKTTTTSVNIKMVDWDSNKNGHHMDMDDQPQIPRRRTNKRDRHSKINTARGPRDRRMRLSLEVARKFFGLQDVLEFDKASKTVEWLLIQSEPEIKKLSRDHHRKFNYKNMVRCAKTTSPATSESCEILSGVDEAPTNINISNGGNDDNDKVRSCGIKPSAKERKIVHRQSRKRAFHPLAKASREKARARARERTREKMQRSKKPSNDQAKLSRLNSWNPFESGEESSAYNNNMNNTANDQPDSLVALRPYPDGVEEPLSSSLAGDIQDMVVDHGATHDAMVVMGKWSPSSVFTPLQQNTGISQEHQQFADFQFFDKPWDVYNNTHKLF |
| *PbTCP25* | MFPSDNTDNVNELVPVSYPHVDQPFFHSRPFDHDITTVTPNFFTNSNPNPNSGQQRGGEENLHQQHHHPPLSLLYFPSPFEDDDVLLFQQHHHQEPDHIEISLHESQVPPYFMKEAAAAATAHAAAAATAADDNTGTVVGDDHQKTTTTSVNIKMVDWDSNKNGHHMDMDDQPQIPRRRTNKRDRHSKINTARGPRDRRMRLSLEVARKFFGLQDVLEFDKASKTVEWLLIQSEPEIKKLSRDHHRKFNYKNMVRCAKTTSPATSESCEILSGVDEAPTNINISNGGNDDNDKVRSCGIKPSAKERKIVHRQSRKRAFHPLAKASREKARARARESRQSRKSAFHPLAKASRENARARARERTREKMQRSKKPSNDQAKLSRLNSWNPFESGEESSAYNNNMNNTANDQPDSLVALRPYPDGVEEPLISSLAGDIQDMVVDHGATHDAMVVMGKWSPSSVFTPLQQNTGISQEHQQFADFQFFDKPWDVYNNTHKLF |
| *PbTCP5* | MTSYLEDQDDDGGASDLSTSTGDPGDNDHTNGDGVVSTQPNYEETTELHSLKEEPIDSDPQQPPQGHPIGMLPDTMQMPMPMSVQLPVSAPTRRSSTKDRHTKVEGRGRRIRIPATCAARIFQLTRELGHKSDGETVRWLLEHAEQAIIEATGTGTVPAIAVSVGGTLKIPTTSSMSVEDNSSPTSATKKRKRPSNSEFMDVRKDAVSQSSGLAPVSPTAPQGLVPVWAVGGAGLIVPANAFWMGPVGSGAAPSGPQPQIWALSPTMTPVFNMVGAARPISSFVVNSGGGAGGVEVRAPSPALSNSAASTSTVGARAAKRSSTTMAPPSVSSSSNNSNGSGSGATTKTQMLRDFSLEIYDKQELKFMDQPVGPAHQQQTQ |
| *PbTCP10* | MTSYLEDQDDDGGASDLSTSTGDLGDNDHTNRDGVVSKQLHYDETTEFHSLKEEPIDSDPPQPPQAYPIGMVPVAMPMPMSVPSAVSTLTRRSSTTDRHTKVEGRGRRIRIPATCAARIFQLTRELGHKSDGETVRWLLEHAEQAIIEATGTGTVPAIAVSVGGTLKIPTTASTSVEDNSLPPSSTKKRKRPSNSEFVDVKNDAVSQSSGLAPVSPSVPQGLVPVWAVGGTGLMVPANALWIGPVGSGAGPSGQPQIWALSPTMTPVFNMAGAARPISSFVANSGGGGAGVVDDRAPSPALSNSAANTSTVGPRAAMWSSTTMAPSMSSSSHNSNGSGSGATTKSQMLRDFSLQIYDKQELQLMGRPVGPENHNQTQ |
| *PbTCP13* | MDLSNFQPNNNKKQQQQQTNNTSTSTSNNNNTSLQHHHLLPHLQQKFQHHHSSSSSSSSSSSSHFVGPFDGIRSSSGGGGGGGGPPFVGSISIQAGLTTTSHSTPPPPPTSVPSSSSFSS  SSSTSSPSSTSTSTAPPPHLVDASLAIATRSDPHPPTRPASLLDSAKNNQTQQQLTISPATTTAPSAPAAPATQVAKRSTKDRHTKVEGRGRRIRMPATCAARVFQLTRELGHKSDGETIEWLLQQAEHAIIATTGTGTIPANFSTLNISLRSSGSTLSAPPSKSSPHSFPGALALAHHPHYDEGFAHSGLLGFHQQHQQQQHQQHHHQPHQSPLITADQIAEALPSGGGDSAGGGGGESTENYMRKRFREDLFKDDNQGQGESGSGDGGGSPSRNKAFKGASSSGLQLPKQQQQGAEVG  SSGVLRPSNILPATAMWAVAPAPTSGAGNTFWMLPVTAGPGVPSMATATFGAGPSEAAQMWTFPSSTSSHGSTLQAPMHFMPRFNLPGSLEFQPAAAGGRANPLQLGSMLMQQPPSQHLGLGVSESNLGMLAALNAYSRGGLNMNSDQHQQNHHHPLDHHQQQQHQPQTTDSGDEAPNSSQ |
| *PbTCP2* | MDMIQEEQQHERDRDQDEDASAADLGIDGGGESDPEDPTDPSRALALPPKQEQDVPMTVAVHAPRRASTKDRHTKVEGRGRRIRMPATCAARIFQLTRELGHKSDGDTIQWLLEHAEPAIIQLTRELGHKSDGDTIQWLLEHAEPAIIAATGTGTVPAIAISVNGSLKIPTDVRPGDDPPDQKKRKRNSNSEYVDLNDGVSVSAGLAPLTTEGRRQPPPPQPPTTAVQAVAPQGMVPMWAIPSNTVVPGAFFMVPSTTQPHIFTFPTTLPAAPFINISARPISSFVGPSSAAPPQPLQRAVAAALRR |
| *PbTCP32* | MDLSNFQPNNNKKQQQQQQQNNNTSTSTSNNTTTSLHHHHLLPHLQQKFQHHHQQQHPSSSSSYSSSFVGPFDGIRSSSGGGGGGGGPRSMSSISIQAGLTATSQSTPPPPTTSAPSSSSFSSSSSTSSPSSTSTSTAPPQLVDASLAMATRSDPHPPTRPGSLLDSAKNNQIQQQLTISPATTPPPAAAATTTPVAKRSTKDRHTKVEGRGRRIRMPATCAARVFQLTRELGHKSDGETIEWLLQQAEPSIIAATGTGTIPANFSTLNISLRSSGSSLSFPPSKSAPHSFHGALALAHHPHYDEGCAHSGLMGFHQQLQQQQHPQHHHQPHQSPLMTADQIAEALPSGGRDSGGGGGGESTENYMRKRFREDLFKDDNQERGESGSGGGGGSPSRNKAFKGGSSCGLQLPKQQQQGAELGSYSSGVLRPSNILPATAMWAVAPAPTSGAGSTFWMLPVTAGSAVPSMATGTSGAGPSEAAQMWTFPSATASHVSTLQAPLHFMPRFNLPGSLEFQQAAAGSRGSPLQLGSMLMQQQPSQHLGLGLSESNLGMLAALNAYSRGGLNMNSDHQQSHHHPLDHHQQQHHQHQTTDSGDEGPNSSQ |
| *PbTCP33* | MDLSNFQPNNNKKQQQQQQQNNNTSTSTSNNTTTSLHHHHLLPHLQQKFQHHHQQQHPSSSSSYSSSFVGPFDGIRSSSGGGGGGGGPRSMSSISIQAGLTATSQSTPPPPTTSAPSSSSFSSSSSTSSPSSTSTSTAPPQLVDASLAMATRSDPHPPTRPGSLLDSAKNNQIQQQLTISPATTPPPAAAATTTPVAKRSTKDRHTKVEGRGRRIRMPATCAARVFQLTRELGHKSDGETIEWLLQQAEPSIIAATGTGTIPANFSTLNISLRSSGSSLSFPPSKSAPHSFHGALALAHHPHYDEGCAHSGLMGFHQQLQQQQHPQHHHQPHQSPLMTADQIAEALPSGGRDSGGGGGGESTENYMRKRFREDLFKDDNQERGESGSGGGGGSPSRNKAFKGGSSCGLQLPKQQQQGAELGSYSSGVLRPSNILPATAMWAVAPAPTSGAGSTFWMLPVTAGSAVPSMATGTSGAGPSEAAQMWTFPSATASHVSTLQAPLHFMPRFNLPGSLEFQQAAAGSRGSPLQLGSMLMQQQPSQHLGLGLSESNLGMLAALNAYSRGGLNMNSDHQQSHHHPLDHHQQQHHQHQTTDSGDEGPNSSQ |
| *PbTCP21* | MASEMALQPLHKLEDRKPTVLPTAVLTSATIQTAPSLSQLSDLQLQAQPKPQKQVSRRNTRDRHTKVNGRGRRVRMPAMCAARIFQLTRELGHRSDGETIEWLLRHAEPSIIAATGSGTLPAEPISTTSPSVSSQMPSVTCRVQPLKSLRSSGQFLYPAAAQAHQAMQLQLDLCQPMGADYNAGYRHMPFTSLLLQTGESEVEEGQAEEEVVGVGIGDDE |
| *PbTCP24* | MAENNKAPAQIKDLQIMIANKEKDESKKQLAPKRSSTKDRHKKVDGRGRRIRMPALCAARVFQLTRELGHKSDGETIQWLLQQAEPSIVAATGSGTMPASALAAAAESSVSEQGNSVSSGLMSFGHLGRSNAATGIWPFVSGIRSSFDHNSGQQTLNFGGENLNFMHKFGFQGFDAPSNLGSMSFPTTLSGSNQQVPGLELGLSQDGHIGLVNSQALSLTQFLQNKVQRPRGVGSFNQQQQQQQSNTNE |
| *PbTCP12* | MKSNQRQSLEESNELEHGGDSNNTTTTTSVSGPLVADPPATALTMKEELSDPVQHLDPQGSLSMGIVPVPVSTALKPARRSSKDRHTKVEGRGRRIRMPAVCAARIFQLTRELGHKSDGETIRWLLEHAEPAIIQATGSGTVPAIAISVGGTLKIPTTSPARPNGEITEIPNKRKKMRGS  TDEFIDAHDQNSLPSGLAPIAPMTYGGGGGGGGFVPMWQVGATGAAGPFFMFPNSGAIQNQPHLWAIPTADCTTPIFNFQPRPMSNFVSAFQPGVQVGGVSGGDGQLQASSGSISSGTAAGGGGSCSSSLGHSLDSASGTSAAAAAAAPPSSATQMLRDFSLEIYDKKELQFMGQAANAQTTYSES |
| *PbTCP17* | MDPKGSKQTQDIPSFLSLPPQPQSQPQQQQQQPQQPPQPNNNNMSDNKPAEIKDFQIVIADKDESGKKQLAPKRSSNKDRHTKVEGRGRRIRMPALCAARIFQLTRELGHKSDGETIQWLLQQAEPSIVAATGTGTMPASALAAAGGSVSQQGTSLSAGLHQKIDELGGSSGGRTGWAMVGGNLGRPHVAGVGGLWPPVSSFGFQSSSGPPSATTNLGTESSNYLQKIGFPGFDLPVSNMGPMSFTSILGGGNQQQQQQLPGLELGLSQDGHIGVLNSQALSQIYQQMGHARVHQQPPQHHQQQHHHHHQQPPSKDDSQGSGQ |
| *PbTCP23* | MIMEADNGIRCRPNFPLQFLDKTNQVDDDPEPAAACSTVADPGSDPNRTGSSIQEQQPSNKKPPPKRTSTKDRHTKVDGRGRRIRMPATCAARVFQLTRELGHKSDGETIEWLPQQAEPSVIAATGTGTIPSNFTSLNISLRSSGSSMSAPSHYLRTNASNYYFNSNNFGAAAPTSQLGTAEHSQRGRILFPGAGLMSSESSPPSSMLWNFNNSGNDNISNNTMLEAKQEIGDHHQAVTLDEASSMARKRIRPEQDLLSASTGSIPASQNTIPATFWMVANNNPSNSQGSIHHEHINSINPMWTIPTVGNAGNMYRGPVAGGGHGLHFMNFASPLAILPGQQLGTGSITTTQGAADSHLGMMAALNAYRPILGGVAMESPANGQHPHQGADEGHDRNTNAR |
| *PbTCP34* | MDPKGSKQTQDIPSFLSLPPQPQQQQPPPPQPNSNMNDDKPAEIKDFQIVIADKDESGKKQLAPKRSSNKDRHTKVEGRGRRIRMPALCAARIFQLTRELGHKSDGETIQWLLQQAEPSIGAATGTGTMSRRLWPPLGPGRFRRRLWPRQGALFRNRGLLYRLDCTKRSMNWGGPVEVGPAGQWWAGIWGDPMWQGWAGYGPLSVALGSSHHLVLHQPPQIWAVRVQITCKKLGFLALTCLSLTWVR |
| *PbTCP1* | MAPLLLFRWGERALLQTGLRLWELQRKLGHKSDGETIEWLLQQAEPSVIAATGTGTIPANFSTSNISLRSSGSSLSASPSKSAPHSFHGALALAHHPHYDEGFAHSGLMGFHQSPSPPPVTISTASQFTLPHNSLHFCSAHPPYHSTHPSPLDKPTEPPSLSPSANQRNPGAMTTVPGQLIWKIVKKNNSFLVKEFGRGNAGVRFSKEPNNLYNLHSYKHSGLANKKTVSIQAGGKDQSVVLATTATRSC |
| *PbTCP7* | MEGGDDHQLHHHHHYQQHQQQYHHRPNFPFQLLEKKELDHQEAASCSNSTSPYPSLAITTVDPTTNNNTSSPAATTTSALQASSAEPSKKPPPKRTSTKDRHTKVDGRGRRIRMPALCAARGFQLTHELGLLQQAEPAVIAATGTGTIPANFTSLNISLRSSGSSMSVPSQLRSSYYSPNFSLHQNQRRSLFQGIGLSSSDSSSTLLNFQTNSMHASMLQAKQELRDTVSLDLSEAASAGEGSMGGRKRRPPEQDLNQMGGGCGGGGGGYLLQSSTGAVPASHPHSQIPANFWMLANSNNQVMSGDPIWTFPASVNNSGLYRGTMPGGLHFMNFPAPVTLLPSQQQLGGSGGGGGSNDGNNMSDGQLNMLAGLNPYRHMSDTGVSDSQQASGSHSHHGGGDDRHDSTSHHS |
| *AtTCP1* | MSSSTNDYNDGNNNGVYPLSLYLSSLSGHQDIIHNPYNHQLKASPGHMVSAVPESLIDYMAFKSNNVVNQQGFEFPEVSKEIKKVVKKDRHSKIQTAQGIRDRRVRLSIGIARQFFDLQDMLGFDKASKTLDWLLKKSRKAIKEVVQAKNLNNDDEDFGNIGGDVEQEEEKEEDDNGDKSFVYGLSPGYGEEEVVCEATKAGIRKKKSELRNISSKGLAKARGKAKERTKEMMAYDNPETASDITQSEIMDPFKRSIVFNEGEDMTHLFYKEPIEEFDNQESILTNMTLPTKMGQSYNQNNGILMLVDQSSSSNYNTFLPQNLDYSYDQNPFHDQTLYVVTDKNFPKGKVWIQDSFVN |
| *AtTCP2* | MIGDLMKNNNNGDVVDNEVNNRLSRWHHNSSRIIRVSRASGGKDRHSKVLTSKGPRDRRVRLSVSTALQFYDLQDRLGYDQPSKAVEWLIKAAEDSISELPSLNNTHFPTDDENHQNQTLTTVAANSLSKSACSSNSDTSKNSSGLSLSRSELRDKARERARERTAKETKERDHNHTSFTDLLNSGSDPVNSNRQWMASAPSSSPMEYFSSGLILGSGQQTHFPISTNSHPFSSISDHHHHHPHHQHQEFSFVPDHLISPAESNGGAFNLDFNMSTPSGAGAAVSAASGGGFSGFNRGTLQSNSTNQHQSFLANLQRFPTSESGGGPQFLFGALPAENHHHNHQFQLYYENGCRNSSEHKGKGKN |
| *AtTCP3* | MAPDNDHFLDSPSPPLLEMRHHQSATENGGGCGEIVEVQGGHIVRSTGRKDRHSKVCTAKGPRDRRVRLSAPTAIQFYDVQDRLGFDRPSKAVDWLITKAKSAIDDLAQLPPWNPADTLRQHAAAAANAKPRKTKTLISPPPPQPEETEHHRIGEEEDNESSFLPASMDSDSIADTIKSFFPVASTQQSYHHQPPSRGNTQNQDLLRLSLQSFQNGPPFPNQTEPALFSGQSNNQLAFDSSTASWEQSHQSPEFGKIQRLVSWNNVGAAESAGSTGGFVFASPSSLHPVYSQSQLLSQRGPLQSINTPMIRAWFDPHHHHHHHQQSMTTDDLHHHHPYHIPPGIHQSAIPGIAFASSGEFSGFRIPARFQGEQEEHGGDNKPSSASSDSRH |
| *AtTCP4* | MSDDQFHHPPPPSSMRHRSTSDAADGGCGEIVEVQGGHIVRSTGRKDRHSKVCTAKGPRDRRVRLSAHTAIQFYDVQDRLGFDRPSKAVDWLIKKAKTSIDELAELPPWNPADAIRLAAANAKPRRTTAKTQISPSPPPPQQQQQQQQLQFGVGFNGGGAEHPSNNESSFLPPSMDSDSIADTIKSFFPVIGSSTEAPSNHNLMHNYHHQHPPDLLSR TNSQNQDLRLSLQSFPDGPPSLLHHQHHHHTSASASEPTLFYGQSNPLGFDTSSWEQQSSEFGRIQRLVAWNSGGGGGATDTGNGGGFLFAPPTPSTTSFQPVLGQSQQLYSQRGPLQSSYSPMIRAWFDPHHHHQSISTDDLNHHHHLPPPVHQSAIPGIGFA SGEFSSGFRIPARFQGQEEEQHDGLTHKPSSASSISRH |
| *AtTCP5* | MRSGECDEEEIQAKQERDQNQNHQVNLNHMLQQQQPSSVSSSRQWTSAFRNPRIVRVSRTFGGKDRHSKVCTVRGLRDRRIRLSVPTAIQLYDLQDRLGLSQPSKVIDWLLEAAKDDVDKLPPLQFPHGFNQMYPNLIFGNSGFGESPSSTTSTTFPGTNLGFLENWDLGGSSRTRARLTDTTTTQRESFDLDKGKWIKNDENSNQDHQGFNTNHQQQFPLTNPYNNTSAYYNLGHLQQSLDQSGNNVTVAISNVAANNNNNLNLHPPSSSAGDGSQLFFGPTPPAMSSLFPTYPSFLGASHHHHVVDGAGHLQLFSSNSNTASQQHMMPGNTSLIRPFHHLMSSNHDTDHHSSDNESDS |
| *AtTCP6* | MVMEPKKNQNLPSFLNPSRQNQDNDKKRKQTEVKGFDIVVGEKRKKKENEEEDQEIQILYEKEKKKPNKDRHLKVEGRGRRVRLPPLCAARIYQLTKELGHKSDGETLEWLLQHAEPSILSATVNGIKPTESVVSQPPLTADLMICHSVEEASRTQMEANGLWRNETGQTIGGFDLNYGIGFDFNGVPEIGFGDNQTPGLELRLSQVGVLNPQVFQQMGKEQFRVLHHHSHEDQQQSAEENGS |
| *AtTCP7*  *AtTCP8* | MSINNNNNNNNNNNDGLMISSNGALIEQQPSVVVKKPPAKDRHSKVDGRGRRIRMPIICAARVFQLTRELGHKSDGQTIEWLLRQAEPSIIAATGTGTTPASFSTASVSIRGATNSTSLDHKPTSLLGGTSPFILGKRVRADEDSNNSHNHSSVGKDETFTTTPAGFWAVPARPDFGQVWSFAGAPQEMFLQQQHHHQQPLFVHQQQQQQAAMGEASAARVGNYLPGHLNLLASLSGGSPGSDRREEDPR  MDLSDIRNNNNDTAAVATGGGARQLVDASLSIVPRSTPPEDSTLATTSSTATATTTKRSTKDRHTKVDGRGRRIRMPALCAARVFQLTRELGHKSDGETIEWLLQQAEPAIVAATGTGTIPANFSTLSVSLRSSGSTLSAPPSKSVPLYGALGLTHHQYDEQGGGGVFAAHTSPLLGFHHQLQHHQNQNQNQDPVETIPEGENFSRKRYRSVDLSKENDDRKQNENKSLKESETSGPTAAPMWAVAPPSRSGAGNTFWMLPVPTTAGNQMESSSNNNTAAGHRAPPMWPFVNSAGGGAGGGGGAATHFMAGTGFSFPMDQYRGSPLQLGSFLAQPQPTQNLGLSMPDSNLGMLAALNSAYSRGGNANANAEQANNAVEHQEKQQQSDHDDDSREENSNSSE |
| *AtTCP9* | MATIQKLEEVAGKDQTLRAVDLTIINGVRNVETSRPFQVNPTVSLEPKAEPVMPSFSMSLAPPSSTGPPLKRASTKDRHTKVEGRGRRIRMPATCAARIFQLTRELGHKSDGETIRWLLENAEPAIIAATGTGTVPAIAMSVNGTLKIPTTTNADSDMGENLMKKKRKRPSNSEYIDISDAVSASSGLAPIATTTTIQPPQALASSTVAQQLLPQGMYPM WAIPSNAMIPTVGAFFLIPQIAGPSNQPQLLAFPAAAASPSSYVAAVQQASTMARPPPLQVVPSSGFVSVSDVSGSNLSRATSVMAPSSSSGVTTGSSSSIATTTTHTLRDFSLEIYEKQELHQFMSTTTARSSNH |
| *AtTCP10* | MGLKGYSVGEGGGEIVEVQGGHIIRATGRKDRHSKVFTSKGPRDRRVRLSAHTAIQFYDVQDRLGYDRPSKAVDWLIKKAKTAIDKLELGETTTTTTRQEPVNTKPESPTLVFQRENNDQTQFVAANLDPEDAMKTFFPATTTTNGGGGTNINFQNYPHQDDNNMVSRTTTPPPNLSQDLGLSLHPFQGNNNTVVVPETNNFTTTHFDTFGRISGWNHHDLTMTSSSSSEHQQQEQEERSNGGFMVNHHPHHHHHQPSMMTLLNSQQQQVFLGGQQQQQQRGTLQSSLFPHSFRSWDHHQTTSDHHHHQNQASSMFASSSQYGSHGMMMMQGLSFPNTTRLLHGEEATQPNSSSSPPNSHL |
| *AtTCP11* | MIFQNVCRNESNFNAIASESRSQTQFGVSKSSSSGGGCISARTKDRHTKVNGRSRRVTMPALAAARIFQLTRELGHKTEGETIEWLLSQAEPSIIAATGYGTKLISNWVDVAADDSSSSSSMTSPQTQTQTPQSPSCRLDLCQPIGIQYPVNGYSHMPFTAMLLE PMTTTAESEVEIAEEEERRRRHH |
| *AtTCP12* | MFPSLDTNGYDLFDPFIPHQTTMFPSFITHIQSPNSHHHYSSPSFPFSSDFLESFDESFLINQFLLQQQDVAANVVESPWKFCKKLELKKKNEKCVDGSTSQEVQWRRTVKKRDRHSKICTAQGPRDRRMRLSLQIARKFFDLQDMLGFDKASKTIEWLFSKSKTSIKQLKERVAASEGGGKDEHLQVDEKEKDETLKLRVSKRRTKTMESSFKTKESRERARKRARERTMAKMKMRLFETSETISDPHQETREIKITNGVQLLEKENKEQEWSNTNDVHMVEYQMDSVSIIEKFLGLTSDSSSSSIFGDSEECYTSLSSVRGTISAAGNSNVLTKNPN |
| *AtTCP13* | MNIVSWKDANDEVAGGATTRREREVKEDQEETEVRATSGKTVIKKQPTSISSSSSSWMKSKDPRIVRVSRAFGGKDRHSKVCTLRGLRDRRVRLSVPTAIQLYDLQERLGVDQPSKAVDWLLDAAKEEIDELPPLPISPENFSIFNHHQSFLNLGQRPGQDPTQLGFKINGCVQKSTTTSREENDREKGENDVVYTNNHHVGSYGTYHNLEHHHHHH QHLSLQADYHSHQLHSLVPFPSQILVCPMTTSPTTTTIQSLFPSSSSAGSGTMETLDPRQM |
| *AtTCP14* | MQKPTSSILNVIMDGGDSVGGGGGDDHHRHLHHHHRPTFPFQLLGKHDPDDNHQQQPSPSSSSSLFSLHQHQQLSQSQPQSQSQKSQPQTTQKELLQTQEESAVVAAKKPPLKRASTKDRHTKVDGRGRRIRMPALCAARVFQLTRELGHKSDGETIEWLLQQAEPSVIAATGTGTIPANFTSLNISLRSSGSSMSLPSHFRSAASTFSPNNIFSPAMLQQQQQQQRGGGVGFHHPHLQGRAPTSSLFPGIDNFTPTTSFLNFHNPTKQEGDQDSEELNSEKKRRIQTTSDLHQQQQQHQHDQIGGYTLQSSNSGSTATAAAAQQIPGNFWMVAAAAAAGGGGGNNNQTGGLMTASIGTGGGGGEPVWTFPSINTAAAALYRSGVSGVPSGAVSSGLHFMNFAAPMAFLTGQQQLATTSNHEINEDSNNNEGG RSDGGGDHHNTQRHHHHQQQHHHNILSGLNQYGRQVSGDSQASGSLGGGDEEDQQD |
| *AtTCP15* | MDPDPDHNHRPNFPLQLLDSSTSSSSTSLAIISTTSEPNSEPKKPPPKRTSTKDRHTKVEGRGRRIRMPAMCAARVFQLTRELGHKSDGETIEWLLQQAEPAVIAATGTGTIPANFTSLNISLRSSRSSLSAAHLRTTPSSYYFHSPHQSMTHHLQHQHQVRPKNESHSSSSSSSQLLDHNQMGNYLVQSTAGSLPTSQSPATAPFWSSGDNTQNLWAFNINPHHSGVVAGDVYNPNSGGSGGGSGVHLMNFAAPIALFSGQPLASGYGGGGGGGGEHSHYGVLAALNAAYRPVAETGNHNNNQQNRDGDHHHNHQEDGSTSHHS |
| *AtTCP16* | MDSKNGINNSQKARRTPKDRHLKIGGRDRRIRIPPSVAPQLFRLTKELGFKTDGETVSWLLQNAEPAIFAATGHGVTTTSNEDIQPNRNFPSYTFNGDNISNNVFPCTVVNTGHRQMVFPVSTMTDHAPSTNYSTISDNYNSTFNGNATASDTTSAATTTATTTV |
| *AtTCP17* | MGIKKEDQKSSLSLLTQRWNNPRIVRVSRAFGGKDRHSKVCTVRGLRDRRIRLSVMTAIQVYDLQERLGLSQPSKVIDWLLEVAKNDVDLLPPLQFPPGFHQLNPNLTGLGESFPGVFDLGRTQREALDLEKRKWVNLDHVFDHIDHHNHFSNSIQSNKLYFPTITSSSSSYHYNLGHLQQSLLDQSGNVTVAFSNNYNNNNLNPPAAETMSSLFPTRYPSFLGGGQLQLFSSTSSQPDHIE |
| *AtTCP18* | MNNNIFSTTTTINDDYMLFPYNDHYSSQPLLPFSPSSSINDILIHSTSNTSNNHLDHHHQFQQPSPFSHFEFAPDCALLTSFHPENNGHDDNQTIPNDNHHPSLHFPLNNTIVEQPTEPSETINLIEDSQRISTSQDPKMKKAKKPSRTDRHSKIKTAKGTRDRRMRLSLDVAKELFGLQDMLGFDKASKTVEWLLTQAKPEIIKIATTLSHHGCFSSGDESHIRPVLGSMDTSSDLCELASMWTVDDRGSNTNTTETRGNKVDGRSMRGKRKRPEPRTPILKKLSKEERAKARERAKGRTMEKMMMKMKGRSQLVKVVEEDAHDHGEIIKNNNRSQVNRSSFEMTHCEDKIEELCKNDRFAVCNEFIMNKKDHISNESYDLVNYKPNSSFPVINHHRSQGAANSIEVPYLYLRVTFIIFMSFFPMVLSKAIHS |
| *AtTCP19* | MESNHEGNAIQVIDQVTTMTHLSDPNPKTKPGMMLMKQEDGYLQPVKTKPAPKRPTSKDRHTKVEGRGRRIRMPAGCAARVFQLTRELGHKSDGETIRWLLERAEPAIIEATGTGTVPAIAVSVNGTLKIPTSSPVLNDGGRDGDGDLIKKRRKRNCTSDFVDVNDSCHSSVTSGLAPITASNYGVNILNVNTQGFVPFWPMGMGTAFVTGGPDQMGQMWAIPTVATAPFLNVGARPVSSYVSNASDAEAEMETSGGGTTQPLRDFSLEIYDKRELQFLGGSGNSSPSSCHET |
| *AtTCP20* | MDPKNLNRHQVPNFLNPPPPPRNQGLVDDDAASAVVSDENRKPTTEIKDFQIVVSASDKEPNKKSQNQNQLGPKRSSNKDRHTKVEGRGRRIRMPALCAARIFQLTRELGHKSDGETIQWLLQQAEPSIIAATGSGTIPASALASSAATSNHHQGGSLTAGLMISHDLDGGSSSSGRPLNWGIGGGEGVSRSSLPTGLWPNVAGFGSGVPTTGLMSEGAGYRIGFPGFDFPGVGHMSFASILGGNHNQMPGLELGLSQEGNVGVLNPQSFTQIYQQMGQAQAQAQGRVLHHMHHNHEEHQQESGEKDDSQGSGR |
| *AtTCP21* | MADNDGAVSNGIIVEQTSNKGPLNAVKKPPSKDRHSKVDGRGRRIRMPIICAARVFQLTRELGHKSDGQTIEWLLRQAEPSIIAATGTGTTPASFSTASLSTSSPFTLGKRVVRAEEGESGGGGGGGLTVGHTMGTSLMGGGGSGGFWAVPARPDFGQVWSFATGAPPEMVFAQQQQPATLFVRHQQQQQASAAAAAAMGEASAARVGNYLPGHHLNLLASLSGGANGSGRREDDHEPR |
| *AtTCP22* | MNQNSSVAEATLQLNSGEKPSPGSIPFISSGQHGNISTSATSSTSTSSGSALAVVKSAVKKPTKDRHTKVDGRGRRIRMPAMCAARVFQLTRELGHKSDGETIEWLLQQAEPAIIASTGTGTIPANFSTLNASLRSGGGSTLFSQASKSSSSPLSFHSTGMSLYEDNNGTNGSSVDPSRKLLNSAANAAVFGFHHQMYPPIMSTERNPNTLVKPYREDYFKEPSSAAEPSESSQKASQFQEQELAQGRGTANVVPQPMWAVAPGTTNGGSAFWMLPMSGSGGREQMQQQPGHQMWAFNPGNYPVGTGRVVTAPMGSMMLGGQQLGLGVAEGNMAAAMRGSRGDGLAMTLDQHQHQLQHQEPNQSQASENGGDDKK |
| *AtTCP23* | MESHNNNQSNNNTTGSAHLVPSMGPISGSVSLTTTAPNSTTTTVTAAKTPAKRPSKDRHIKVDGRGRRIRMPAICAARVFQLTRELQHKSDGETIEWLLQQAEPAIIAATGTGTIPANISTLNISLRSSGSTLSAPLSKSFHMGRAAQNAAVFGFQQQLYHPHHITTDSSSSSLPKTFREEDLFKDPNFLDQEPGSRSPKPGSEAPDQDPGSTRSRTQNMIPPMWALAPTPASTNGGSAFWMLPVGGGGGPANVQDPSQHMWAFNPGHYPGRIGSVQLGSMLVGGQQLGLGVAENNNLGLFSGGGGDGGRVGLGMSLEQKPQHQVS DHATRDQNPTIDGSP |
| *AtTCP24* | MEVDEDIELQKHQEQQSRKLQRFSEDNTGLMRNWNNPSSRIIRVSRASGGKDRHSKVLTSKGLRDRRIRLSVATAIQFYDLQDRLGFDQPSKAVEWLINAASDSITDLPLLNTNFDHLDQNQNQTKSACSSGTSESSLLSLSRTEIRGKARERARERTAKDRDKDLQNAHSSFTQLLTGGFDQQPSNRNWTGGSDCFNPVQLQIPNSSSQEPMNHPFSFVPDYNFGISSSSSAINGGYSSRGTLQSNSQSLFLNNNNNITQRSSISSSSSSSSPMDSQSISFFMATPPPLDHHNHQLPETFDGRLYLYYGEGNRSSDDKAKERR |
| *PmTCP16* | MFPSSSSASNYQLPFPCDTNQQPQLLEKSSTNIIDQHGHENPNSISHDDHHIRQYYSHYSEDHQQQAPNNFLEHDGLLLSYLLSQQQLLVGSSSPNMNSATSHHVQVHDTTEISVVASNSNKKVMDRVDEGRTAPmATSKGCSSKKKSNTSNGESKNPKAPRKRSSGNKDRHSKIYTAQGPRDRRMRLSLQIARKFFDLQDMLGFDKASKTIEWLFTKSKTSIKELKQHLNISPLAAYSCSTNANATSTSTITAKMNSSTSENSEVASKIQETANGDVHSIGTLERDKKNRKLCVVARESRVEARARARERTREKMMRTRSGLDQYQNLRKQSPDHDQVPQNPNNDQFSGGHYLWSSNCPNDQLFEPGMMMNSMVMSINHHDEKVVGSTTIPSGANSEDYDFPSFPGNWGQINNSKNITGNNVLQVDPNPSNSSTMATAPPTSYPLEQKPSSVFMSTSSSMHEQNPSSIFVTTLIAEDQNPTTSSNFGTNSSIVLKSHFLGN |
| *PmTCP7* | MFSSCSSNINSNSVSPFQPYFPLSSSNYHPPPPPPPPPFPCVNQEACSGDIFLHHNIQGYPISGQFPLHNNALMAPPLPQSLTHLGVSSNTVPPSINADDHHHYHNYYGAINNDNIFPHFLHSSREDIVAPPMKKDRHSKIFTAQGLRDRRVRLSINVARQFFDLQDLLGFDKASKTLEWLLTKSRRAIKQLGTRNKHLTCSTSTGRSKSLTSSSECDDDDVDSDTNEVENVASKEKEVMLMMKKKMKESESANVYGTKDSRARARARARERTREKLMCTGSRPQMLNQLKLFNELDHHQSNNNCKTMSSSSAKANIGDHPENQELGSLLANQLAHHHEDDPSVHVIKRNKLKQYSSVYSNYNQQKYLNVVSAENTDQSSNSHIQFPNAFQNWDTNGAFPCPNIHCAITSINLSTGN |
| *PmTCP9* | MGLESLQEWVRSKAIHEMPGVGISSIQLGNKEEGKKQLAPKRSSNKDRHTKVEGRGRRIRMPmLCAARIFQLTRELGHKSDGETIQWLLQQAEPSIIAATGTGTIPmSALTAAGGSVSQQGTSLSAGLHQKIDELGGSSIGSGSRTSWAMVGGNLGRPHVATGLWPPVSSFGFQSSSGPSTTNLGSESSNYMQKIGFPGFDLPVSNMGPMSFTSILGGGSNQQLPGLELGLSQDGHIGVLNSQALSQIYQQMGHARVHQHQHQHQHQQPPmKDDSQGSGQ |
| *PmTCP2* | MEAENGIRCRPNFPLQFLDKNQDDDIQEAPGDPGSNQNRPGSSLTQEQQPNNGKKPPPKRTSTKDRHTKVDGRGRRIRMPmTCAARVFQLTRELGHKSDGETIEWLLQQAEPmVIAATGTGTIPmNFTSLNISLRSSGSSMSAPSHYLRPNSSNYFMNFNPNNLGAAAASQLIMDENSQRGRILFPGVGLLSSESSPSSSMLLNFNNAMLEAKRELRDQGGSLGEVSEGGEASTMGRKRIRPEQDLLSSSSSSSSSSQSSHHQMGISNSYMLQSASTGSIPmSQSTIPmTFWMVANNPSSGSGGGGHHVHEPPINPMWTIPSINNGINSNMYRGASVSSGGGHGLHFMNFAPPPMAAILPSHMGSTPHGGGGSIADSHLGVMAALNAYRPILGGVMAEPPGGNNGQHSHHGADEGHDRDSNAR |
| *PmTCP1* | MGSISIQAGLTSTHTPTTTSATVSAQPPmPSSSSLSSSSSSSPSSTSTSTAPPPHLVDASLAIATRSDSHPSTRPmPLLDSAKKNQIHQQQLTISPTSTTTATPPmPPmAAAATQVVKRSTKDRHTKVEGRGRRIRMPmTCAARVFQLTRELSHKSDGETIEWLLQQAEPmIIAATGTGTIPmNFSSLNISLRSSGSTLSAPPSKSASHSFPGALALAHHPHYDEGFPHSGLLGFHQQQHHHHHHQQHQSPLMTADQIAEALPSGGGDSGGGDSTDSYMRKRFREDLFKDDNQGRGEGGGGSSGGGGSPSGNKAFKSSGGLQLQKQQQGSEGEAGPSGLLRPTSNILPPTAMWAVAPmPNSGAAGSTFWMLPVTAGAGGPSMPTGTSGAGPSEAAQMWTFPSAPmSHGSTLQAPLHFMPRFNLPSSLEFQQAGSRGSPLQLGSMLMQQQPSQHLGLGVADSNLGMLAALNASYSRGGLNMNSEHHQQNHPLDHHHQQQQHQPQTTDSGDEAPNSSQ |
| *PmTCP3* | MAENKPmQIKDLQILKANKDDENKKHLAPKRSSNKDRHKKVDGRGRRIRMPmLCAARVFQLTRELGHKSDGETIQWLLQQAEPSIIAATGSGTVPmSALAAAGSSVSEQGSSVSSVSSGLHTRMEGLIRPSVGSEGSANWAFHMGRSNVASGVWPLPFLSGVRSGFDQNSGQATVNFGSENLNIMQHKFGFHGFDVPGMNLGSMGFPTILSGSNQQVPGLELGLSQDGHIGVVSSQALTQFHQHKGQRPGGVASFNQQQQHQQPSDTNE |
| *PmTCP18* | MFPSNSNNNLNNTGNELPVSYPHVDQSFFHSRPFLHEITTLNPNSLHPNLNSKQEEEQRQQEGPHHHPLSFFYFPSPLEDDDVLLFQQHHHYDHQHDHVHDMPLHDSQQAPPLTTMREAVAANNTLADDHRQTTTTSTTLNIKMVDWDSNKNHGEMMNMDQPQIPRRRSCKRDRHSKINTARGLRDRRMRLSLEVARKFFWLQDALHFDKASKTVEWLLIQATPEIKKLVGDCKHMMSSTKSTSPmTSESCEVISGIDEAATNTNIHINIDGGNDGDDKLIQSCEIQPSAKERKVARRQLSRKTAFHPLSKASREKARARAREKAREKQRTHQRVVDVDDQSKKQRGDQENLSRLGSWSPFETGEESAGTQSHNNNNISINSLEGLVHHEIEEPMSSCQVGDHPDLVVDHGTTHDPLVIMGKWSPPSIFSSLQQNIGISQEVTSNNKF |
| *PmTCP10* | MTMTEKRELERDQATTSVDLRINGGEESDSEEPmGPSQALMLAPKDERDVAMPVAVHAPKRASTKDRHTKVEGRGRRIRMPmTCAARIFQLTRELGHKSDGETIRWLLEHAEPmIIAATGTGTVPmIAMSVNGALKIPTSAPDPRPGEDPPDKKKRKRNSNSEYVDLNDGVSASAGLAPLTTERHHQQPPTAAVQAVVPQGVLPMWAIPSNGVVPGAFFMVPSASTQPHIFTFPTTVAAAPFINISARPISSFVGPSSASATTHMAASTAPQTLRDFSLEIYDKKELQFMSGSSNH |
| *PmTCP5* | MEVEEIQAQACKFPRIGNGSSRATNPmADDEDQDPSCLDFKRDTTADAGNRLRGWHHSRIIRVSRASGGKDRHSKVWTSKGLRDRRVRLSVTTAIQFYDLQDRLGYDQPSKAVEWLIKAAAEAIAELPSLNNSFPDTPKQLSDEKRASCEHGFDSAEVELEGHGHGDPNYHHHQNQNQTQNQSQYLSLSKSACSSNSETSKGSGLSLSRSEIRVNRVKARERARERAAKDKEKESNESASHIAHHPQQNSSNLNNSISQSASFTELLTGGIGTNSSNNNNSPTAAAHQQQNHGGGGGEPILFHKAAAAGGGGPMDYFSSGLLGLSSSTRTHHSSGFPGQIQLGMNSIPQTMSVVSPFSVSGDHHHNHNPELQHFSFVPDHLIPVTTSSQPGNGGDYNLNFSISSSGGLAGFNRGTLQSNSSSSPSLLPHHLQRFSPIDGSSNVPFFIGAAAAPTMENHHHHHHHHQQHQQQFPmGFDRRLQHPYGDGSRHSDHKGKAKN |
| *PmTCP12* | MTSYLEDQDDDGGTSDLSTSTGDPEDNNNNGNGVVSTQPNFDETTAFQQLKEEPIDSDPPPQAHSIGMVPVAMQMPMSVAVPVSNPTRRASTKDRHTKVEGRGRRIRMPmTCAARIFQLTRELGHKSDGETVRWLLEHAEEAIIEATGTGTVPmIAVSVDAVSQSSGLAPVGPmAPQGLVPVWAVGGAGLMVPmNAFWMGPVGSGGGPSGPQPQIWALSPTVTPVFNVAGATRPVSSFVANNGGGVEVRAPSPmLSNSAVSTSTVGPRAAKRSSTTMAPSVSSSSNNSNGSGSGASKAQMLRDFSLEIYDKQELQFMGRPVGSPTTHQHQTQ |
| *PmTCP17* | MGMKSTGGGGGGEIIQVQGGHIVRSTGRKDRHSKVYTAKGPRDRRVRLSAHTAIQFYDVQDRLGYDRPSKAVDWLIKKAKSSIDKLAELPPWHPITGVAANNAEPDQSNPNEMVIAGGAEETESSGYNFHQLQRQMGENNNNQANNVSSFNIPPSLDSDTIADTMKSFFPTSSAANSSINFQSYPmDHDLISRTTNLNPCQDLGLSNPKGTPTTTTIRFRPFSPRGREDQQWDLTLVIREWWHGAIRTEEAMVDLYSTHTHKHCHSRHMLMLRGQRTQQQQQPMIHHSSIFGTRFASDGGLPVFCIPTRIDAEEADNGGVSDRPSSTSSPNSTHH |
| *PmTCP11* | MEPNQRQSLEESNELEQQSNSSSNDNTTSASDPSVADPPEKIFPLTAPTMKEELTDTVQELDEGSLPMGLIQVPVPTSSEKQVVAAKRSSKDRHTKVEGRGRRIRMPmTCAARIFQLTRELGHKSDGETIRWLLEQAEPmIMQATGTGTIPmIAVSVGGTLKIPTTSPmRPNGEITEIPRKRRKRGSNSEFVDVHEQSSVSSGLAPMSYGGGGGGGGAHGLVPMWQVGATGAAGPFFMFPNNGAVNPNQPQLWAVPmADAATPIFNFQARPISNFLSAFQPGVHVVGGDVQLQASSGSISSGATSGSGGSCSSSLGPSLGSASGTRANKNTISTGSGTGASAAAAAASATTTQMLRDFSLEIYDKRELQFMGANSQTPYSKP |
| *PmTCP4* | MSHLQDILRLQMLQQRGSKNEDQDQPEDVQEEEQQSQKRLIGQYQHVQEPPNYGPLNGKMLNTHIAKSSRKSCYSMSSSSSHLASEQAKINNARYGKIVKVHGGHIVRSTARKERHSKVYTSKGPRDRRFRLSAPTAIQFYDVQDRLGYDRPSKAIDWLIEKAKAAIEALSESELPGKEYDCTNINNSAQQTEQDIGEESMRQFQHHQRSYGGEPEKLNNVNSFKEPVLDHYQLSSMNYAEEALNSGSSLTDSKMEVAWFQSLLAWNYNAGDGGEGCPFNSSHVYLQ |
| *PmTCP19* | MGSEMALAPLRNHEDPTLIPTAVLSSAALETASSRQHSQQSIQSLQPKTHNQLSQTRKSASSRDRHTKVNGRGRRVRMPmMCAARIFQLTRELGHRSDGETIEWLLRHAESSIVAATGTGTLPmEPISTSAPmVSSQVPSLACRAHPLSSLNGGGQFMYPLVSSAHQAQPNQPHQQPSIRLDLCQPmGLDYTEYRQHMPFTSLLLHPmENEEEEEEDGQQEEALCLGDG |
| *PmTCP6* | MGDTHHHHHPQATTSSRLGIRPSSGLSADIVEVVRGSHIVRSTGRKDRHSKVCTAKGPRDRRVRLAAHTAIQFYDVQDRLGYDRPSKAVDWLIKKAKAAIDELDELPSWNPHSVSTTTASTAVTAMEAQNPTTTGFHCFAAVDAIGSANRRATMVGSGVSEQIVQNQNPLTNSTLLPPSLDSDAIADTIKSFFPMGASAGAAEAPSSTIQFQNYPPDLLSRTSSQSQDLRLSLHSFQDPILLQHQQAQAQHHQAQTHQNEQTLFSGTQQQNPLGFDGWTEHHHQQQQAEMNRFQRMVAWNSAGGGDTGNGGGSSSSAGFVFNSLLPTQQSTSSLQPSLFGQGHFFSSQRGPLQSSNSPSVRAWMMDQQNQQSISHDHHHHHQISPTIHHHNQSSSSISNMGFASGGGFPGFHIPmRIHGEEEHDGISDKPSSASSNSRH |
| *PmTCP8* | MIKSHNEADLQEAAGNSSRDDQANKFSTKANDLSRPSTPWLRLKDPRIVRVSRAFGGKDRHSKVCTVKGLRDRRVRLSVPTAIQLYDLQERLGLNQPSKVVDWLLDAAKHEIDELPPLPLPPSGNFGLNHPSLVLTSSHGVQTHAHAQLSHDNGEGPSGGIAPmRSHFWSTNSDAIWRGKSKEIARDTTNEEEEENQKDISTGSDQKEEGTVDGNSSSNNFLTRISTNHPFFPGLVNNAMPYAYHNWDHNQPSNFPLSQLGSHGFPSQTADLHNFINVVSLPSTLSLSTTQSHFPSHNAAAAAEIDPRQFNHMHMLSSSSTSQNLLPNSLSPTLYPNSQTLRAPHLSMMTKLVRSSNNTTGSDHHQPNTDQESPSR |
| *PmTCP14* | MISNSREKGFQAKQEGHNNTNNDGNSSNFNKESSSSTTTSRQWSGFRNPRIVRVSRTFGGKDRHSKVSTVRGLRDRRIRLSVPTAIQLYDLQDRLGLSQPSKVIDWLLDVTEDDIDKLPPLQVPHGFAHQFHQQMLNPHHSHDQGHQSNNSLAAAPFFDVNSSFMEADHQAHQVVHDHQRSSTSTNVGDRKGKSIKTHDEQDDDDQNHHQDGNIGGGQLLAQKLFPQGNHPSSIPGLLNNAMAYNYYHNYSEPSSLSLSQFGGHGFPPVPQIDHHSHMMSNALSFSTSMPSGSQLFFCPSTATPSLFGPYPPYITNPVVERGTNTSEPRSQANHFQFLSSSNSPNLLPNALMSSLQSLKSYPTSVNPKQLHSNSQDNNGSQPNKDHN |
| *PmTCP13* | MEGGDQDHQLHHHHHHHYQQQQHHHRPNFPFQLLEKKELDQEAASCSNSTSPYPSLAITTVDPmTAITTTTTTTSTLQASAEPSKKPPPKRTSTKDRHTKVDGRGRRIRMPmLCAARVFQLTRELGHNSGSSMSVPSQLRSSYYSPNFSVHQNQRRSLFQGIGLSSSDSSSTLLNFQTNSMHASMLQAKQELRDTVSLDLSETASGEGSMGGRKRRPPEQDLNQMGGGGGGGGGGYLLQSSTGAVPmSHHHSQIPmNFWMVANSNNQVMSGDPIWTFPmSVNNSGLYRGTMPGGLHFMNFPmPMTLLPSQQQLGGSGGGGGGNDGDNMSDGQLNMLAGLNPYRHMSSTGVSESQQASGSHSHHGGDDRHDSTSHHS |
| *PmTCP15* | MEINQTLPTNTLAITDPPENPSLQTQPPQQEEQQQLERRRYIDKHSTVNGRHRRVRIPVTCCPGIFRLTQELGHRSDGDTIQWLLSQVRPELVLPPQPNNRTRRLPPDPVPQPNCRYPGAAEDWLDHKAVARLPSVTVRATVVQASTVFFDTPmTLDKAERLVAGAAAYGSQLVVFPEAFVGGYPRGLMFDSATATLSPEEKQAFEKYYASAIDVPGPEVDRLAKIASKYKVHLVMGVVERVGFYLCSTVLFFDSFGQCLGKHPKLLPLASESPVWCSGPKLPVSVYDTEIGRIGGLVCWDNRMPDLRTQLYGKGIEIYCAPTAEAREIWRSSMTHIALEGGCFVLSANQFCRRKDYPLPLECVSGDSNDATSLDIICAGGSVIVSPSGTILAGPNYQGESLISADLDLVEIARAKLEFGGVGLGHNAGPNAVGWRRTSIPNPDLFAATVKTEVSDHANVLYA |
| *PaTCP5* | MFSSCSSNINSTSVSPFQPYFPLSSSNYHPPPPPPPPLPCVNQEPCSGDIFLHHNIQGYP  ISGQFPLHNNALMAPPLPQSLTHLGVSSNTVPPSINADDHHPYHSHYGAINNGNIFPHFLHSSREDIVAPPMKKDRHSKIFTAQGLRDRRVRLSINVARQFFDLQDLLGFDKASKTLEWLLTKSRKAIKQLGTRNKHLTCSTSTGRSKSLTSSSECDDDDVDSDTNEVENVASKEKEVMLMMKKKMKESESANVYGMKDSRAKARARARERTREKLMCTTRSGPQMLNQLKLFNELDHHQSNNNCETMSSSSAKANIGDHPENQELGSLLANQLAHHHEEDPSVHVIKRNKLKQYSSGYSNYNQQKYLNVVSAESTDQSSNSHIQFPNAFQNWDTNGAFPCPNIHCAITSINLSTGN |
| *PaTCP13* | LNSFNGSAHHQVVLKVQASPSLDQHPSPPTQPQQAKGGDIMFPSNSNNNLNNTGNELPVSYPHVDQSFFHSRPFLHEVTTLIPNSLHPNLNSKQEEEQQQQQQGPHHHLSPFFYIPSTFEDDDVLLFQQHHHYDHQHDHVHDMPLHDTQQAPPITTMRETVAANNTLADDHRQTTTTSTTLNIKMVDWDSNKNHGEMMNMDQPQIPRRRSCKRDRHSKINTARGLRDRRMRLSLEVARKFFWLQDALHFDKASKTVEWLLIQATPEIKKLVGDCKHMMSSTKSTSPATSESCEVISGIDEAATNTNIHINIDGGNAGDDKLIRSCEIQPSAKERKVARRQLSRKTAFHPLSKASREKARARAREKAREKQRTHQRVVDVDDQSKKQSGDQENMSRLSSWSPFETGEESAGTQSHNNNNININSLEGLVHHEIEEPMSSCQPGDHPDLVVDHGTTHDPLVIMGKWSPPSIFSSLQQNIGISQEHHQFADFQFFGKPWEVYNNTHNLF |
| *PaTCP1* | MFPSSSSASNYQLPFPCDTNQQPQLHEKSSTNIIDQHGHENPNSISHDDHHIRQYYSHYSEDHQQQAPNNFLEHDGLLLSYLLSQQQLLVGSSSPNMNSATSHHVQAHDTTEISVVASNSNKKVMDRVDEDRTSPAATSKGCSSKKKSNTSNGESKNAKAPRKRSSGNKDRHSKIYTAQGPRDRRMRLSLQIARKFFDLQDMLGFDKASKTIEWLFTKSKTSIKELKQHLNISPLAAYSCSTNANATSTSTITAKMNSSTSENSEVASKIQETAIGDVHSIGTLEREKKNRKLCVVARESRVEARARARERTREKKMMRTRSGLDQYQNLRKQSPDHDQVPQNPNNDQFSGGHYLWSSNCPNDQLFEPGMMMNSMVMSINHHDEKVVGSTTIPSGANSEDYDFPSFPGNWGQFNSKNITGNNVLQVDPNPSNSSTMATAPPTPYPLEQKPSSVFMSTSSSMQEQKPSSIFVTTLIAEDQNPTTSSNFGTNSSIVLQSQFLGN |
| *PaTCP2* | MIMEAENGIRSRPNFPLQFLDKNQDDDIQEAPGDPGSNLNRPGSSLTQEQQPNNGKKPPPKRTSTKDRHTKVDGRGRRIRMPATCAARVFQLTRELGHKSDGETIEWLLQQAEPAVIAATGTGTIPANFTSLNISLRSSGSSMSAPSHYLRPNSSNYFMNFNPNNLGAAAASQLIMDENSQRGRILFPGVGLLSSESSPSSSMLLNFNNAMLEAKRELRDQGGSLGDVSEGGEASAMGRKRIRPEQDLLSSSSSSSSSSQSSHHQMGISNSYMLQSASTGSIPASQSTIPATFWMVANNPSSGSGGGGHHVHEPPINPMWTIPSINNGINSNMYRGASVSSGGGHGLHFMNFAPPPMAAILPSHMGSTPHGGGGSIADSHLGVMAALNAYRPILGGVMAEPPGGNNGQHSHHGADEGHDRDSNAR |
| *PaTCP11* | MSNVGCGADHISCGTQQRIQSISSWHFKFEILARQQVQPRAQCSTGYSFFKGFKSEPSKKPPPKRTSTKDRHTKVDGRGRRIRMPALCAARVFQLTRELGHKSDGETIEWLLQQAEPAVIAATGTGTIPANFTSLNISLRSSGSSMSVPSQLRSSYYSPNFSVHQNQRRSLFQGIGLSSSDNSSTLLNFQTNSMHASMLQAKQELRDTVSLDLSETASGEGSMGGRKRRPPEQDLNQMGGGGGGGGGGYLLQSSTGAVPASHHHSQIPANFWMVANSNNQVMSGDPIWTFPASVNNSGLYRGTMSGGLHFMNFPAPMTLLPSQQQLGGSGGGGGGNDGDNMSDGQLNMLAGLNPYRHMSSTGVSESQQASGSHSHHGGDDRHDSTSHHS |
| *PaTCP10* | MEVEEIQAQACKFPRIGNGSSRATNPAADDEDQDPSCLDLKRAATADAGNRLRGWHHSRIIRVSRASGGKDRHSKVWTSKGLRDRRVRLSVTTAIQFYDLQDRLGYDQPSKAVEWLIKAAADAIAELPSLNNSFPDTPKQLSDEKRASCEHGFDSAEVELEGHGHGDPNYHQNQNQTQNHSQHLSLSKSACSSNSETSKGSGLSLSRSEIRVKARERARERTAKDKEKESNESASHIAHHHQQNSSNMNNSISQSASFTELLTGGIGTNSSNNNNSPTAAAHQQQNHGGGGEPILFHKAAAAGGGPMDYFSSGLLGLSSSTRTHHTSGFSSQIHLGINSIPQTMSVVSPFSVSGDHHHNHNPELQHFSFVPDHLIPVTTSSQPGNGGDYNLNFSISSSGGLAGFNRGTLQSNSSSSPSLLPHHLQRFSPIDGSSNVPFFIGAAAAPTMENHHHHHHHHQQHQQQFPAGFDRRLQHPYGDGSRHSDHKGKAKN |
| *PaTCP9* | LDPQRNHHHHYLHLPLHQNQNPIINFTHQQQGQEEEEEDHQNHQAFNFQQHPYLQQADQQQQHHQHFFGGLLLDTRSTEAAELQAQQQEAPKKRNFFTSSSSTLGEQSIEYARSKMGDTHHHHHPQATTSSRLGIRPSSGLSADIVEVVRGSHIVRSTGRKDRHSKVCTAKGPRDRRVRLAAHTAIQFYDVQDRLGYDRPSKAVDWLIKKAKAAIDELDELPSWNPHSVSTTTPSTAVTAMETQNPTTTGFHCFAAVDAIGSANRRATMVGSGVSEQIVQNQNPLTNSTLLPPSLDSDAIADTIKSFFPMGASAGAAEAPSSTLQFQNYPPDLLSRTSSQSQDLRLSLHSFQDPILLQHQQAQAQHHQAQTHQNEQTLFSGTQQQNPLGFDGWTEHHHQQQQAEMNRFQRMVAWNSAGGGDTGGGFSGFHIPARIHGEEEHDGISDKPSSASSNSRH |
| *PaTCP7* | MSHLQDILRLQMLQQRGSKNEEQENEDQDQPEDVQEEEQQSQKRLIGQYQHVQEPPNYGTLNGKMLNTDMAKSSRKSCYSMSSSSSHLASEQAEINNARYGKIVKVHGGHIVRSTARKERHSKVYTSKGPRDRRFRLSAPTAIQFYDVQDRLGYDRPSKAIDWLIEKAKAAIEALSESELPGKEYDCTNINNSAQQTEQDIEEESMHQFQHHQRSYGGEPEKLNNVNSYKEPVLDHHQLSSMNYAEEALNSASSLSDSKMEVAWFQRLMAWNYNAADGGEGCPFNSSHLYLQ |
| *PaTCP8* | MGMKSAGGGAGGEIIQVQGGHIVRSTGRKDRHSKVYTAKGPRDRRVRLSAHTAIQFYDVQDRLGYDRPSKAVDWLIKKAKSSIDRLAELPPWHPITGVAANNADPNQSNPNEMVIAGGTEETESCGYNFHQLQRQMSENNNNNNQANNVSSFNIPPSLDSDTIADTMKSFFPTSSAATSSINFQSYPPDHDLISRTTNLNPSQDLGLSLHSFQDQGLNIHHTHSQQSQGDTNHNNDQIQTLFAAGAGGSTVGFDTSYQRMVAWSNQNRGVDGGFVFNSHTQALPQQAYAHAQGGTLQSSFSPSVSARAWNDSSIFGTQQRTQQQQPMIHHSSIFGTRFASDGGLPVFCIPTRIDAEEGDNGGVSDRPSSTSSPNSTHH |
| *PaTCP14* | MIKSPNEADLQEAAGNSSRDDQANKFSTKANDLSRPSTPWLRLKDPRIVRVSRAFGGKDRHSKVCTVKGLRDRRVRLSVPTAIQLYDLQERLGLNQPSKVVDWLLDAAKHEIDELPPLPLPPSGNFGLNHPSLVLTSSHGVQTHAHAQLSHDNGEGPSGGIAPARSHFWSTNSDAIWRGKSKEIARDTTNEEEEENQKDISTGSDQKEEGTVDGNSSSNNFLTRISTNHPFFPGLVNNAMPYAYHNWDHNQPSNFTLSQLGSHGFPSQTADLHNFINVVSLPSTLSLSTTQSYFPSHNAAAAAEIDPRQFNHMHMLSSSSTSQNLLPNSLSPTLYPNSQTLRAPHLSMMTKLVRSSNNTTGIDHHQPNTDQESPSR |
| *PaTCP12* | MISNSREKGFQAKQEGHNNTNTDGNSSSFNKASSSSTTTSRQWSGFRNPRIVRVSRTFGGKDRHSKVSTVRGLRDRRIRLSVPTAIQLYDLQDRLGLSQPSKVIDWLLDVTEDDIDKLPPLQVPHGFGHQFHQQMLNPHHSHDQGHHSNNSLAAAPFFDVNSSFMKEADHQAHQVVHDHQRSSTTSNVGDQKGKSIKTPDEQDDDDQNHHQDGNIGGGQLLAQKLFPLGNHPSSIPGLLNNAMAYNYYHNYSEPSSLSLSQFGGHGFAPVPQIDHHSHMMSNTLSFSTSMPSGSQLFFCPSTATPSLFGPYPPYITNPVVESGTTTSEPRSQANHFQFLSSSNSPNFLPNALMPSLQSLKSYPTSVNPKQLHSNSQDNNGSQPNKDHN |
| *PaTCP6* | MDPKGSKQTQEIPSFLSLPQPQQQQQQQQQQQQQQQQQPNMSENKPAEIKDFQIVIADKEEGKKQLAPKRSSNKDRHTKVEGRGRRIRMPALCAARIFQLTRELGHKSDGETIQWLLQQAEPSIIAATGTGTIPASALTAAGGSVSQQGTSLSAGLHQKIDELGGSSIGSGSRTSWAMVGGNMGRPHVATGLWPPVSSFGFQSSSGPSTTNLGSESSNYLQKIGFPGFDLPVSNMGPMSFTSILGGGSNQQLPGLELGLSQDGHIGVLNSQALSQIYQQMGHARVHQHQHQHQHQQPPAKDDSQGSGQ |
| *PaTCP3* | MAENKPAQIKDLQILKANKDDENKKHLAPKRSSNKDRHKKVDGRGRRIRMPALCAARVFQLTRELGHKSDGETIQWLLQQAEPSIIAATGSGTVPASALAAAGSSVSEQGSSVSSVSSGLHTRMEGLIRPSVGSEGSANWAFHMGRSNVASGVWPLPFLSGVRSGFDQNSGQATVNFGSENLNIMQHKFGFHGFDVPGMNLGSMGFPTILSGSNQQVPGLELGLSQDGHIGVVSSQALTQFHQNKGQRPGSVASFNQQQQQQPSDTNE |
| *PaTCP4* | LTRELGHKSDGETIRWLLEHAEPAIIAATGTGTVPAIAMSVNGALKIPTSAPDPRPGEDPPDKKKRKRNSNSEYVDLNDGVSASAGLAPLTTERHHQQPPTAAVQAVVPQGVLPMWAIPSNGVVPGAFFMVPSASTQPHIFTFPTTVAAAPFINISARPISSFVGPSSASATTHMAASTAPQTLRDFSLEIYDKKELQFMSGSSNH |
| *MdTCP1* | MKSNQRQSLQESNELERGGDSNNTSTSGSSPSVANTPEQNFSATVLMMKEELAGPVQQLDQQGSLSMGIDRHTKVEGRGRRVRMPATCAARIFQLTRELGHKSDGETIRWLLEHSEPAIIQATGSGTIPAIAVSVGGALKIPTTSPARPNGEGTEIPKKRKKMRGSLGEFADVHDQSSGLAPIAPMIYGGGGGGLVPMWQIGATGAAGPFFMFPNSGTIQNQPQLWAIPTADSTAPIFNFQPRPLSNFVSAFPAGVQVGSVGGGDGQLQASSCSIPGGAATGGGGSCSSSLGHSLESASGTSAAASAAAAPRSATTQMLKDFSLEVYDKRELQLMGQAANSQTPRSES |
| *MdTCP2* | MGVERYYQLAKXPATIGYRKLENGLPEGGEEQQKYSGGXLWSSTTSRECDPRIVRASRAFGGKDRHSKVCTIRGFRDRRVRLSVPTAIQLYDLQERLGLNQPSKVVDWLLDDDHNQVRLLPGKEHEVQRYKHECSYQPSSSSFWLLGFSKATKEGSLYVSTLKMIEEEDE |
| *MdTCP3* | MVEKRRRTKHLKIPQFSGHRKNQSGELARKKVRVGARGPVPSWARGGTCHNDPPASAQTRRPKPDRDPHSHFDFFHHPNSSIXLAFPTIRPLTXGPAQAPKTGNPLEHQGRHTKVNGWGRRVLMLAMCAAQIFQLTQELGHRFDGETIEWLIRHAEPSIIASTDFGTLSTEPISTTTMFVSSQMPFIPC |
| *MdTCP4* | MNEHDIQIPKIVTKKIQKEXDGXKSMQLTLLQDPXQRIRTEALLSSSPHTSLQSQIIDDPPASAQTRRPKPDRDPHSHFDFFHHPNSSIXLAFPTIRPLTXGPAQAPKTGNPLEHQGRHTKVNGWGRRVLMLAMCAAQIFQLTQELGHRFDGETIEWLIRHAEPSIIASTDFGTLSTEPISTTTMFVSSQMPFIPC |
| *MdTCP5* | MLRQRESKNKEEQNEDYQHVHYLLPTSKSAFKQPPVDNARYEHIVKVHGGHIVRSLARKDRHSKVYTSKGPRDRRFRLSAHTAIQFYDVQDRLGYDRPGKAIDWLIKKSKAAIEALSKSEQPCQEYNDCTNTNVFGQQTEQEIGEQSMHQFWNHPESNGELETMSNVSPMNNVNNLKEPVFDPHQLSSLNYAEEALDSASSLSDSKFKEMGWFQSLVAWNYNASDGGEICPYNSSHVSLH |
| *MdTCP6* | MLRQRESKNKEEQNEDYQHVHYLLPTSKSAFKQPPVDNARYEHIVKVHGGHIVRSLARKDRHSKVYTSKGPRDRRFRLSAHTAIQFYDVQDRLGYDRPGKAIDWLIKKSKAAIEALSKSEQPCQEYNDCTNTNVFGQQTEQEIGEQSMHQFWNHPESNGELETMSNVSPMNNVNNLKEPVFDPHQLSSLNYAEEALDSASSLSDSKFKEMGWFQSLVAWNYNASDGGEICPYNSSHVSLH |
| *MdTCP7* | MFLRTPEGKAIFERWELVSTVGCRKLENGLTEGGEEQQKYSGGRLWSSTTSRECVAREPNKISSNPNLSRSSTPWPRLKDPRIVRASRAFGGKDRHSKVCTIRGLRDRRVRLSVPTKIQLYDLQERLGLNQPIKVVDWLLDDDHNQESCPSIKNILLLDSEGKRVAVKYYSEEDWPTXAAKESFEKAVFTKTQKTNARTEGNVDKKEALENLDLILLCLDEIVDGGIVLETDSNVIASKVASHSIDAGAPLSEQSKQASSNGKTALFSLPVKKHTKLTSSFFANPRLFTSFTSKAVSEFDGRHEPNLYPRNEAVFRPSKPFLSKSNTPRTPEIWGGSDGGGREGRRRLEIWGEDQQSGEGIGKSSSNKKIFGXFFSLPFSYSV |
| *MdTCP8* | MXXEEIQASNKFPRIGNGSSRDHKPSPEDEDNQNPSCLDLKRAAAATATADAGNRLRGWHHSRIIRVSRASGGKDRHSKVWTSKGLRDRRVRLSVTTGIQFYDLQDRLGYDQPSKAVDWLIKAAADAIAELPSLNNSSFPDTPKQLSDEKRASCERGGFDSAEIEFDQNYHQNQSQPGNQSQHLSLSKSACSSTSETSKGSGLSLSRSEIRVNRSKARERARERAAKDKEKESIESSYQQSINNNISQQNASFTELLTAGIGTTHNNNSPTASAQHHQQNHGGGGEPILFHKAAAPMDYFSPGLLGLSSSARTHHSSGFSEQIHLGMNSIPQTMSVVSPFNVSGEHHHGHHSSELQHFSFVPDLIPVTTSSQPGSGVDYNLNFSISSSGGLAGFNRGTLQSNSSSSPSLMPHHLQRFSPIDGTSNVPFFIGAAAAAASPTMENHHHHHHQQHQQHQQQFPGGFDRRLQQLYGDGTRHSDHKGKAKN |
| *MdTCP9* | MXXEEIQASNKFPRIGNGSSRDHKPSPEDEDNQNPSCLDLKRAAAATATADAGNRLRGWHHSRIIRVSRASGGKDRHSKVWTSKGLRDRRVRLSVTTXIQFYDXQDRLGYDQPSKAVXWLIKAAADAIAELPSLNNSSFPDTPKQLSDEKRASCERGGFDSAEIEFDQNYHQNQSQPGNQSQHLSLSKSACSSTSETSKGSGLSLSRSEIRVNRSKARERARERAAKDKEKESIESSYQQSINNNISQQNASFTELLTAGIGTTHNNNSPTASAQHHQQNHGGGGEPILFHKAAAPMDYFSPGLLGLSSSARTHHSSGFSEQIHLGMNSIPQTMSVVSPFNVSGEHHHGHHSSELQHFSFVPDLIPVTTSSQPGSGVDYNLNFSISSSGGLAGFNRGTLQSNSSSSPSLMPHHLQRFSPIDGTSNVPFFIGAAAAAASPTMENHHHHHHQQHQQHQQQFPGGFDRRLQQLYGDGTRHSDHKGKAKN |
| *MdTCP10* | MXXEEIQASNKFPRIGNGSSRDHKPSPEDEDNQNPSCLDLKRAAAATATADAGNRLRGWHHSRIIRVSRASGGKDRHSKVWTSKGLRDRRVRLSVTTXIQFYDXQDRLGYDQPSKAVXWLIKAAADAIAELPSLNNSSFPDTPKQLSDEKRASCERGGFDSAEIEFDQNYHQNQSQPGNQSQHLSLSKSACSSTSETSKGSGLSLSRSEIRVNRSKARERARERAAKDKEKESIESSYQQSINNNISQQNASFTELLTAGIGTTHNNNSPTASAQHHQQNHGGGGEPILFHKAAAPMDYFSPGLLGLSSSARTHHSSGFSEQIHLGMNSIPQTMSVVSPFNVSGEHHHGHHSSELQHFSFVPDLIPVTTSSQPGSGVDYNLNFSISSSGGLAGFNRGTLQSNSSSSPSLMPHHLQRFSPIDGTSNVPFFIGAAAAAASPTMENHHHHHHQQHQQHQQQFPGGFDRRLQQLYGDGTRHSDHKGKAKN |
| *MdTCP11* | MEVEEIQASNKFPRIGNGSSRDHKPSPEDDDNQDPSCLEVKRDAATTAAADAGNRLRGWHHSRIIRVSRASGGKDRHSKVWTSKGLRDRRVRLSVTTAIELYDLQDRLNYDQPSKAIEWLIKAAADAIAELPSLNNSSFPDTPKQLSDEKRASCERGGFDSAEIEFDQNYNQNQNQTGNQSQHLSLSKSACSSTSETSKGSGLSLSRSEVRVNRSKARERARERAAKDKDKESIESAYPHNISQQNSSFTELLTAGIGTAHNNNDSRTASAHHHQQNHGCGGGEPILFHKAPAPMDYFSPGLLGLSSSARTHHSAGFSGQIHLGMNSIPQTMSVVSPFNVSGEHHHGHHSSELQHFSFVPDLIPVTTSSEPGNGGDYNLNFSISSSGGLAGFNRGTLQSNSSSSPSLMPHHLQRFSPIDGSSNVPFFIGAAAAAAAPTMENHHHHHHHHHQQHQQQFPGGFDRRLQQLYGDGTRHADHKGKAKN |
| *MdTCP12* | MTSYLEDQDDDGGASDLSTSTGDLGDNDHTNRDGVAYPIGMVPVAMPMPMSVPSTVSTLIRRSSTKDRHTKVEGRGRRIRVPATCAARIFQLTXELGNESDGETVRWLLEHAEQAIIEATGTGTVPAIAVSVGGSLKIPTTASTSVEDNSSPPSSTKKQKRPSNSEFLDVENDAVSQSSGLAPVGPSVPQGLVPVWAVGGPGLMVPANALWIGPIWALPPTMTPVFNMAGAARPISSFVANRGGGGLGVLDVRAPSPPLSNSAANTSTVGPRAAMRSSTTMAPSVSSSSNNSNGTGSGATTKTQMLRDFSLEIYDKXELQFMGRPVGPENHHQTQ |
| *MdTCP13* | MEGGDDHQLHHHHHHHHYQQHQQQYHHRPNFPFQLLEKKELDHQEAASCSNSTSPYPSLAITTVDPTANNNTSSTPAATTTSALQASSAEPSKKPPPKRTSTKDRHTKVDGRGRRIRMPALCAARVFQLTRELGHKSDGETIEWLLQQAEPAVIAATGTGTIPANFTSLNISLRSSGSSMSVPSQLRSSYYSPNFSLHQNQRRXLFQGIGLSSSDSSSTLLNFQTNPMHASMLQAKQELRDTVSLDLSEAASAGEGSMGGRKRRPPEQDLNQMGGGGGGGGGGYLLQSSTGAVPASHPHSQIPANFWMLANSNNQVMSGDPIWTFPASVNNSGLYRGTMPGGLHFMNFPAPVTLLPSQQQLGGSGGGGGSNDGNNMSDGQLNMLAGLNPYRHMSDTGVSD  SQQASGSHSHHGGGDDRHDSTSHHS |
| *MdTCP14* | MEGGDDHQLHHHHHHHHYQQHQQQYHHRPNFPFQLLEKKELDHQEAASCSNSTSPYPSLAITTVDPTANNNTSSTPAATTTSALQASSAEPSKKPPPKRTSTKDRHTKVDGRGRRIRMPALCAARVFQLTRELGHKSDGETIEWLLQQAEPAVIAATGTGTIPANFTSLNISLRSSGSSMSVPSQLRSSYYSPNFSLHQNQRRSLFQGIGLSSSDSSSTLLNFQTNPMHASMLQAKQELRDTVSLDLSEAASAGEGSMGGRKRRPPEQDLNQMGGGGGGGGGGYLLQSSTGAVPASHPHSQIPANFWMLANSNNQVMSGDPIWTFPASVNNSGLYRGTMPGGLHFMNFPAPVTLLPSQQQLGGSGGGGGSNDGNNMSDGQLNMLAGLNPYRHMSDTGVSDSQQASGSHSHHGGGDDRHDSTSHHS |
| *MdTCP15* | MITNSRDKGFQAKQEGHNNNNNNDGSNNSSFHKASSSSTTTSRQWSGFRNPRIVRVSRTFGGKDRHSKVSTVRGLRDRRIRLSVPTAIQLYDLQDRLGLSQPSKVIDWLLDITEQDIDKLPPLQVPHGFGHQFHQPMLNPHQVSNSLVAPFFDVNSTFMEADQVDHQEVRDQAKGKSIKTNDGQDDQNRHDHEGNVGQLLAQKLFPIGSSSIPGMLNNAMAYNYYHNYSEPSTLSLAQFGSHGFPQVPQLDQHHSHMMSTNALSFSTPMPSVSQLFFRPPTATPMLFGSYPPYITNPIVEGTSTATDQPRQANHFQFLSSPSNSQNFLANNALMPPLHSVSSSLKSFPSLVHPKQQLHLNSQNNNGSQPNKDVP |
| *MdTCP16* | MVFLLCCLSQPLYLLSMTIDGNLRHCRQQLSFSSAQLAVNTVTHGCLSQSCPIISLTTSAGKVKNKCTEISFNGNGFIIKSFSKPTKQAKSLRGRYNMFPSHNNNLNELVPVSYPHVDQSIFHSWPSYHDNSTLTPNSLTILNPNPNSRQQEGGEENLHQQHHHLPFSLLYFPSPFEDDDVLLFDQQHHHQEPDHIELSIHESQEPLYFMKEAAAAAAADDNTVVGNDHQKTTTTSVNRKMVDWDSNKKGHRMNMDDQPQIPRKRTSKRDRHSKINTARGPRDRRMRLSLEVALKFFGLQDALGFDKPSKTVEWLLIQSEPAIKKLSRDHHRQFNYKHMVRCAKSTSPATSESCEVLSGVDEAPTNVNISINGKVRSRGIKPSAKERKFVHRQSRKSAFHPLAKASREKARARARERTREKMQRSKKPSNDQANSSRLSSWNPFETEEESPAHNNNMNSTTNDQPNSRAVLRDYPDEVQEPLSSPQAGNIQDMVVDHGTTHDAMVVLGKWSPPPVFTRLQQNTGISQEVTSTTKFQLL |
| *MdTCP17* | MHQIPPSATDGKGVAAGVKNLEPRSKFMSPSSSELGSELLSPSSSEASSFPHRPSRCRSPDWKHLNPCLPPSTHGSFVVRDRKKTEEVGEKYYQLAKNPTTIGYWKLENGLPEDGEEHQKYSGGRLWSLTTSRECVAREANKISSNPNLSRSLTPWPRLKDPRIVRASRAFGGKDRHSKVCTIRGLRDWRMRLSVPTAIQLYDHQERLGLNQPSKVVDWLLDDDHNQVRLLPG |
| *MdTCP18* | MKSNQRQSLEESNELEHGGDSNNTTTSASGPLVADPPATALTMKEELTDPVQHLDPQGSLSMGIVPVPVSTALKPARRSSKDRHTKVEGRGRRIRMPAVCAARIFQLTRELGHKSDGETIRWLLEHAEPAIIQATGSGTVPAIAISVGGTLKIPTTSPARPNGEITEIPNKRKKMRGSTGEFIDAHDQNSLPSGLAPIAPMTYGGGGGGLVPMWQVGATGAAGPFFMFPNSGAIQNQPQLWAIPTADSTAPIFNFQPRPMSDFVSAFQPGVQVGGVSGGDGQLQASSGSMSSGTAAGGGGTCSSSLGHSLDSASGTSAAAAADAPPSSATQMLRDFSLEIYDKKELQFMGQAANAQTTYSES |
| *MdTCP19* | MGVERYYQLAKNPATIGCRKLENGLLEGGEEQQKYSGGWLWSSMTSRECVAKEANKISSNPNLSRSSTPWPRLKDPRIVCASRAFGGKDMHSKVCTIRGLRDRHVRLSVPTAIQLYDLQERLGLNQPSKVVDWLFDDDHNQVQLLPG |
| *MdTCP20* | MGVERYYQLAKNPATIGCRKLENGLLEGGEEQQKYSGGWLWSSMTSRECVAKEANKISSNPNLSRSSTPWPRLKDPRIVCASRAFGGKDMHSKVCTIRGLRDRHVRLSVPTAIQLYDLQERLGLNQPSKVVDWLFDDDHNQVQLLPG |
| *MdTCP21* | MLTCAVSLLPXLPSTGTNWTHPPTPSPSDIXNLPFAPEGINVSVKKADSYHSFHQTPRLEGGGRRAEXGGRRTEXGEIFLRRXRGRRRTPLSVGSISIQAGLTTTSHSTPPPPPTSAPSSSSFSSSSSTSSPSSTSTSAAPPPHLVDASLAIATRSDPHPPTRPASLLDSAKNNQTQQQLTISPATTSPPSAXAAPATQVAKRSTKDRHTKVEGRGRRIRMPATCAARVFQLTRELGHKSDGETIEWLLQQAEHAIIATTGTGTIPANFSTLNISLRSSGSTLSAPPSKSAPHSFPGALALAHHPXYDEGFAHSGLLGFHQQHQQQQHQQHHHQPHQSPLMTADQIAEALPSGGGDSAGGGGGESTENYMRKRFREDLFKDDNQGQGESGSGDGGGSPSRNKAFKGASSGGLQXPKQQQQGAEVGSSGVLRPSSILPATAMWAVAPAPTSGAGNTFWMLPVTAGPGVPSMAXATFGAGPSEAAQMWTFPSSTASHGSTLQAPMHFMPRFNLPSSLEFQQAAAGGRGSPLQLGSMLMQQPPSQHLGLGVSDSNLGMLAALNAYSRGGLNMNSDQHQQNHHHPLDHHHQEQQQHQPQTXDSGDEAPNSSQ |
| *MdTCP22* | MDPKGSKQTQDIPSFLSLPPQSQPQPEQQQQPQQQPQPNNNMSDNKPAEIKDFQIVIADKDESGKKQLAPKRSSNKDRHTKVEGRGRRIRMPALCAARIFQLTRELGHKSDGETIQWLLQQAEPSIVATTGTGTIPASALAAAGGSVSQQGTSLSAGLHQKIDELGGSSGGRTSWAMVGGNLGRPHVAGVGGLWPPVSSFGFQSSSGPPSATTNLGTESSNYLQKIGFPGFDLPVSNXGPMSFTSILGGGNQQQQQQLPGLELGLSQDGHIGVLNSQALSQIYQQMGHARVHQQPPQHHHQQHHHQQQQPPSKDDSQGSGQ |
| *MdTCP23* | MGXERYYQLAKNLAIIGCRKLENGLPEGGEEHQKHSGGRLWSSTTSRECVAREANKISSNPNLSRSSTLWPRLKDPRIVRASRAFGGKDRHSKVCTIRGLRDRRVRLSVPTAIQLYDLQERLGLNQPSKVVDWLLDDDHNQVRLLPG |
| *MdTCP24* | MIKSLISEADFKEAGAGSASRDEQANKTSSNPNLSRSSTPWPRLKDPRIVRVSRAFGGKDRHSKVCTIRGLRDRRVRLSVPTAIQLYDLQDKLGLNQPSKVVDWLLDAAKHEIDELPPLPMPPPGTFGLNHPSLVLTSSHDDQTNAHPQLSHNHRGEGPSSGIDRSNVWPTNLDAXWRAKSKEIARDTRNEEEEEKRKDNLGISDDQKQAGNNIDGNSSNXFLTRGSNTNPPFFPGLLNSSTNMPYAYQNWDHPHQTSSFPLSQLGSHGFQSQXTDLHNFLNVLSLPSTLSLSTTQSYFPSHNAAATGEIDPRQFNHLQLLNSSSSTSTSQNLLPNSLSTPAPYPSSQTLRAPHLSMVTKLVHSSNNTGSGHQSNNDQGPLPR |
| *MdTCP25* | MGMKGCGGEIVQVQGGHIVRSTGRKDRHSKVYTAKGPRDRRVRLSAHTAIQFYDVQDRLGYDRPSKAVDWLIKKAKSSIDKLAELPPWHPITTSNHAAXADDPFRSSNPNPNQNPNEMVIAAAEQQSESSAGYNFXFELHRQRQSDNESNFNIPPSLDSDNIADTMKSFFPTNTSSNAAAASSVVDFQSYAPDPHLISTTTQDLGLSLHSFQDQSLNIHHNHTHTQQSSQTLFAAAMGIGLDSSSYQRMVAWSNENRGLDGGFVFNSHSHSYAQPQPHHHGGTLQSSFTPSVSARAWQQQQQHSSIFGTRFGASNGSSPVFCIQGEEAENGVVSDRTASTSSPNSTRQL |
| *MdTCP26* | MTSYLEDQDENGGASDLSTSTGDPGDNDHTNGDGVVSMQPNCEETXEFHSLKEEPIDSDPQQPPQGHPIGMLPDTMQMPVPMSVQLPVSAPTRRSSTKDRHTKVEGRGRRIRIPATCAARIFQLTRELGHKSDGETVRWLLEHAEQAIIEATGTGTVPAIAVSVGGTLKIPTTSSTSVEDNSSPTSATKKRKRPSNSEFVDVRKDAVSQSSGLAPVGPAAPQGLVPVWAVGGAGLIVPANAFWMGPVGSGAGSPGPQPQIWALSPTMTPVFNMAGAAGPISSFVVNSGGGAGGVDVRAPSPALSNSAASTSTVGARAAKRSSTTMAPPSVSSSSNNRVAEAEVEVTLRVSGQPFFAGRPYAHVVKNKFNPXNTPTLL |
| *MdTCP27* | MELKTLNGRTFWEKQKQKKQKQLQGSEGRWQHKHQEENHKGLTLDPQRXHHHHYLHQNPILNFNHLVQEEEEEETQQQTQQNHHQAFNFPYLHQDPHTQQHFFGETRQLQLQEQQQTQQRAPKKRSFTSSSSSTLVEQSIEYARSKMGDSHHHHLNNHQATTSSRLGIRPPSSGGGVSTDIVEXVRGSHIVRATGRKDRHSKVCTAKGPRDRRVRLAAHTAIQFYDVQDRLGYDRPSKAVDWLIKKAKAAIDELEELPSWNPHSISTTAAVPAMETQNPSATGIHCFAAVDAIGSANRRTMVGSGVSEQQFAQNPNSNSTFLPPSLDSDAIADTIKSFFPMGASAAAAAAESPSSTIQFHQNYPPDLLSKASSQSQDLRLSLQSFQDPILLHHHDAQNQHHHAQTHQNEQTLFSGSQNQLGFDGSPGSWAEHHHNQQQQEMNRFQRMVAWDSGGNGGPSSAGGFIFNSLLPTQQGSTSPLQQQQPSLFGQSQFFSQRGPLQSSNSPSVRAWMVDQQNQQSISHDHHHQISQSIHHQQSISGMGFASGGGFSGFHIPARIHGEEEHDGLKLSSHLCLPQRDYKSYLLVSNSYMARRRFFGRFVQWRAPWEEEI |
| *MdTCP28* | MSNSEAPNNELTNGGGGAGAMVEQTSQRPSSNGVLAVKKPPSKDRHSKVDGRGRRIRMPIICAARVFQLTRELGHKSDGQTIEWLLRQAEPSIIAATGTGTTPASFSTVSVSLRGGNTSSSAPSTTSSDHKPQLLXPTPFILGKRVRGSLDDPENNHSHNHNHDASAAKDPSDNHANNDGAVSVVGHSMASMLGPNAGGPGGFWAPHFGQVWSFAATPPPELMAQSALSHQQQQHQHHQQHSLFLQQQPMGEASAARVGNYLPGHLNLLASLSGGHGNSGRREDDQR |
| *MdTCP29* | MATSKSAFKQQQVDNTRYGKIVKVNGGHIVRSMARKDRRSKVYTLKGPRDRRFRLSPHTAIQLYDVQDRLGYDRLSKAIDWLIEKSKEAIEALSGSEQPCQDYYDCTNSNVFGQQTEQEIGEQSMHQLWNHPESNRVLETMSNVGPMNNVNNFKEPVFDQLSSLNYAEEALDPTSSLSDSKVTEMGWFQSLVAWNYNAGDGGEICPYNSSHRLRGLQKLSCCCSIHKMYPSSSSSSPQKPIGLRPSTTGLTRYGSSIGSLPNSAVDSVIGTAADFDFLSLRSQPLIGHYFFGDGHDDSFSLTSSKSTFKVNSSNGGYREASTTKPLL |
| *MdTCP30* | MHQTNERLIMSILFGTYIRLLGEPEAAEAEAASGLRGEVAAQASGDPSSKEKPLAKRGLFVKENHKGLTLDPQRNHDHHYLHQNPILNFTHLVQREEETQQNHHQAFNFPYFQQDPQTQHQHQHFFGETQQLQLQEQQQAPKKRSFTSSSSTLGEQGIEHARSKMGDSHHLHHHQATTSSRLGIRPPSSGAGVVSTDIVEVVRGSHIVRATGRKDRHSKVCTAKGPRDRRVRLAAHTAIQFYDVQDRLGYDRPSKAVDWLIKKAKAAIDELDELPSWNPHSTSTTASVPGMETHNPTTTGIHCFAGVDAIGSANQRTTMVGSGVSEQQIVQNPNSNSTFLPPSLDSDAIADTIKSFFPTGASAIAAAAEAPSSTIQFHQNYPPDLLSRTSSQSQDLRLSLQSFQDPILLHQHHAQTHQNEQSLFSGSQNPLGFDGSSAAWAEHHHNQQQQEMNRFQRMVAWNSGGGDSGDNGGGSSSSGGFTFNSLLPTQQSGTSSLQQQQPSLFGQSQFFFQRGPLQSSNSPSIRAWMMDQQNQQSISHDHHHQISQSIHHQPSFSGMGFTSGGGFSGFHIPARIHGEEEHDGISDKPSSASSNSRH |
| *MdTCP31* | MASEMALQPLHKLEDRKPTVLPTAVLTSATIQTAPSLQHSQLSDLQLQAQPKPQKQVSRRNTRDRHTKVNGRGRRVRMPAMCAARIFQLTRELGHRSDGETIEWLLRHAEPSIIAATGSGTLPAEPISTTSPSVSSQMPSVPCRVQPLKSLRSSGQFLYPAAAQAHQAMQLQLDLCQPMGVDYNAGYRHMPFTSLLLQTGESEVEEGQPEEEVVGVGIGDDE |
| *MdTCP32* | MDMIQEEQQHERDRDQDEEASAADLGIDGGXESDPEDPADPSRALALPPKQEQDVPMTVAVHAPRRASTKDRHTKVEGRGRRIRMPATCAARIFQLTRELGHKSDGDTIQWLLEHAEPAIIAATGTGTVPAIAISVNGSLKIPTDVRPGDDPPDQKKRKRNSNSEYVDLNDGVSVSAGLAPLTTEGRRQPPAPQPPTTAVQAVAPQGMVPMWAIPSNTVVPGAFFMVPSXTQPHIFTFPTTLPAAPFINISARPISSFVGPXSAAPATATSSGGGTQTLRDFSLEIFDKKELQFMSGSSNH |
| *MdTCP33* | MFPSSSTTSNTQXPFLCDDNHTNGIIDDFHQNPNSISSHGGYHQYYSQYYYSDDQQQQQAPDFLEHDGMLLSYLLSQQQLLIDSSSTTNVATHSNHXRAHSSAEIXIVDSNSNKTMELINRADEITVIPAADSQIKKNNVKMNGEGGGEKKAKFARKRASGKKDRHSKIYTAQGPRDRRMRLSLQIARKFFDLQDMLGFDKASKTIEWLFTKSKTAIKDLKQHLLVSPKEDYSSTNGGATAKVNSFESTTGDVASKIMEPNSSAAKGDISIGLAREKKYRKLSVVARESRVEARARARERTREKMMRIRGFDQDHQSTKQSHKHQEQQNPNELFETAGMMNSMMMSMNRPNDGQKIVGRTSSNCGGAERLFLNFDFSRHAEASGANSEDCDFPGNWGAINNTKNIITGNVLQVEQNPTSYPKQQNPSSIFGTGTQEQNPNSIFLTTLAKAQDQNSTTSSNFAINTNISTLQPLFTGNMFPSSSTTSNTQXPFLCDDNHTNGIIDDFHQNPNSISSHGGYHQYYSQYYYSDDQQQQQAPDFLEHDGMLLSYLLSQQQLLIDSSSTTNVATHSNHXRAHSSAEIXIVDSNSNKTMELINRADEITVIPAADSQIKKNNVKMNGEGGGEKKAKFARKRASGKKDRHSKIYTAQGPRDRRMRLSLQIARKFFDLQDMLGFDKASKTIEWLFTKSKTAIKDLKQHLLVSPKEDYSSTNGGATAKVNSFESTTGDVASKIMEPNSSAAKGDISIGLAREKKYRKLSVVARESRVEARARARERTREKMMRIRGFDQDHQSTKQSHKHQEQQNPNELFETAGMMNSMMMSMNRPNDGQKIVGRTSSNCGGAERLFLNFDFSRHAEASGANSEDCDFPGNWGAINNTKNIITGNVLQVEQNPTSYPKQQNPSSIFGTGTQEQNPNSIFLTTLAKAQDQNSTTSSNFAINTNISTLQPLFTGN |
| *MdTCP34* | MEADNGIRCRPNFPLQFLDKTNQVDDDPEPAAACSTVADPGSDPNRTGSSIQEEQPSNKKPPPKRTSTKDRHTKVDGRGRRIRMPATCAARVFQLTRELGHKSDGETIEWLLQQAEPSVIAATGTGTIPANFTSLNISLRSSGSSMSAPSHYLRTNASNYYFNSNNFGAAAPTAQLGTAEHSQRGRILFPGAGLMSSESSPPSSMLWNFNNSGNNNLSNNTMLEAKQELGDHHQAVTLDEASSMARKRIRPEQDLLSASTGSIPASQNTIPATFWMVANNNPSSSQGSIHHEHINSINPMWTIPTVGNAGNMYRGPVAGGGHGLHFMNFASPLAILPGQQLGTGSITTTQGAADGHLGMLAALNAYRPILGGVAMESPVNGQHPHQGADEGHDRNTNAR |
| *MdTCP35* | MAENNKHPAQIKDLQIMIANKEKDESKKQLAPKRSSTKDRHKKVDGRGRRIRMPALCAARVFQLTRELGHKSDGETIQWLLQQAEPSIVAATGSGTMPASALAAAAESSVSEQGNSVSSGLMSFGHMGRSNAATGIWPFVSGIRSSFDHNSGQQTLNFGGENLNFMHKFGFQGFDAPGNLGSVSFPTTLSGSNQQVPGLELGLSQDGHIGLVNSQALSLTQFLQNKVQRPRGVGSFSQQQQQQQQSNTNE |
| *MdTCP36* | MGWLSMRLFKDVMKWYHAVIKRGITHYTPESVVALSLARVVPVSIFFRLFREPTRFVLTPLCKSNIPLHHCGKEKTVEEIYYQLAKNPTTIGCRKLENGLPEGGEEQQKYSGGRLWSSTTSRECVAKEANKISSNPNLSRSSTPWPRLKDPRIVHASRAFGGKDRHGKVCTIRGLRDRRVRLSVPTAIQLYDLQERLGLNQPSKVVDWLLDDDHNQVRLLLG |
| *MdTCP37* | MYTGGAFXADSELKKIDELLLLDLALHKCQKVKSSXKXPLGEDRRNPSRERQGCTKLFVFPARPTNPAPAFLWRNPTVAAVRTSANLAASSKETQFHFFNFGGKEHRICKIKNEGFSPSPVHHQATTSSRMGIRSPSSSGGVVNTDIVEVVQGSHIVRATGWKDRHSKVCKAKGLRDRCVWLAAHTAIQFYDVQYRLDYDRPSKVVDWLIKKAKAAIDELPSWNPHSTSTTTAAIVVRPKPVLFS |
| *MdTCP38* | MRSWKEVMIINSTTTTTTINNTNNINNNITTPSPPSTPPPPTTRPLLPQPQPPQLSKLLPPSPPKKPPPKRTSTKDRHTKVDGRGRRIRMPALCAARVFQLTRELGHKSDGETIEWLLQQAEPAVIAATGTGTIPANFTSLNISLRSSGSSMSVPSQLRSSYYSPNFSLQQNQRRSLFQGIGLSSSDSSSTLLNFQTNTMHASMLQAKQELRDTVSLDLSEAASAGEGSLGGRKRRPPEQDLNHMGGGGGGGGGGGGYLLQSSTGVVPASHPHSQIPANFWMLANSNNQVMSGDPIWTFPASVNNSGLYRGTMPGGLHFM  NFPAPVTVLPNQQQLSGSGGGGGNDGDNMSDGQLNMLAGLNPYRHMSGTGVSDSQQASGSHSHHGGGDDRHDSTSHHS |
| *MdTCP39* | MITNSRDKGFQAKQEGHNNNDGNNNSSFNKASSSSTTTSRQWSGFSNPRIVRVSRAFGGKDRHSKVSTVRGLRDRRIRLSVPTAIQLYDLQDRLRLSQPSKVIDWLLGVTEQDIDKLPPLQVPHGFGHHQPMLNPHQANNSLIAPFFDVNSTFMEADQVDEEVRDQXKGKSIKTNDEQDDQNRHDHEGNVSGQHLAQKLFPXGNHPSSIPGLLNNAMAFNYYHNYSEPSTLSLAQFGSHGFPQVPQTDQHRSHMMSTNALSFSTPMASGSQLFFCPPTATPTLFGSYPPYMTNPIVEGTSTATDQPRQASHFQFLSSSSNSQNFLANNALMPYLHSISSSLKCFPSLVDPKQQLHLDSQNNNASQPNKDVP |
| *MdTCP40* | MFPSDNTSNVNELIPVSYPHVDQPFFHSRPFDHDITTVTPNFFTNSNPNPNSGQQQEGGEENLHQQHHHPPLSLLYFPSPFEDDDVLLFQQHHHQEPDHIGISLHESQVPPYFMKEAAAAATATAAAXAAAAXAAATAADDNTGTVVGVDHQKTTTTSVNIKMVDWDSNKNGHHVYMDDQPQIPRRRTSKRDRHSKINTARGPRDRRMRLSLEVARKFFGLQDVLEFDKASKTVEWLLIQSEPEIKKLSRDHHRKFNYKNMVRCAKTTSPATSESCEMLSGVDEAPTNINISNGGNDDNDKVRSSGIKPSAKERKIVHRQSRKGAFHPLAKASREKARARARERTREKMQRSKKPSNDQAKLSRLNSWNPFESGEESSAYNNNMNNTTNDQPDSMVALRPYPDGVEEPLSSSLAGDIQDMVVDHGTTHDAMXVMGKWSPSSVFTPLQQNTGISQEHQQFADFQFFDKPWDVYNNTHKLF |
| *MdTCP41* | MVANRWLRPEARDLSNVRCHKGRRRELRVEAEARRDAGGAAFGSEKGAQKTKKKNRRKQLSCLAGEGCFAQRENGKERQTDWQIAKDLTGAERARREEEKQSWLLSLAHLGFESTHSSPRTIETRQIGRHIPLSFHALPPSAPAAPATQVAKRSTKDRHTKVEGRGRRIRMAATCAARVFKLTREFGHKSDGETIEWLLQQARHAIIATTGTGTIPANFSTLNISLRSSGSTLSAPPSKSAPHSFPXALALAHHPHYDEGFAH |
| *MdTCP42* | MAAVEIINHHPKTKIIKTHHAQTSRRSPPPPPQLMRETVSMGCTTPRIICVSRASGIKDRHSKVWTSKRLRDRRVHLSVTTAIQFYDLQDRLGLQFENSCEVGVFSKLTNAYCLVSIGGSENFYSTFEAELADVIPVVKTSIGGTRIIGRLCAGNKNGLLLPHTTTDQELQHLRNSLPDNVVVQRIEERLSALGNCIACNDHVALTHTDLDRETEEMIADVLGVEVFRQTIAGNILVGSYCSFTNRGGLVHPHTSVEDLDELSTLLQVPLVAGTVNRGSEVIAAGMTVNDWTAFCGLDTTATELSVIESVFKLREAQPSAIVDEMRKSLIDSYV |
| *MdTCP43* | MASDLLQEGEEEEGHPSMGSISIQAGLTTTSHSTPPPPTTSAPSSASFSSSSSTSSPSSTSTSTAPPQLVDASLAIATRSDPHPPTRPGSLLDSAKNNQIQQQLTISPATTPPPAAAATTTPVAKRSTKDRHTKVEGRGRRIRMPATCAARVFQLTRELGHKSDGETIEWLLQQAEPSIIATTGTGTIPANFSTLNISLRSSGSSLSAPPSKSAPHSFHGALALTHRPHYDEGFAHSGLLGFHQQPQQQQQQHPQHHHQPHQSPLMTADEIAEALPSGGGDSGGGGGGESTENYMRKRFREDLFKDDNQERGESGSGGGVGSPSRNKAFKGGSSCGLQLPKQQQQGEELGSYSSGVLRPSNILPATAMWAVAPAPTSGAGSTFWMLPVTAGSAVPSMATGTSGAGPSEATQMWTFPSATASHVSTLQAPLHFMPRFNLPGSLEFQQAGSRGSPLQLGSMLMQQQPSQHLGLGVSESNLGMLAALNAYSRGGLNMNSDHQQNHQHPLDHHQQQHHQHQTTDSGDEGPNSSQ |
| *MdTCP44* | MASDLLQEGGGGGGHPSMGSISIQAGLTTTSHSTPPPPTTSAPSSASFSSSSSTSSPSSTSTSTAPPQLXYSSLAIATRSYPHPPTIPGSLLDSAXXNQIQQQLTISPATTPPPAAAATTTPVAKRSTKDRHTKVEGRGRRIRMPATCAARVFQLTRELGHKSDGETIEWLLQQAEPSIIATTGTGTIPANFSTLNISLRSSGSSLSAPPSKSAPHSFHGALALTHRPHYDEGFAHSGLLGFHQQPQQQQQQHPQHHHQPHQSPLMTADEIAEALPSGGGDSGGGGGGESTENYMRKRFREDLFKDDNQERGESGSGGGVXSPSRNKAFKGGSSCGLQLPKQQQQGEELGSYSSGVLRPSNILPATAMWAVAPAPTSGAGSTFWMLPVTAGSAVPSMATGTSGAGPSEATQMWTFPSATASHVSTLQAPLHFMPRFNLPGSLEFQQAGSRGSPLQLGSMLMQQQPSQHLGLGVSESNLGMLAALNAYSRGGLNMNSDHQQNHQHPLDHHQQQHHQHQTTDSGDEGPNSSQ |
| *MdTCP45* | MVSSSSTTSNFQLPFLCDDNQTNAIIDDFHQNPNSILSHGGHHQYYSQYYYSEDQQQQAPEFLEHDGMLLSYLLSQQQLLLGXXSTTTNVATDYNPTRAHESTEISIAASNSNKAMEVIHRDXEDGRNELTINTAAASKIKINNVKINGGGGGGEKKANVARKRASGKKDRHSKIYTAQGPRDRRMRLSVQIARKFFDLQDTLGFDKASKTIEWLFTKSKSAIKDLKQHLLVSPKEDYNSTNGGATAKVNSSENTTGEVASRIMEPSSSDANGDISVGFGREKRNRKLCVVARESRVEARARARERTREKMMRIRGFDQDHQHLTKQSPKHQEQQNPNELFETAGMMNSTMMSTNCRNXGQKIVGRTSSGCGGAQRSLLNFDFWRHVSEASGANSEDCGFSRELGSNQLHQEYYYR |
| *MdTCP46* | MIMEADNGIRCRPNFPLQFIDKTNQVDDDPEPAAACSTVADPGSDLXRTGSSIQEQQPSNKKPPAKRTSTKDRHTKVDGRGRRIRMPATCAARVFQLTRELGHKSDGETIEWLLQQAEPSVIAATGTGTIPANFTSLNISLRSSGSSMSAPSHYLRTNASNYYFNSNNFGAAASTSQLVTEENAQRGRILFPGAGLMSSENSPPSSMLWNFNHSGNNNVSNNAMLEAKQELGDHHQAVTLDEASSMARKRIRPEQDLLSASTGPIPASQNTTPATFWMVANNNPSSSQGSIHHEHTNSINPMWTIPTIGNAGNMHRGPVAGGSHGLHFMNFASPMAILPGQQLGLGSSTTSHGAADSHLGVLAALNAYRPILGGVAMESTANGQHHPHHGADEGHDRNTNAR |
| *MdTCP47* | MDPKGSKQTQDIPSFLSLPPQPQQQQQPPPQPNNNMNDDKPAEIKDFQIVIADKDESGKKQLAPKRSSNKDRHTKVEGRGRRIRMPALCAARIFQLTRELGHKSDGETIQWLLQQAEPSIMAATGTGTIPASALAAAGGSVSQQGTSLSAGLHQKIDELGGSSGGRTSWAMVGGNLGRPHVAGVGGLWPPVSSFGFQSSSGPPSATTNLGSESSNYLQKIGXPGFDLPVSNMGPMSFTSILGGGNQQQQQLPGLELGLSQDGHIGVLNSQALSQIYQQMGHARVHQQPPQHHQQQHHHHHQQPPSKDDSQGSGQ |
| *MdTCP48* | MIKSPITEADLQEAGAGSSSRDDEANKVSSNPNLSRPSTPWLRLKDPRIVRVSRAFGGKDRHSKVCTIRGLRDRRVRLSVPTAIQLYDLQERLGLNQPSKVVDWLLDAAKHEIDELPPLPMPSXGSFGLNHPSLGLTSSHGDQTNAHAQLSHNHSGEGPSSGIDRSNFWPTDSDALWRAKSKEIVRATRNEXEGNQKDNLGISDDQEQAGNNVDGNSSNTFLRSNTNPPFFPGLLNSSTNMPYAYHNWDHSHQTSNFPLSQLGSHGFQSQTTDLHNFLNVLSLPSTLSLSTTQSYFXSHNTAATGELDPRQFNHLQMLNSSXSTSTSQNLLPNFLSTPALYPSSQTLRAPHLSMVTKLVHSSDNNGSGHHPNKNQEPPSR |
| *MdTCP49* | MGMEGCGGEIVEVQGGHIVRSTGRKDRHSKIYTAKGPRDRRVRLSAHTAIQFYDVQDRLGYDRPSKAVDWLIKKAKSSIDKLVELPPWHPITTSNHAAEADDPFRSSNPNPNGPNPNDMVIAAAEQQSESSTSYNFNFELQRQRQSDNDSNFNIXPXLDSDNIADTMKSFFPTNTSSNAAAASSVDDFRSYPTDPHLISATTQDLGLSLHSFQDQGLNIHHNHTHTQQSSQALFAAAVGTGFDSSSYQRMVAWSNENRGLDGGFVFNSHSHSYXQPXPHNHGGDGNHGSTLQSSFTPSVSTRAWQQQHSSIFGTRFGTSDGTSPVFCXQGEEAENGTISDRPSSTSSLNSTRQL |
| *MdTCP50* | MGIRSPSSSGGVVNTDIVEVVQGSHIVRATGWKDRHSKVCKVKGPRDRCVWLAAHTAIQFYDVQDRLDYDRPSKVVDWLIKKAKAAIDELPPWNPHSTSTIAAAIVVRRKPILFS |
| *MdTCP51* | MSNSEDPNNELTNGGGGGGAMVEQTSQRPSSNGVLALKKPPSKDRHSKVDGRGRRIRMPIICAARVFQLTRELGHKSDGQTIEWLLRQAEPSIIAATGTGTTPASFSTVSVSLRGGHTSSSAPSSDHKPQLLGPTPFILGKRVRGPLDDHDNNHNHDASATKNPSDHHANNDGAVSVVGHSMASMLGPTAGGPGGFWAPHFGQVWSFAATPPPELMDQSAVSHQQQQQHHQQQHSLFLQQQTMGEASAARVGNYLPGHLNLLASLSGGHGNSGQREDDRR |
| *MdTCP52* | MGVERYYQLAKNPTTIGCRKLENGLPEGGEEHQKYSGGRLWSSTTPRECPRLKDPRIVRASRAFGGKDRHSKVCTIRGLQDRQVRLSVPTAIQLYDLQERLGLNQPSKVVDWLLDDDHNQVRLLPG |
| *FvTCP15* | LPQENTTPGGIFGLHQDQFSLVNNSFLPPGPESTMLNLGGDSSNAIVPSPGDYRQHYGYDINNISHPYFVSKDVQVPVVAVKKDRHSKIFTAQGFRDRRVRLSINVAREFFDLQDLLGFDKASTTLEWLLRKSKNAIKDLEKSKCKNSFSSSSTSECDVVLEDNLDKRMRKMKERAKARARARERTREKMCGISKRSPQICSHLRLFNDLEPADMNQERGSTVLTEYHESVALKRKLKAPSGSNYPDRSLELNPQRMEVSSNCCSHINIQFPSAISPNWDINSGVFTCPSICAITANMNLNSSGF |
| *FvTCP7* | MYPLLGNMTSTHDESSSSQPHHPHHDYNSSYFQDHDGVVLSYLLSQQLDTDSSQSADISVAASNQGLIMEEEKIQSSKKKRKAAAAPRKRSSGKKDRHSKIYTAQGPRDRRMRLSVQIARKFFDLQDMLGFDKASKTIDWLFTKSRSAINELKQQLVLRTCSTINSLSTSAESNEVVSQTMQDLKSDSSIGRMINPRKNIRRYRVGKGSRDQARARARERTRQKLRIRLSCQANHDEANPKDLGLLCLGEEAEKHTNHELIEQGRTNSMMRNNADDHKTLMGRSISARSRARFEDDGCEFPGFTGNWGNKMQSIHPRKMTRNVTESFTGNNLLQVENPSTPIMGLTSKYQEPSSTTTSRLSFSSSNSQEQSYSGTYSNSCPINFMPNQDHNPNPIFIATLISPDQNPGQFLKFN |
| *FvTCP10* | MHPPLSFVYFPSPLEDDDVLLHHHHHHELLSLHENTLSASHHHHHQTGTPLTAMVEWDSNKDDQLLIMSKEGEQQQQQQQQQQQQQQQQQILIPTARRSCKKDRHSKIRTARGLRDRRMRLSLDVARNFFGLQDMLGFDKASKTVAWLLNKAGPEIGKVREMSGQNENQSSVSDCEVVSGTDEAATDEIIDKISRKPSSSSCANNRIKRTVRQPKKSAFFNPLAKASRVKARARAKERTREKMERRQPKPCDDVDQAKKMELDLSQLSSWSTFQTGEESGGTQSHNNLNNQSSLEVLAEEEPMSNFQAAGAAGVVHQDLIEIDGHDAAANLVTMGKWSSSSIFHSLQNTSASISQENV |
| *FvTCP8* | MIMEGDNENGIRIRPNFPLQFLDKRKEEEAAASSSYGGAEQPNKAVVVARSSSGDVSTIPEKKPAPPKRTSTKDRHTKVDGRGRRIRMPATCAARVFQLTRELGHKSDGETIEWLLQQAEPAVIAATGSGTIPANFTSLNISLRSSGSSMSAPSHLRTSGYFNPNNSSSLLFGGGASQLMEEQQRRRVLFPGVDLSAPSSMLLNFNSGNVNAMLEAKQEMRDQGNNNSSSTTLALDHMSREGTSIGRKRRPDEQDLLSSSSSQTSHSQLGNYMLQSSTGSIPASQSTTPATFWMVANPSSNNNNQGQAGNMSGGGGSNAHLHHQDPPMWTFPTINGNHGLHFMNFAPPMAILPSQVQLGTSGLSTGGGVGGGAMADSHLGMLAALNAYRPIIGGGAPANGQHPSDDADEGQNDEHSNASQR |
| *FvTCP12* | MEGGDDHFHHHHHHHYQQHHHRPNFPFQLLDQKKEDEAASCSNNSAAASPYPSLAISSAVDPINPLNNNTAPSSTAVTTAVAAEPSKKPPPKRTSTKDRHTKVDGRGRRIRMPALCAARVFQLTRELGHKSDGETIEWLLQQAEPAVIAATGTGTIPANFTSLNISLRSSGSSMSVPSQLRPSYYSPNFSSSIHQSSQNQRRNLFQGLGLSSSDSSSTLLNFQTNTMHSMLQAKQELRDTVSLDLSEAASAAEGSMGRKRRPPEQDLNQMGSGYLLQSSAGAIPASHHSQIPANFWMLANSTNQAAVMSGDPIWTFPSVNNSGLYRGTMSGGLHFMNFPTPVALLPSQQHMGGSGGGGGGDNGGNDGSGGDHHNNMNDGQLNMLTGLNPYRHMSSDSPHEQQASGSHSHHGGDDRHDSTSHHS |
| *FvTCP1* | MEVEEIPAQQPCKFPRIGNGGRGSSSVAKNPDEEDNNGGGGGGGGGREIKTTNHLNGWHHSRIIRVSRASGGKDRHSKVWTSKGLRDRRVRLSVSTAIQFYDLQDRLGYDQPSKAVEWLIKSAADAIAELPSLNTDSFPDTPKQLSDEKRGSSEHGLDSVEVDVDDHPNYHHQMNQQQLCLSKSACSSTSETSKGSGLSLSRPNRVKAARERARERTAKEKEKESNNNYNNNESSRIAQAISQNSSFTELLTGGIGHNSNNSPTGHHHHQNCNGEHVLFHKAAAHQQQQMDYSFGSSSLLGLSPTPARNQHQFSGQIHLGNSLPAQTLFSISGDHHHRSSSSSSHPEMPQHFSFVPDYIPVSAAANGGEYNLNFSMSMSSSGGLAGFNGGTLQSNSSSSPSLLSQLQRFSPMESNVPFFIGAAAAPTMENHHHHHHQFPAGLQLYGDGSRHSDHKGKAKN |
| *FvTCP3* | MGEQSHNNLHHHHHRATASSSSAAARLGLGIRTTTSSSTASEIVEARGGHIIRATGRKDRHSKVCTAKGPRDRRVRLAAHTAIQFYDVQDRLGYDRPSKAVDWLIKKAKAAIDELDELPPWNPNSISVQTSASPTAPPTSAAQETQNNSSLHFSMVEAPSSLSANRRASMVGSGVAELVNQNSSNFLPPSMDSDSIADTIKSFFPMGAATTAETPSFHPSYPPDLLSRTSSQSQDLRLSL  HSFQDPILLHHHQAHQHQTHHQNEQTFLTPNPLGFDSSAATWTEQHHQQQQQAELNRFQRMLAWSNAGGGDSNNNNNNNSGGGGGGFTFNSLPLSQSQSPTLQPFLFGQNQFFSSQRGPLQSSNAPSVHAWIDHNDLSDHQHQMAPSMHNHHHQQSSISGMGFASGVFPGFHIPARIQGEEEHDGISDKPSSASSNSRH |
| *FvTCP14* | MGMKSVGGEIVQVQGGHIVRSTGRKDRHSKVYTAKGPRDRRVRLSAHTAIQFYDVQDRLGYDRPSKAVDWLIKKAKTAIDKLAELPPWHPTGVAATATEGEAPNGAGTSEMVISAEQSESSGYNFQLQRQIGDNTDHNDSNFIPQSLDSDTIADTMKSFFPTSSAASTINFQSYPSDIISRTTNLNPNQDLGLSLHSFQDQGLIHHTQQSHQDTTHHHNDQIQTLFAVPATAGFDSSTYQRMVAWSSDTSSHENNNRGLFFNNMSALPHQQQQALMGQQHSSAFAQRGTLQSSFTPSIRSWSDEIQMASQRTQHPIHHSSIFGTRFASDGLPVFCIPTTRIHGEDADNAVVSDRPSSTSSPNSNHH |
| *FvTCP2* | MTSHSQGLYLQLSQLRDSKKQEEEENEVPNQAGDAQEQQDYEARVEAKQPNMNDGRYGKIVKVQGGHIVRSTKKDRHTKVLTTKGPRDRRFRLAAQTAIEFYDVQDRLGYARPSMAIDWLIQKAKASIEALGNSSTVQQPQQHQGGPAVPETVEIHQHFNESAMNNVSKEPVLGFAEEVLNYATNLSEADFMEMAISQSLMAYNYYTGAGGYNAGDDGECSSFNSSYVSWN |
| *FvTCP6* | MKSGPHLPCAPPLSPPAPVWTSMIREELCFPQPPHSPFPSSSASLSLSLYPPQPTLPFSL  PTGLAARTAVSYRSVSMSNSEAPNNEQLSNGAAAAAMMMERQQQQQQTQQQQTAQQPSSNGVLAVKKPPSKDRHSKVDGRGRRIRMPIICAARVFQLTRELGHKSDGQTIEWLLRQAEPSIIAATGTGTTPASFSTVSVSLRGGASTATSLSNSSTTTSSDHKPLLGGGGPTPFILGKRMRTDDDGKDSDNHHGGNDGGGAVSVVGHHSMSSMLGPAAAAQGGFWAPHFGQVWSFAATPPPELMSQQQSVSQQHSLFLQQQPMGEASAARVGNYLPGHLNLLASLSGGHGNSGRREDDPR |
| *FvTCP16* | MIRSPNTNEADVQAPAGNSSAQNEAGKTSKLALSSSTSRSSTSATVAPWMRLSKDPRIVRVSRAFGGKDRHSKVCTVRGLRDRRVRLSVPTAIQLYDLQDRLGLNQPSKVVDWLLDAAKDDIDQLPPLPMMPPAPGSYGGGGLGLMNHQSLLNITPSSLDQNIGDHQGFRTNIWRSTNDGEEDQENDKDGEESDEDDHGKQEGNVVDHGSSSSNNFLMTRTSTTTNHPLFFPGLLNNSNAMPNYSFHNWDQNQSSNFPLSHQLGSHGFTAHNFNVASLPSTLSLSTGTTQSYFPSHATTDAADHQIDIPRQFDHHMQNSHHQNLLASSVYPSQQSMQRATPQNLMSMITKLAHSSQPNKDQKPPSR |
| *FvTCP11* | MMTDPRGKGFQAKQEGQNNNNNDGNTMSSYNKAASSSTTTSRQWSGFRNPRIVRVSRTFGGKDRHSKVSTVRGLRDRRIRLSVPTAIQLYDLQDRLGLSQPSKVIDWLLEVTDDDIEKLPPLQLLPHHGLLNTTSTAHHQFHHQQQQILNPVNVPFFDVNRRLTEQVVLDESKGKSIKDHDDQALAQKLFPIGNMPSSIPGLLNNAMAYNNYFQNHNSEPSCLSLSQFGSSSHGFPLAPQMDHMMSSNNGLSFSNTSMPMASGSQLFFCPSTATPVPSLFGPYAPYITATTPVGENSSTSNDQQPALRQTSNHMIQLLSSGNTTSQNFAPNNVLMPSLQYSIGSSLRSFPTLDNLKLHSQNHNASSSQPNKDDTGS |
| *FvTCP17* | MDPKASKQPQEIPSFLSLPQPQQQQQQHQQHHQQLPNTMSENNNNNNHNHNNNNNVVKPAEIKDFQIVVADKDETKKQLAPKRSSNKDRHTKVEGRGRRIRMPALCAARIFQLTRELGHKSDGETIQWLLQQAEPSIIATTGTGTIPASALAAAGGSVSQQGSSISAGLYQKTDDLGSSGGRTSWAMVGGNLGRPHVAAATGLWPPAGFGFSSQSSSSGPSTTNLGGTESSSNYLQKIGLPGFDLPVTNMGPMSFTSILGWGSQQLPGLELGLSQDGHLGVLNSQAYQIYQQMGHARVHHHQQQQQQHHQQQHQHQQQQQAPSSKDDSQGSGQ |
| *FvTCP18* | MLQNQEVKPGQVDPTGRGGTDSPPSQPPLVMKEEQEPEQRSSQAITVAVNKPKRTSTKDRHTKVEGRGRRIRMPATCAARIFQLTRELGHKSDGETIRWLLEHAEPAIIAATGTGTIPAIAMSVNGSLKIPTTAPDSGDPAKKRKRPCNSDYVDLNDTVSAGLAPLTTTTQQQPTPPPPQPVAQSMVPMWAVTSNGVVPGTFFMVPQTQSPHMFTFPATANAPSPFLNMSARPISSFVSSMGTNTLSFISPASTPPPASADITHSTVATTTTSLRDKTELQFMSGSSKH |
| *FvTCP19* | MEENQRQSLEPMNPTFSGHQFPAAAKKEEFEEGSLSMGLIPVPASAKPVVKQRSSTKDRHTKVEGRGRRVRMPAACAARVFQLTRELGHKTDGETIRWLLEHAEPAIIAATGTGTVPAIAVSVGGALKIPTSSLANPNGEVSELPRKKRRLHCNSEFVDVEDLSSISSGLAPMATVNYGGIGGGGGGGLVPMWQFGANVPAVPFLMFPNSGWAIPSDSVSGQTMFNLQGRPMSNFPVTALQSGVEVHSCGDVQATSGSILSGGGSCSTSLGVSNSNAVTSSDTSATTAAPMSHTQLLRDLSLDTLDKRELQFLGDLCGNSQAQYSRP |
| *FvTCP9* | MTSYFEDQEDDGGGGGSDRSTSTGDPEENRNSEGNRNGAFTRQLAGFHETAALQGTPLKMEPFDSDPPSDSDHRAHLKLPRAPPSVGMVPLAAMQPAAVRRTSTKDRHTKVEGRGRRIRIPATCAARIFQLTRELGHKSDGETVRWLLEHAEQAIIEATGTGTVPAIAVSVGGTLKIPTTPSNHHNTAEDSPTSAAAKKRKRPSNSEFVDLSDAVSQSSGLAPLGPSAPQPVPQGLVPVWAVGNAGGMMVPANAFWMIPPNGSGQVVSAAAAGPSSQPPQIWALSPTLTPVFNMAAARPISTFVANGGGVEIRAPSPALSNSAVSTSTVGSKAAKKSSSTMAPSVSSSGSGNSNSNNGTKAQMLRDFTLEIYDKQELQLMSRPAGPGSQ |
| *FvTCP13* | MGSEMALQDYLSPDPPHHHHHQPHLPSLKLEDLTQNVAVPAAVLHPTTTTVTTGDNSGKTTAAQQLSRTRRSTNTKDRHTKVNGRGRRVRMPAMCAARIFQLTRELGHRSDGETIEWLLRHAEPSIIAATGTGTQPAQLCTSTPSMPVSIPSLPCRPTVTTASAGQFIYHHLQLPVSQAHQQQQQQQLSCRLDLCQPLPGLDYAVAGNNHGGYQNSHYNNHGFAAGENSHGFTALLLQSGVSDGETPPPPRKVLRGSTREVRF |
| *FvTCP5* | MGTNPWNASRSRNKVLGKASSEDNNTKVDGHGHRTRLPPTCAAWIFQFTRELGHKTDGQTIEWLLKQAEPSILVVTGNSVTPSNTSSTLVSVNMQAGNVVSEDKNSLRKKCTKGGGQPMPPPYDHYLKIITKNYSIEFSVNDVAIDVGIEAQREDERQD |
| *FvTCP4* | MGTVPNFSTKASSSSNQLFHGKASTSVGHGKDEGGECGIRLPKKCVARLVQLTDELGFDDNGQTVEWLLNTVEPSIVAATGRSVSSINTPSTQAPAPSTQATEDMEENATVSLEDKDLVKKKRANAGQPLPLPYDDFMRLVTNYDLEFSANDVAVDVGIPAHEENNNQDGQERLG |
| *BrasTCP13* | MFSSTTNTSSGTVYPFPYNFPLCSSPYNHHPPLPQENRTCGGILGLHHDQSSILNNAFMVPGPEGTIINLGGVPSNAVVSSSGDYHGQQQHGYGNNVYHPHIVSKEVKVPVVAMKKDRHSKIFTAQGLRDRRVRLSINVAREFFDLQDLLGFDKASKTLEWLLTKSTKAIKDLEKSKCNNTNSLSSSSTLECDVVSDMNTEESEDNRKMKEAAAIDHAVRARESRAKARARARERTREKMCSTSRRSPQIFSQLSLFTELEPADMNQEPGSTLLSNYQAEHHESVTVKRKLKAHSDSNYHDRILGLISKSTETGSNCCSHGFKSMAKLWEACTIYDCNDLLQAKHM |
| *BrasTCP10* | MFPHSASSNIINTSTATGNYYESAVSFPHDQSFLIHSRPFPSDITPNLNPNPNPNSNSDS  IISNSKQYQDLEEDLNHHIHPPLSVFYFPSPFEDVDVLLHQHHHLHDHEPLALQETISLAQNHHHHHHQTSFPLTAKMVDWDANKNDQVVINKEGEQQHQQQKQIPMLRKRSCKRDRHSKISTARGLRDRRMRLSLDVARKFFGLQDVLGFDKASKTVEWLLNQACAEIKKVTRERSGQNQNQSSSTTTATGPGARTTSSISECEVVSGTDEVATDDIIDKVSRNPSSSCTKDRIKKIIRKPKKSSSFNPRTKASRERARARARERTRERMGRQSKPCDDVDQATKTKDLSRLNSWSTFETGEESGGTQSHNNNNNNNIHSSLEVLAEVEEPISSFQAAGTSSTTTDHHQDLIAIDGHDAAANMAIMGKWSPYPSIFNYLQNTTGSISQEQHQFADFQFKPWDDNSSTHNLF |
| *BrasTCP6* | MYPPLSSNTTTTTNDENPGTNSSSQPHHHPHNYDYNSPFLEDHDGVILSYLLSQQQFPMVDSGGADATSNLSQHQAAQSSAEISVAASNQALMEEKILISSKKKKKAAAPRKRTSGKKDRHSKICTAQGPRDRRMRLSLQVARKFFDLQDMLGFDKASKTIDWLFTKSKSAIKELKQHLILPRTIGTAKYSTSTESGEVVSKTMMTHCAVEGESSIGGMARDPKKNMRNLRMVAKGSRDEARARARERTREKMRIRSSCQTTHDEENPNDLGLCLGEESKKPTNPTNPQLFEQGGTNSMMINNTNDHKTLIGRSISASRNRPNFEEDGEFTGFTGNWGNINSTKMQSFCHTMTANVIKSFTGNNVLQVENPSSPIMGTTTSHYTKQQEPSRTATSLFSYTSSNSQEQNYSGTSIFLTPIPNTQEVLNPCSINFMPNQDHNPNSIFITTLISQDQNPGQFHKFN |
| *BrasTCP5* | MIMEGDENGIRIRPNFPLQFLDKRKEEEAASASSSYGGEPGSSKAVVVRSNSDVSIVQELPKKPAPPKRTSTKDRHTKVDGRGRRIRMPATCAARVFQLTRELGHKSDGETIEWLLQQAEPAVIAATGTGTIPANFTSLNISLRSSGSSMSAPSHLRTSGYFNPSSSNLLFGGGAAAASQLMEEQHRSRVLFPGVDLSPSSMLLNFNSGNVNAMLEAKQELRDQANNNSTTTLDLDHMSREGTSIGRKRRPDEQDLLSSSSSQTSHSQLGNYMLQSSTGSIPASQSTVPATFWMVANNPSSNNNQGQGGNMSGGGAHLHHQDPPMWTFPTINGNHGLHFMNFAPPMALLPSQVQLGTSGISTGGGGGGGGTVADSHLGMLAALNNAYRPILGGGAPVNGQHPSHDADEEHHDDHSNASQR |
| *BrasTCP8* | MEGGDDHFHHHHHHYQQQQQQQQQQQQHHHRPNFPFQLLDQKKEDEAASCSNNSAAASPYPSLAISSAVDPTNPTASSNTAVTTVVAAEPSKKPPPKRTSTKDRHTKVDGRGRRIRMPALCAARVFQLTRELGHKSDGETIEWLLQQAEPAVIAATGTGTIPANFTSLNISLRSSGSSMSVPSQLRSSYYSPNFSSSIQHSQNQRRNLFQGLGLSSSDSSSTLLNFQTNTMHSMLQAKQELRDTVSLDLSEAASAAEGSMGRKRRPPEQDLNQMGSGYLLQSSAGAIPASHHHSQMPANFWMLANSNNQVMSGDPIWTFPSVNNSGLYRGTMSGGLHFMNFPTPVALLPSQQQLGGSGGGDNGGNDGSGGDHHSNMSEGQLNMLAGLNPYRPMSSESSQQQASGSHSHHGGGDDRHDSTSHHS |
| *BrasTCP4* | MSQQRESKTEEEEEEEEEEEDDDENEAQNQPEDAQEQADNGTKLDTNLANLASKKPSSNNGRYGRIVKVQGGHIVRSIARKDRHSKVYTSKGPRDRRFRLSAQTAIEFYDVQDRLGYDRPSKAIDWLIHKAKAAIKALDDSSATQQPQHHQGCIAEPETVETQQHYHERTMNNVSKEPVLDYAEETLNSASHLSEVNFMEMGWLQSLMAWNYNAGDGGEGCSFNSSSLSLQ |
| *BrasTCP2* | MGESHNNHHRPTPSSAASRLGLGIRTSSSTASEIVETRGHILRATGRKDRHSKVCTAKGPRDRRVRLAAHTAIQFYDVQDRLGYDRPSKAVDWLIKKAKAAIDELEELPPWNPSAQTSSPPVVPISATQDTQNASHHHRFSMVEAPVSSPGNRRTAMVGSGVGELVNHNSSNFLPPSMDSDSIADTIKSFFPMGAATAAETPSFHHSYPPDLLSRTSSQSQDLRLSLHPFQDPILLHHHNQAHHHQNEQAFLSGTANPLGFDGSSATWTEHQQQQADLHRFQRMMTWNNAGADSNNNSGAGAGGFTFNSLPLTQTQSPTLQPFLFGQTQFFSSQRGPLQSSNTPSIHAWIDHNDPSSISDHHHHQMAPSIHHQSSISGMGFASGGFPGFHIPARIQGEEEHDGISDKPSSASSNSRH |
| *BrasTCP7* | MSSSEAPNNELTNGVGMMIEPSSSQRQQQQQQQQQQQQQNQLTTTSGNGVLAVKKPPSKDRHSKVDGRGRRIRMPIICAARVFQLTRELGHKSDGQTIEWLLRQAEPSIIAATGTGTTPASFSTVSVSLRGGASSGAASLSNSSTTSSEHKPLLGGPTPFILGKRMRADDDGKDGEAHHGNNDGGAVSVVGHHSMSSMLAPAAAAQGGFWAPHFGQVWSFAATPPPEMLTQQQSVSQQHSLFLQQQPMGEASAARVGNYLPGHLNLLASLSGGHGNSGRREDDPR |
| *BrasTCP12* | MIKSPNTNEADVQGPAGNSSTHNEAGKISKVTLSSSTSRSSSSTTVAPWMRLSKDPRIVRVSRAFGGKDRHSKVCTVRGLRDRRVRLSVPTAIQLYDLQDRLGLNQPSKVVDWLLEAAKHDIDQLPPLPMMPPAPGSYGGGGLIHHPSVLLNVASSSVDQNIGEQGFRSSLWRPTNDGGDEDQENEKDVEADEADHRKQEGGNVVDHGSSSANNFLMARSSTSTNHPLFFPGLLNNSNTMPNYGFHNWDHDQSPNFPLSHQLRSHGFASQPTDLHNFNVMTLPSTLSLSTGTTQSYFPSHATADAADHQIDIRRQFNHQMQSSHQNLLANSVYPSSQSMQRAAPQNLLSMITKLAHSSQPNKDQEPPSR |
| *BrasTCP9* | MITNSRGKSFQAKQEGQNNNNSDGNIMSSFNKAASSSTTTSRQWSGFRNPRIVRVSRTFGGKDRHSKVSTVRGLRDRRIRLSVPTAIQLYDLQDRLGLSQPSKVIDWLLEVTEDDIDKLPPLQLLPHHGLLNTTSTHHQFLHQQQILNPLNVPFFDVNQRISEQVVLDESKGKSIKANDHHQHDDHDQQALAQKLFPIGNMPSSIPGLLNNAMAYNNYFQHHNSEPSSLSLSQFGSSHGFPLVPQMDHMMSNNSGLSFSTTSMPMAAGSQLFLCPSTATPVPPLFGPYTPYITTTTPVVENSDDDQPALRQTNNHMIQLLSSSSSITSQNFGTNNGLMPSLQYSIGSSLRSFPTLVNPLHSQNNNGSSSQPNKDHTGS |
| *BrasTCP16* | MDSKGSKQQQPQEIPSFLSHPQPQQQPQQQQHHQQPNNNNMSENNNNKPAESKDFQIVVADKEETKKQLAPKRSSNKDRHTKVEGRGRRIRMPALCAARIFQLTRELGHKSDGETIQWLLQQAEPSIIAATGTGTIPASALASAGGSVSQQGISLPAGLHQKIDALGPSNVESSASRTSWGMVGGDLGRPLQTGLWPPTGFGFSQSSGPSTTTNLGSSNYLQKISFPGFDLPVTNMSFTSILGGGGGTTTQQLPGLELGLSQDGPIGVLSSQALSHIYQQMGHARVHHQHQQHHQEAPSSKDDSQGSGGQYQQNPVVESHQGSPGLLLRQVLDRLLVQIRPFTRQENTVIQPHLWRFYFIFSPTYVGCGFICPHIVNFSVLLGAIISWGFLWPLIPQYAGHWYPADLAIFLIVIEIWLKADLTLSIMFVYSESSKQNNLPLVKENLVTMPIIFLPLKWYLVLCSYILVPSLAFCDSYGTDLTDWSLASTYGKIGLFIIASLVGSDGGVRAGLAARGLVGTAMGCVMALTFWLFWTAFDIGSPDGPYKAPYAVIFREMATLGIEGFSELPKHCLAMCCGFFVAALVINHLS |
| *BrasTCP15* | MAMLQNQEFEPDQDAPAVDLGIHGGGVDPTDRTETDSPPAPLSQALVLKEEPEPEQRSPALTVAVNKPKRSSTKDRHTKVEGRGRRIRMPATCAARIFQLTRELGHKSDGETIRWLLEHAEPAIIAATGTGTVPAIAMSVNGALKIPTTAPDPKPGDPDSKKKRKRPCNSDYVDLNDGVSYSVSAGLAPLTTAQQQQQQPTPPQPQVVPQNVVPMWAISSNGVVPGTFFMVPPMASTQSPHIFTFPATAAASPFLNISARPISSFVSSMGNIVTPVSNTLSFMAPTPTPASATITNSTIATTTAQTLRDFSLEIRDKTELQFMSGSSKH |
| *BrasTCP17* | MDANQMPSLEENNGLINPTDYSSYDSNPSVSDPKVPGTAPSMKDEFDEGSLSMGLIPVPVSAALVPAKKRSSTKDRHTKVEGRGRRVRMPAACAARVFQLTRELGHKSDGETIRWLLEHAEPAIIAATGTGTVPAIAVSVGGTLKIPTSSPARPNGEVSERLRKKRKRQSNSDFMDVNDLSSVSSGLAPIAPMSCGGGGALVPMWQLGANVPAGSFFMFPNSGWAIPATDSISAHQIFNLQGRPISNFVSALQSEVQGCDVQATSGSISSGGGGGSCSSSLGANNSNAVSSSDTSATTAAPISTTSHTQLLRDFSLGIYDKREPRVQGGFSGRV |
| *BrasTCP14* | MGSEMALQDYLSPDPHQPQQQFPSLKREDPNPNVVVPAAVLTSTTTVTTVTTADNSGKTQQLTRPRRSASTKDRHTKVNGRGRRVRMPAMCAARIFQLTRELGHRSDGETIEWLLRHAEPSIIAATGTGTHPAQMCTSTPAMSASMPSLPCRSQSINSGAGQFMYQLHHPVSQAQQQQQLSCRLDLCQPLAGLDYEVAVAGNNNGGYQNSHGGYSHTFTALLLQSGVGDGEEASAGGGGSQGLDQ |
| *BrasTCP11* | MTSYFEDQEDDGGGTSDLSTSTGEPEENRNTEDNRNGVFSRQLANFDETAALQGMPLKMEPIDSDPPSDSDRAHRQPPQAGMVPVEAMEVQMPMPTVSTVTRRSSTKDRHTKVEGRGRRIRIPATCAARIFQLTRELGHKSDGETVRWLLEHSEQAIIEATGTGTVPAIAVSVGGTLKIPTTPSNNPAEDSPASMAKKRKRPSNSEFVDLSDAVSQSSGLAPVGPSTPQAVPQGLVPVWAVGNAGMMVPANAFWMIPPTAANGAGQVVSAAGPSGQQPQIWALSPTMTPVFNMAAARPISSFVANSGVEVTSTVGSKAANKSSSTMAPSVSSSGSGSGSGTKTQMLRDCTLQIYDKQELQFIGRPAGPGNQ |
| *BrasTCP1* | MARETELSTIASSSRNTVLHGKASSRGGRHKKVEGRQCRIRIEESCAARIFQLSCDLGHKTNGRTIEWLLKQAEPSIIAATGTGVTPINTPSTPLVAVNLQENVVSHEDKDLVQKKRTRCCQPLPPPYDHYMRMITNNYSMEFSASDVAVDVGIQTQNET |
| *BrasTCP3* | MWLIFSLIPAAYASDKGILKIADLELGRSFTVPLKRYMHEISILWYKAPAVLLSSTPYSIDVDMWADKGDSIKASYLHHLSKLNGGAIVGEVVRKFSTNENTLTVGGSYVVDPHTTFKARLNNHINLRALLQYQLSPKNIFTLSGSLHNKTLHKNSKRALTDAEEAIKLNPTNLKDRLGYDRLSKAIDWLIDKAKAAIEAVDDSYATYHKKANFMKMGWLKSMMALNYNADSHLSTSSIPSKPDQFLSYPINDVNRIWSRQILTKQEIVHEQQQTESAKRISISRPTWSRRPSHSTEPWTPPFRSRTRL |
