# Supplementary material for: Comparative Genomic Analysis of TCP Genes in Six Rosaceae Species and Expression Pattern Analysis in Pyrus bretschneideri
Source: Front Genet. 2021 May 17;12:669959. doi: 10.3389/fgene.2021.669959 (PMC8165447; doi:10.3389/fgene.2021.669959)
Supplement: Supplementary Table 3 — Primers for vector construction. [file Table_3.docx]

**Table S3 Primers for vector construction.**

| **Gene name** | **Primer sequences 5’** | **Primer sequences 3’** |
| --- | --- | --- |
| ***PbTCP6*** | **ATGCCTACTTCAAAATCGGC** | **CTAATGCAGAGATACATGCG** |
| ***PbCCR13*** | **ATGGACTTATCAAATTTCCAACCC** | **TCATTGAGAACTGTTTGGGGC** |
| ***PbCCR17*** | **ATGGATCCCAAGGGCTCAAAG** | **CTACTGTCCGGATCCTTGAG** |
| ***PbTCP6-1304*** | **CCCATGGGGATGCCTACTTCAA** | **CTAGACTAGTCTAATGCAGAGAT** |
| ***PbCCR13-1304*** | **CCCATGGGGATGGACTTATCA** | **CTAGACTAGT TCATTGAGAAC** |
| ***PbCCR17-1304*** | **CCCATGGGGATGGATCCCAAG** | **CTAGACTAGCTACTGTCCGGA** |
