# Supplementary material for: Comparative Genomic Analysis of TCP Genes in Six Rosaceae Species and Expression Pattern Analysis in Pyrus bretschneideri
Source: Front Genet. 2021 May 17;12:669959. doi: 10.3389/fgene.2021.669959 (PMC8165447; doi:10.3389/fgene.2021.669959)
Supplement: Supplementary Table 4 — Basic information of TCP genes in five Rosaceae species (Prunus mume, Rubus occidentalis, Fragaria vesca, Prunus avium, and Malus domestica). [file Table_4.docx]

| **Gene name** | **Gene ID** | **Chromosome** | **AA** | **KD** | **pI** | **GRAVY** | **Preditced**  **subcellular**  **localization** |
| --- | --- | --- | --- | --- | --- | --- | --- |
| **MdTCP1** | **MDP0000123919** | **chr1** | **348** | **35.97** | **6.97** | **-0.351** | **nucl** |
| **MdTCP2** | **MDP0000594000** | **Chr2** | **170** | **19.52** | **8.39** | **-0.791** | **nucl** |
| **MdTCP3** | **MDP0000681033** | **Chr4** | **189** | **21.19** | **10.65** | **-0.625** | **nucl** |
| **MdTCP4** | **MDP0000393985** | **Chr4** | **196** | **22.00** | **7.17** | **-0.479** | **nucl** |
| **MdTCP5** | **MDP0000182310** | **Chr5** | **240** | **27.59** | **6.31** | **-0.946** | **nucl** |
| **MdTCP6** | **MDP0000259723** | **Chr5** | **240** | **27.59** | **6.31** | **-0.946** | **nucl** |
| **MdTCP7** | **MDP0000534647** | **Chr5** | **383** | **42.81** | **9.27** | **-0.652** | **nucl** |
| **MdTCP8** | **MDP0000927314** | **Chr5** | **486** | **53.09** | **8.65** | **-0.871** | **nucl** |
| **MdTCP9** | **MDP0000920127** | **Chr5** | **486** | **53.14** | **8.85** | **-0.871** | **nucl** |
| **MdTCP10** | **MDP0000763497** | **Chr5** | **486** | **53.14** | **8.85** | **-0.871** | **nucl** |
| **MdTCP11** | **MDP0000287069** | **Chr5** | **484** | **52.88** | **7.27** | **-0.877** | **nucl** |
| **MdTCP12** | **MDP0000280252** | **Chr6** | **335** | **34.90** | **5.58** | **-0.305** | **nucl** |
| **MdTCP13** | **MDP0000877369** | **Chr6** | **425** | **45.45** | **6.98** | **-0.756** | **nucl** |
| **MdTCP14** | **MDP0000531313** | **Chr6** | **425** | **45.43** | **6.98** | **-0.757** | **nucl** |
| **MdTCP15** | **MDP0000120671** | **Chr6** | **374** | **41.69** | **9.02** | **-0.741** | **nucl** |
| **MdTCP16** | **MDP0000219838** | **Chr6** | **538** | **60.97** | **9.28** | **-0.812** | **nucl** |
| **MdTCP17** | **MDP0000199422** | **Chr7** | **233** | **26.24** | **9.6** | **-0.839** | **nucl** |
| **MdTCP18** | **MDP0000260056** | **Chr7** | **362** | **37.48** | **5.99** | **-0.345** | **nucl** |
| **MdTCP19** | **MDP0000617746** | **Chr8** | **147** | **16.60** | **9.16** | **-0.629** | **nucl** |
| **MdTCP20** | **MDP0000253526** | **Chr8** | **147** | **16.60** | **9.16** | **-0.629** | **nucl** |
| **MdTCP21** | **MDP0000319266** | **Chr8** | **608** | **63.87** | **8.87** | **-0.631** | **nucl** |
| **MdTCP22** | **MDP0000523096** | **Chr9** | **321** | **34.31** | **8.67** | **-0.798** | **nucl** |
| **MdTCP23** | **MDP0000130524** | **Chr9** | **147** | **16.68** | **10.08** | **-0.743** | **nucl** |
| **MdTCP24** | **MDP0000692406** | **Chr9** | **383** | **42.11** | **7.96** | **-0.821** | **nucl** |
| **MdTCP25** | **MDP0000442611** | **Chr9** | **349** | **38.22** | **6.64** | **-0.720** | **nucl** |
| **MdTCP26** | **MDP0000264920** | **Chr10** | **377** | **39.07** | **6.18** | **-0.355** | **nucl** |
| **MdTCP27** | **MDP0000184743** | **Chr10** | **612** | **69.01** | **7.36** | **-0.915** | **nucl** |
| **MdTCP28** | **MDP0000238683** | **Chr10** | **287** | **30.37** | **8.86** | **-0.689** | **nucl** |
| **MdTCP29** | **MDP0000189749** | **Chr10** | **327** | **36.37** | **8.13** | **-0.635** | **nucl** |
| **MdTCP30** | **MDP0000243495** | **Chr11** | **598** | **65.73** | **6.55** | **-0.780** | **nucl** |
| **MdTCP31** | **MDP0000139807** | **Chr12** | **222** | **24.32** | **6.66** | **-0.476** | **nucl** |
| **MdTCP32** | **MDP0000535805** | **Chr12** | **301** | **32.23** | **5.41** | **-0.494** | **nucl** |
| **MdTCP33** | **MDP0000173048** | **Chr13** | **477** | **53.21** | **8.96** | **-0.843** | **nucl** |
| **MdTCP34** | **MDP0000242185** | **Chr13** | **399** | **42.55** | **6.73** | **-0.602** | **nucl** |
| **MdTCP35** | **MDP0000374900** | **Chr13** | **250** | **26.95** | **9.67** | **-0.592** | **nucl** |
| **MdTCP36** | **MDP0000202241** | **Chr13** | **222** | **25.38** | **9.91** | **-0.450** | **nucl** |
| **MdTCP37** | **MDP0000693146** | **Chr13** | **245** | **27.27** | **9.89** | **-0.409** | **nucl** |
| **MdTCP38** | **MDP0000210785** | **Chr14** | **398** | **42.18** | **9.71** | **-0.647** | **nucl** |
| **MdTCP39** | **MDP0000155433** | **Chr14** | **371** | **41.25** | **7.32** | **-0.755** | **nucl** |
| **MdTCP40** | **MDP0000224810** | **Chr14** | **479** | **54.02** | **6.56** | **-0.908** | **nucl** |
| **MdTCP41** | **MDP0000617459** | **Chr15** | **263** | **29.18** | **10.64** | **-0.769** | **nucl** |
| **MdTCP42** | **MDP0000247249** | **Chr15** | **334** | **36.75** | **6.18** | **-0.095** | **chlo** |
| **MdTCP43** | **MDP0000515080** | **Chr15** | **530** | **55.58** | **6.37** | **-0.657** | **nucl** |
| **MdTCP44** | **MDP0000608645** | **Chr15** | **530** | **55.41** | **6.71** | **-0.606** | **nucl** |
| **MdTCP45** | **MDP0000272980** | **Chr16** | **416** | **46.62** | **9.01** | **-0.881** | **nucl** |
| **MdTCP46** | **MDP0000319941** | **Chr16** | **402** | **42.97** | **6.83** | **-0.594** | **nucl** |
| **MdTCP47** | **MDP0000915616** | **Chr17** | **314** | **33.46** | **8.68** | **-0.757** | **nucl** |
| **MdTCP48** | **MDP0000320363** | **Chr17** | **380** | **41.78** | **6.81** | **-0.774** | **nucl** |
| **MdTCP49** | **MDP0000916623** | **Chr17** | **354** | **38.77** | **5.98** | **-0.790** | **nucl** |
| **MdTCP50** | **MDP0000149841** | **Chr0** | **115** | **12.82** | **9.79** | **-0.234** | **nucl** |
| **MdTCP51** | **MDP0000851695** | **Chr0** | **280** | **29.71** | **8.08** | **-0.710** | **nucl** |
| **MdTCP52** | **MDP0000373350** | **Chr0** | **126** | **14.34** | **9.51** | **-0.800** | **nucl** |
| **PmTCP1** | **Pm003922** | **Chr2** | **516** | **53.42** | **7.21** | **-0.596** | **nucl** |
| **PmTCP2** | **Pm006518** | **Chr2** | **423** | **44.71** | **7.00** | **-0.633** | **nucl** |
| **PmTCP3** | **Pm007054** | **Chr2** | **269** | **28.72** | **9.51** | **-0.529** | **nucl** |
| **PmTCP4** | **Pm010118** | **Chr3** | **287** | **32.61** | **6.42** | **-0.974** | **nucl** |
| **PmTCP5** | **Pm010440** | **Chr3** | **499** | **54.19** | **7.29** | **-0.894** | **nucl** |
| **PmTCP6** | **Pm011369** | **Chr3** | **439** | **47.64** | **6.79** | **-0.691** | **nucl** |
| **PmTCP7** | **Pm015499** | **Chr4** | **417** | **46.79** | **8.48** | **-0.777** | **nucl** |
| **PmTCP8** | **Pm016049** | **Chr4** | **376** | **41.4** | **6.59** | **-0.787** | **nucl** |
| **PmTCP9** | **Pm016133** | **Chr4** | **280** | **29.58** | **9.33** | **-0.5** | **nucl** |
| **PmTCP10** | **Pm018100** | **Chr5** | **296** | **31.68** | **7.15** | **-0.408** | **nucl** |
| **PmTCP11** | **Pm019399** | **Chr5** | **382** | **39.69** | **6.25** | **-0.414** | **nucl** |
| **PmTCP12** | **Pm023748** | **Chr7** | **332** | **34.39** | **5.38** | **-0.37** | **nucl** |
| **PmTCP13** | **Pm024934** | **Chr7** | **376** | **40.36** | **7.46** | **-0.818** | **nucl** |
| **PmTCP14** | **Pm024946** | **Chr7** | **387** | **42.93** | **6.95** | **-0.91** | **nucl** |
| **PmTCP15** | **Pm028040** | **scaffold103** | **463** | **50.63** | **6.04** | **-0.202** | **cyto** |
| **PmTCP16** | **Pm029002** | **scaffold205** | **502** | **55.7** | **8.61** | **-0.887** | **nucl** |
| **PmTCP17** | **Pm029788** | **scaffold327** | **335** | **37.09** | **7.30** | **-0.777** | **nucl** |
| **PmTCP18** | **Pm030202** | **scaffold475** | **448** | **51.01** | **6.42** | **-0.951** | **nucl** |
| **PmTCP19** | **Pm030138** | **scaffold475** | **229** | **24.93** | **5.98** | **-0.604** | **nucl** |
| **BrasTCP1** | **Bras_T13729** | **chr3** | **160** | **17.72** | **8.65** | **-0.526** | **nucl** |
| **BrasTCP2** | **Bras_T10040** | **chr3** | **417** | **45.46** | **6.79** | **-0.713** | **nucl** |
| **BrasTCP3** | **Bras_T17150** | **chr3** | **309** | **35.16** | **9.73** | **-0.416** | **cyto** |
| **BrasTCP4** | **Bras_T05207** | **chr3** | **231** | **26.15** | **4.87** | **-1.124** | **nucl** |
| **BrasTCP5** | **Bras_T00084** | **chr4** | **420** | **44.56** | **6.63** | **-0.585** | **nucl** |
| **BrasTCP6** | **Bras_T02625** | **chr4** | **463** | **51.51** | **9.13** | **-0.837** | **nucl** |
| **BrasTCP7** | **Bras_T17955** | **chr4** | **285** | **30.16** | **9.25** | **-0.620** | **nucl** |
| **BrasTCP8** | **Bras_T03227** | **chr5** | **423** | **45.67** | **6.81** | **-0.807** | **nucl** |
| **BrasTCP9** | **Bras_T03350** | **chr5** | **379** | **41.87** | **8.62** | **-0.621** | **nucl** |
| **BrasTCP10** | **Bras_T03441** | **chr5** | **472** | **53.29** | **6.84** | **-0.921** | **nucl** |
| **BrasTCP11** | **Bras_T14862** | **chr5** | **371** | **38.98** | **5.53** | **-0.459** | **nucl** |
| **BrasTCP12** | **Bras_T20183** | **chr6** | **369** | **40.46** | **6.81** | **-0.740** | **nucl** |
| **BrasTCP13** | **Bras_T04814** | **chr6** | **346** | **38.62** | **8.64** | **-0.639** | **chlo** |
| **BrasTCP14** | **Bras_T13193** | **chr6** | **245** | **26.18** | **7.12** | **-0.566** | **nucl** |
| **BrasTCP15** | **Bras_T15055** | **chr7** | **329** | **35.14** | **7.02** | **-0.376** | **nucl** |
| **BrasTCP16** | **Bras_T07786** | **chr7** | **590** | **64.36** | **7.30** | **-0.108** | **plas** |
| **BrasTCP17** | **Bras_T09502** | **chr7** | **334** | **34.79** | **8.35** | **-0.301** | **nucl** |
| **PaTCP1** | **Pav_sc0003685.1** | **chr1** | **502** | **55.84** | **8.61** | **-0.902** | **nucl** |
| **PaTCP2** | **Pav_sc0000220.1** | **chr1** | **425** | **44.90** | **7.01** | **-0.601** | **nucl** |
| **PaTCP3** | **Pav_sc0000555.1** | **chr1** | **268** | **28.61** | **9.51** | **-0.523** | **nucl** |
| **PaTCP4** | **Pav_sc0001673.1** | **chr2** | **206** | **21.73** | **6.72** | **-0.210** | **nucl** |
| **PaTCP5** | **Pav_sc0000618.1** | **chr3** | **417** | **46.57** | **8.29** | **-0.755** | **nucl** |
| **PaTCP6** | **Pav_sc0001080.1** | **chr3** | **308** | **33.08** | **9.01** | **-0.739** | **nucl** |
| **PaTCP7** | **Pav_sc0000037.1** | **chr4** | **292** | **33.42** | **5.67** | **-1.055** | **nucl** |
| **PaTCP8** | **Pav_sc0001340.1** | **chr4** | **378** | **40.99** | **6.36** | **-0.738** | **nucl** |
| **PaTCP9** | **Pav_sc0002264.1** | **chr4** | **457** | **50.90** | **6.34** | **-0.879** | **nucl** |
| **PaTCP10** | **Pav_sc0003135.1** | **chr4** | **492** | **53.44** | **7.31** | **-0.875** | **nucl** |
| **PaTCP11** | **Pav_sc0000877.1** | **chr5** | **389** | **41.58** | **9.36** | **-0.633** | **nucl** |
| **PaTCP12** | **Pav_sc0000358.1** | **chr5** | **388** | **42.86** | **6.95** | **-0.855** | **nucl** |
| **PaTCP13** | **Pav_sc0000383.1** | **chr5** | **506** | **57.58** | **6.46** | **-0.923** | **nucl** |
| **PaTCP14** | **Pav_sc0002358.1** | **chr0** | **376** | **41.44** | **6.54** | **-0.761** | **nucl** |
| **FvTCP1** | **FvH4_3g05220.1** | **Chr3** | **460** | **49.95** | **7.49** | **-0.880** | **nucl** |
| **FvTCP2** | **FvH4_3g08160.1** | **Chr3** | **231** | **25.83** | **5.29** | **-0.806** | **nucl** |
| **FvTCP3** | **FvH4_3g18740.1** | **Chr3** | **439** | **47.79** | **6.69** | **-0.755** | **nucl** |
| **FvTCP4** | **FvH4_3g31470.1** | **Chr3** | **175** | **18.75** | **4.62** | **-0.566** | **nucl** |
| **FvTCP5** | **FvH4_3g31480.1** | **Chr3** | **159** | **17.55** | **7.79** | **-0.712** | **cyto** |
| **FvTCP6** | **FvH4_4g06720.1** | **Chr4** | **360** | **37.94** | **9.30** | **-0.503** | **nucl** |
| **FvTCP7** | **FvH4_4g28170.1** | **Chr4** | **414** | **46.76** | **9.55** | **-0.915** | **nucl** |
| **FvTCP8** | **FvH4_4g31520.1** | **Chr4** | **421** | **44.59** | **6.91** | **-0.622** | **nucl** |
| **FvTCP9** | **FvH4_5g01340.1** | **Chr5** | **389** | **40.43** | **8.67** | **-0.463** | **nucl** |
| **FvTCP10** | **FvH4_5g12710.1** | **Chr5** | **358** | **40.28** | **6.82** | **-0.860** | **nucl** |
| **FvTCP11** | **FvH4_5g13710.1** | **Chr5** | **376** | **41.44** | **8.82** | **-0.649** | **nucl** |
| **FvTCP12** | **FvH4_5g15150.1** | **Chr5** | **423** | **45.40** | **6.74** | **-0.749** | **nucl** |
| **FvTCP13** | **FvH4_6g16170.1** | **Chr6** | **263** | **28.68** | **9.12** | **-0.611** | **nucl** |
| **FvTCP14** | **FvH4_6g27030.1** | **Chr6** | **366** | **40.31** | **6.38** | **-0.697** | **nucl** |
| **FvTCP15** | **FvH4_6g45410.1** | **Chr6** | **305** | **34.29** | **9.07** | **-0.600** | **nucl** |
| **FvTCP16** | **FvH4_6g46730.1** | **Chr6** | **366** | **40.19** | **6.37** | **-0.812** | **nucl** |
| **FvTCP17** | **FvH4_6g53830.1** | **Chr6** | **333** | **36.06** | **8.97** | **-0.867** | **nucl** |
| **FvTCP18** | **FvH4_7g12810.1** | **Chr7** | **289** | **30.86** | **9.51** | **-0.482** | **nucl** |
| **FvTCP19** | **FvH4_7g28770.1** | **Chr7** | **327** | **34.34** | **6.66** | **-0.241** | **nucl** |
