# Supplementary material for: Comparative Genomic Analysis of TCP Genes in Six Rosaceae Species and Expression Pattern Analysis in Pyrus bretschneideri
Source: Front Genet. 2021 May 17;12:669959. doi: 10.3389/fgene.2021.669959 (PMC8165447; doi:10.3389/fgene.2021.669959)
Supplement: Supplementary Table 6 — Ka/Ks analysis of the TCP homologous gene pairs from Pyrus bretschneideri and Malus domestica. [file Table_6.docx]

**Table S6 Ka/Ks analysis of the *TCP* homologous gene pairs from *Pyrus bretschneideri* and *Malus domestica*.**

| **Duplicated Pairs** | **Ka** | **Ks** | **Ka/Ks** | **Purifying Selection** | **Duplicated type** |
| --- | --- | --- | --- | --- | --- |
| ***PbTCP3-PbTCP20*** | 0.0506 | 0.1676 | 0.301909308 | Yes | Segmental |
| ***PbTCP4-PbTCP22*** | 0.0809 | 0.1889 | 0.428268925 | Yes | Segmental |
| ***PbTCP5-PbTCP10*** | 0.0728 | 0.2104 | 0.346007605 | Yes | Segmental |
| ***PbTCP6-PbTCP18*** | 0.0797 | 0.1695 | 0.47020649 | Yes | Segmental |
| ***PbTCP9-PbTCP27*** | 0.0414 | 0.1217 | 0.340180772 | Yes | Segmental |
| ***PbTCP11-PbTCP31*** | 0.0042 | 0.0202 | 0.207920792 | Yes | Segmental |
| ***PbTCP28-PbTCP29*** | 0.3508 | 0.5706 | 0.614791448 | Yes | Segmental |
| ***MdTCP15-MdTCP39*** | 0.0416 | 0.1451 | 0.286698828 | Yes | Segmental |
| ***MdTCP22-MdTCP47*** | 0.0183 | 0.1244 | 0.147106109 | Yes | Segmental |
| ***MdTCP24-MdTCP48*** | 0.4936 | 0.5445 | 0.906519743 | Yes | Segmental |
| ***MdTCP28-MdTCP51*** | 0.0241 | 0.2488 | 0.096864952 | Yes | Segmental |

对于基因复制事件的确定，主要依赖于以下几条原则：（1）两条基因处于进化

树的同一分支上，且相匹配部分的氨基酸序列相似度在 80%以上；（2）这两个基因位于同一条染色体上且距离至少在 200kb 以上，认为这两个基因为串联重复基因；（3） 若两个基因位于不同染色体上，则被定义为片段复制事件。使用 DnaSP v5.0 软件计 算出一个复制基因对的非同义（Ka）和同义替换（Ks）值，并计算出 Ka/Ks 比例以 确定基因复制事件的类型（Ka/Ks>1 为阳性选择，Ka/Ks<1 为阴性选择，Ka/Ks=1 为 中性选择）。再次利用 DnaSP v5.0 软件对基因复制事件进行滑动窗口分析，以确定在每个氨基酸位点的突变选择模式，具体参数为：窗口大小 150bp，每步移动 9bp[112]

砀山酥梨TCP基因家族中鉴定了8个基因复制事件，在苹果中共鉴定了4个基因复制事件，分别涉及8个复制基因对和4个复制基因对。12个基因复制事件中Ka/Ks值均小于1，最大值为0.818181818（***PbTCP25-PbTCP26***），最小值为0.096864952（***MdTCP28-MdTCP51***），表明TCP家族基因在进化过程中主要受到purifying selection。根据这些基因复制事件类型的分析结果，在12个复制基因对中，共鉴定到9个片段基因复制事件，1个串联基因复制事件，还有2个基因对未定位无法判断基因复制事件的类型。这说明，‘砀山酥梨’TCP基因家族的扩张以片段复制为主要驱动力，串联复制为次要动力。在CIN和CYC亚家族中鉴定到6个和3个基因复制事件均为片段复制事件，唯一一个串联复制发生在CYC亚家族中。

=
