# Supplementary material for: Comparative Genomic Analysis of TCP Genes in Six Rosaceae Species and Expression Pattern Analysis in Pyrus bretschneideri
Source: Front Genet. 2021 May 17;12:669959. doi: 10.3389/fgene.2021.669959 (PMC8165447; doi:10.3389/fgene.2021.669959)
Supplement: Supplementary Table 7 — All TCP gene promoter sequences list. [file Table_7.docx]

| Gene  ID | Promoter sequence |
| --- | --- |
| Pbr006641.1 | TTTTCTTCTCTTTGTCCTCCTTTATAGTTCAAATGTTTACGCTTTTCTTTTTCAAGAAACCTCAATATTTATTCAGTTGGAAAAAAATTCCACCTAGACAGACGATCGAGAACATAAACTTTATTCCTCATGGTTCAATTGGAGACCATATAGAAAAATAAGAAAAAAGAAAAATATATGAAAAGAGGGGCATAAAAACAAAGCGGGGATAGTTCAAGTGTGTGATGCAATTAGAGAGAATAGGGATAAATAGTTTTATATGTATAGTTAGGCTGCAGATGAATATGTCAATTCCAATGATTGATTAATTGGAATCATTTATTTTTGAAGTGTACTCACACAACCTAACATATATGTGTGATCGAGTCATTGTTAAAATTGGACCACGCATCTTGAGTATTTGGATGTGGGAGGAAAACTTCAGCTTACTATAAGGCTTTGTCCACATGAACATGTTCATCTTCCTTGGTACTTTAGGCGTGCATGTGAAAGGGATATATCTAAAACCTTTAATTTGTTAATTGTCGATAAAGCAACACTCACCACTTTCGAATGTGCAACATCCATATGGTCCCCAGTTGAAATTTTCTAATTTTGTATTTGATGTATATAATCATATGTTGCATGTACATATGTACAGGTAAACATTACATTTACATATGTTGCTCACAAGACGTGCCATATCTAATGTGATTTTAGCAGCTAGCTAGAGATTTTCAGATTATTAGCCCTCCTAAATCCTAGTTAACTAATTGTTATTGTCACGATGACTGTTTAACCTTATGCATGCGTGATGAGAAAAATGATGAGATATCGACTAAGTTGATGGTGAGAGTGCCACGTGGCAGGGTATGTACCACTGCTTTATCTTTCCTTGTAGCATGGTCGGTGGGATTCCGGCGCCCTCCTTTTCCTCTCAACTAGCTCCCTACTTCTTCTTCTTCTACAAGTTTATTCTCATATTCTCCACATACTTGCAGAATCATTTAATCTTCAAAAGTACAGGCGGCTGCTTGGTTGGTTTTAGTAGTTCTCCACCAAACTCACAAAGTCAATTTAGCTGAGAAATTCAAAACCATATATATTTTCTTTGCCATTCCGTGTAGTGCTGTATATCATCAAGCACCAAAATCTATCTGCAAAACAAGAAGCAGAAAATGTTTACTTTAATGTAAGTTGGGTCAATTTCAAGTAAGTAGTTCAAAATCATTACCATATGCTGGTTGGGTAACCCTAGGGTACTTCATAATTATTAAATAACACATACCTAGCTTTATATTTGTTGCTTTATTTTAATTCGATTAACATATGTATGCGAGACAAGTGAACTTCGGATTCCTGGAAAAAATATTTCAATAGCTTGTGAATTTTGAGATACTTTTGAATTTTGTTCATGAAACAATTCATGAAAGCTGTATATATATTTGGTTTTTTATTTTGTGGATCATCATACAGGCATGTCATAGATATTAGCTTAGCAGCAAACACTTTTTTTGTTCATTTATTTTCTTTATAATTCATTTCTTTCATGATTGATGGTGTAGCAGATCATGCTAAAAAAAAAAAGAAAGCACATGAAGAAGAGAAGCATATCATGATTAATTCGTGAAGTTTATCATCGCAAATAGAATAGAAATCTGGGTTTTGAGCTGAAATGATGAACCAATGACGAGAGGGTGCATAACAGCATAAG |
| Pbr006477.1 | TTTCATGCATATACATAATTATTAGCAGCTTCAACTTATCACCAAGTATAAATCAATTATGTTGCCTACAAGGCGTATCATATATAATGTGATTTGAGCAGCTAGGTAGAGATTTTAAGTTTAATAGCCCTCTTAAATCCTAGTTTTCTTTTGAAAAAAAAAATCCTTGTTTTCTTCTTTAAAAAAAAAGAGAGATCAGTTGGTGGCTTCATCACGAAATCAGTGGCATAGCTAGCATAGGGTCATTAGTTGTCCTTGACCCCAACAATTTTGAAAACTCCATGTATATTTGTTTATGTATTATATTTGACCCTTATAAACACCTAAGGCAAACTAAATTACAATCTCCCCCTCCTACTTCATGTGTGCGACTCTATTGTGCAATCATGTGCTCGATGAAATTCTTTAGTTTTTCTCTCTTCTTGTGTGCGATCATGTGCTCGATTAAATACCTTAGTTGAAAAGCCTAGGCAATCTTCAATATTACTCCACAGTATCATGCATCAACCCTAGACAAGCATTTACTAATAATAGTAGTTGTGATATACGTTAGCATAAACTTTCGACATATGTTGACAGGAATTGGCAGTGCTTCGTAAGTGCTCGATGAATGGCTAACGAAGTTTTTTTGGTTGATATAATGACCCCATTTTTCAAAATCATGGCTACGGCACTGACAACAACCCACTTTTTTCGAAGGTCAGGAAGACTTATAAAGACATTCAGCCTTCTCATAGTCATCATCATGTGATTTAATATCATAAAGTATCGTACTTAGAACACACCGTGTGAAATAAAAGTTTGAAATTCATCTTCAGTTAACTCCTAAATCATAGTTAATTAACTCCTATTTTGTCACAATTAATGATTATTTAACCTTATGCATGCGTGATGAGGAAATGATGAGATATCGACTAGCTACCATAGCTACACCCACATTCGTTTGAGTTGATGGTGAGAGTGCCACGTGGCATTGTATGTACCACTGCTTTATCTTTCCTTGGGCATGGTCGGTGGAATTCCGGCGCCTTACTTTTTCCTCTCAAACGGTTCTCTCTACTTCTTCTTCAACAAGTTTTATTCTCATATTCTCCACATACTTGCAGAATAATTTAATCTTCAAAAGTACAGACGGCTGTTTGGTTGATTTTAGTAGTTCTCTACTAAACTCACAAAGCCAATTTCAAGTTGAGAAAATCACAACCACATACCTTTCTTTGCCATTCTGTGTAATACTGTGTATTCAACTCACCAAGCACCGAAAACTGTCTGCAAAACAAGAAGCAGAAAATGTTTACTTTCATGTAAGTTGGGTCAATTTCAAGTAATATGTTGGTTGGGTAACCCTAGAATACTTCATAATTATAAAATAATTAGCATCACATACCTAGCTTTATATTTGTTGCTTTGCTTTAATTCCATTAATATATATACGCGAGACAAGTGAACTTGGAATTCCTTAACTAAACATTTCTACAGCTTGTGAATCGCTTCTTCTTTTGAGCTCCTTTTTAATTTCGTTCATGAAACAATTCATGAAAGTTGTATTAGTTTCGTTTTTAATTTTGTGGATCATCACATATATGTCATAGATATTAGCTAGCTTAATTAGTAGGAAACACTTTTTGATAATTTTTATTTTTTTTATAATTCATTTCTTTCATGATTGATGGTGTACCAGATTATGATAAAAAAAGAAAGCACATGAAGAAGAGAAGCATATCATGATTAATTCGTGAAGTTATCATTGCAAAGAGAATAGAAATCTGCGTGTTGAGCTGAAATGATGAACCAATGACGAGAGAGTCCAGAAG |
| Pbr020171.1 | TACAATTTTTTTTTCTCTTGTATCATCCTTTGATTTGTCAAGATTCTTTTTGTAATATTGATATGTTTTATATAACTAAATCACTTATAGTACTATTTAGTTTATATATATATATGATTTTTATATGAATATTGCACTACAAATTTACATGTAGGGGAAAAAGATCGAAGAAACATTATCTCCAAGGGATTTATATATAGATTTTTTTTTTCTTCTGCTTTCTTAATTAAACTGGTAGCTAGGCATGCCTTCTTCAATTCATGATATGTGGAAAAAGCTATTTGTAGTTGCCGATAAGTGTGAGATGGTCCCCCTTCAAAATAATCCAACATCTATACCTAGAGAACCTATTGCATAATATTATACGGTAAACTGCGACCAAACTTATTCATTAATTAAGTGGTGATTGTTCAGTCTGTGGTATGAACAAATTGATGAAATTTTTATACTGTGGCTTGACAGATGGACGAGAAAGTATAGGTATCGTGAACACAAGTTATGTAATGTATAAATTTATGTGTTATAATAAGAGAGGACAATGGAGTTGATATGTCCTATATTTAGGACATTAGTAAAAACAAATTCACATGTTATATTATATAATTTGAGTAGAATGATGATAACGCTATGTTGTCTTGTTTCAGATACATAAAAGATTGCTCTAAATTAACGTGTTAGCATATTCAGATGCCACGTGGCCATAAATGTGCCATACCTTTTCCTTTTTCATTCCCATGCAATATGGTGTAGTAAATTCTAGTTAGCATCTTTTCTTTACCAAATGGTCGATTATTATTTCTGAAAGTTTATATTCTCTCTATCTCTCTCTGCAGCTAAGAGCCTCTAAGATGTTGAATTTGACAGGCAAATGAAGTACACAGCTTGGTAAGAAATTCAAGAGAGAGCATCATATATACTTTAGCAAGAGGTTTGAAATCGCACTGTTGCATCCTGTTGGTCTGTCCTACTTCTTCTGTTTCTTTTTAATAAGTTTTCGACATCCAAGAACTAAATCTCAGCTTCTACGACCTCGGTTTTCAATCTGATGTGAGTACAGTTCATCTTATGATCCTAATTATATCTTTCAAAATAGATTGATAAGAGGTAAAGGTAATTTAGGGTTTCCGGTAGTTTGAATTTGTATGATTTTGTGAGAGTAGATCAAGAAAAAAACCTCATTGCAAACTCTGATTTTCTACTAGCTTTTGCTCATCCTGTTAACTCTCAATTTTAAACAATTTATAAGTTCTTTAACTTCTTTGACGATTAAAATGGTTCAACTCATTAATCATATATATATGTGTGTATATATTTATCTTCTATAGTAGTTTGTGACCATTCTTTTTAATGCAACATCTGCTCAACTTCTCAGCACAATTTTCAGATTTGCATATGGAGGATCTCCTTAATAATTAGGGGAGCATATATATATATATGCAAATAAATACTAATAATGGAAACTTTGATGAGAAAATGTTTTCTTTCTCATCTATCGATCAGGAACTGAACAGTGATTAATTTAATTAGTTCATGAATCAATTTTCCATAAGATATAAACCCATATGGATTCCAAAAGAAATTAAGAATAATCATATGAATTCATGTAGTATATAGCAGTTTATTATTTTTCCCCTTTAATTTTTATTCTTGTTTTTTTTTTCAGTTGGATCTTTGGAGTCGAGTGATCGGAGAATTTCTTTCCATATCCGCAAAAAAGGAGCACTTGAAAGAAGGGCGAGATTACTATAATCAGAAATACTACA |
| Pbr007197.1 | TATACCTAGAGAGCCTATTAATAATCCAATATCTATACCTAGAGAGCCTATTGCATAATTATACACGGTAAACTAAGACCAAACTTGTTCATTAATTAAGCAGTGATTGTTCAGTCTGTGGTATGAACAAATTGATGAAATTTTTACTGTTGTTTGACAGATTGACAAGTTATAGCGGCATTGGAAATATAAGTCATGTAATGTGCAATTTTATGTGTTATAATAAGAGGGAACAATAAAGTTGATATGTCTTATTTTTAGGACATTAGTAAAAACAAATTCATGTGTTTTTTTTTTTTTTTTTTTTTTTGTGAGTATAACAATATATTTTACACCCAAGGATATGAAGTTTGGCTAAGTCACACAATGAAAAACTTAATTTAGTATCGAATTCATTATTTACAAGATTCGAACCGAATATCTATCACTTACAAGTGAACAAGAATATCTCCAAAATGTAGTATTGAGCAGCACAAATTCACATGTTATATTATATAATTGAGTAAAATGATGATAACGCTACGTTGTTATGTTTCAAATACATAAAAGATAGCTCTAAACTAACTTATCAGCGTATTCGTATACCACGTGGCCATAAATGGTGCCATACCTTTTCCTTTTTCATTCCCATGCAAGCGGTAAATTCTAGTTAGCATATTTTCTTTACCAAATTGTGAATTATTATTTTTAAAAGTTTATATTCTCTCTCTCTCTCTCTCTCTCTCTCACTGAAACTAAGAGCCTTTAAGATGTTGAATTTGACAGGCAAATGAGGTACACAGCTTAGTAAGAAATTCAAGAGAGCATCATATATACTTTAGCAAGAGGTTCAAATTGCACTGTTGCGTCCTGTTGCTCTCTCCTACTTCTTCTGCTTCTTGCTCATAAGTTTTGGACATCCAAGAACAAACTCTGATCTCAGCTTCTACAACCTCAATTTTCAGTCTGATGTGAGTACAGTTCATCTTATAATCCTAATTATATCTTCAAATTGGTTTGCTAAGAGATGAAGGTAATTTAGGGTTTCTGGTAGTTTGAATTTGTATGGCTTTTGTTACAAAATCAAAGGAAGAGAATAGAAGAAAAAACCTTATTTCAATTAAACTCTTATTTTCTGCTAGCTTTTCCTCAACATGCATGTTAACTTCTTGAATTTAGTTTCCAAATTAAATGTTCAATTTTAAACGATTCAGATTTCAATTCTTCAACTTATTTCAAGATTAAAATGGTCCAATTCATTAATCACACACACACACACACATATATATATATATATATATATCTTTAAGTAGTTTGTGACCATTCATTTTAATGCAATATTTATCAATATCTGCTCAACTTCTCAGCAGAATTTTCAGATTTGCATATGGAGGATCTCCATAATAATTAGAGGAGAATGTATATAAAATTGAAAAGAAATACTACTATCAGAATCTTTGATGAGAAATGTTTTCTTTCTCATCTATCAATCTCGAACCGATCAGTGATCAATTTAATTAGTTCATGAAGCAATTTTCCATAAGATATAAACTTATATGGAGTCCAAAAGAAATTAAGAATAATCTCACGAAAATGGAAGAAGGCTCATATGGTATGTAACAGTTTATTTTTCCTCTTAATTTGTGTTCTTCTTTCTCTTTCAGTTGAATCTTTGGAGCTGAACGATCGCAGTATTTCTTTCCATATCCACAAAAAAGGAGCGATTGAAAGAATGGCGAGTTTACTATAATCAGAAATATTAAC |
| Pbr039609.1 | TATAAAGTGGAGAGAACGGCGCTGTGTGCTGTCTTTTCCCGATAAGCCCGGTGGTTTGCTCAAATGGGGAAGAAGCAAACAACTCTCTCTCTCCTCTTCCATCTCTCTCCTCTTCTTCTTTCACTGCTCACTTCTTCTGCTACTTAACCCTCTCAAGCACCACTTTCCATTTTTCAGTTTTGAAACCTCTCCCCTCCGCTTTCCATCAATTTCCCACATTTTCTCTGATTATTTTCTTACCCGCCAAACATCAACCCTAACTCGCTCCCACCTCCCGACCCCTCTCGAATTCTCAGCTCACAAACACATATCCAGCTTCTGTGAGTCTGTTTCTCACTCAATTTTTCAACTTTCTGCAGATAAAGCTCAAATCTGTTTACTTTTTGTTTCTGGGTTCTTTGGTTAGGCCAGTTAGTGGTGCTTTTCTGGTTTCAGGAAGTCTGGATTTGTATGCCAAGGGTAGTCCTTGCAACTTGGGAGGTTCTTAGGTCATCTCATCAGTCTAATTTTCTTGATTCAGCTTAAATTTTGCATTTTTAATTGTAATTAGTGTTAATTAGGTTGTCATTAATATGGGGTTTGAGATTGGAGGCAATGGGAGGGATAAAGTATCAACATTTTGTCTTGATTTGACGGATTTTTTGCAGCAAATGAACTGATCCCATCAAGTTTAAATGCGCAGATCTGTGAGCTTGCGTGCTCCAAATCTCAAACTGCTCATGAATCTGCAGACAAAGAATCACACTGCTCCATTTCTGCTGTCTTTACATTTGTTTAAGTTTTAACAATCAGAGAGAGCTTGACATCTGTCTGTCCCAATCAAAACGCACAACATTATTCAAGGTTTTAATTTTCCCATTTTTTATTTAAGCTACCAATTATCATTTCTTCTAGTTTTTAATTGTTTAATATATATAGAGGAGAGCAAGAACATGCCTGGCATTTTTAGGGCACAGGAAAAAAATTGTGTGAAATTTACTGCTGCTGCTTGAATCTAAAGCAGAAAAGGGATTACATACTAATTATACTTTTTTTGGTTTCTAAATTGGTTATATTGAAAAGTTCATCTTCTTTAAAAACCCTTTTGTTAGGTTTAGGCATTTAACTTTTATTAGCTTGAGATCTCTGAAAGCTGGTTTTTTTAGTGTAAATATGTACTGTATTTAGGCATCTGATCAGCTGTTTTTACTTGCTTTTGTGTTATGTGTATTTTGGGAAAATTTTCTTCGTTTTTCTTTTCATTTATTGAATTAAAAATGGAAATGAAATATCAAAATTACTGCTGGTGGTTTTTTGCAGAGCTGAGAGACTGTGAAGAGTAAAACTGTGCAGGCTTTGGTTTGCTGCTTTTGAAAGCACACTGCGGGAACTGCAAAAAGCTTGAGAGAGAGAGAGAGAGAGAGAGAGTAGAGAGAGAGCCTCTCTCTCCTGACTTAGACCGTGATATATCAGCAGCGTGAAAAAGGCCTTTTTATCACTACACAGCCAAAGTACCCTCTGAGTTATCGTCATCAATGCATAGGACACTGCAACCCCACCTCCTCCTGCACTCCTCTTCCCCTTCTTTCTCTCTCTAAAACCGCTCTCTCCCTCCCCCAAATTCAACCCCTTTCTCCCTCTCTCTCTCCTCCCCTTTCTCTCTCTAGAAAGAGCTAAGTGGTGAAAAGGATAAGAAGCCAGCAAAGCAGAACAGCACAAGTGCCCAAATTGAATGAGTCTTTATGTGAGAAAGGCATGAAACTACTGCT |
| Pbr031206.1 | AGCCATGGATAAAGTAATCTTCCAAAATATTTTCCCTCGTCTGCCAACGTCTGTTGATCTTCGATGCACGATCGGTACATGAACCGCCACATAGTTTTTGGCTTCCTTCATAAGGCATATCCACTGCTAAAACAACTTGGGTGTTGTGTGCCTCTCCCCTCATTATTCTTCACCACGCCTCCGTCTTCATTCTTCATTTTGTTGTCCGGTTCTCTTTAACAACATGCTAAAGTAAGACATTGAAGATAACACTAATTATAAAGGATAGAGATGATTGTTATGAGTTGATGAAATTGGTATCCTGGTATTTATGGCAATTTGGAGATAAGGCTAAATAAGAAGAAGACACATTGTGTGTCGAAAGCAACTAAAATCTACAGCCCCACTGAGCGATGAGAGCAACTGAAATTTATTCGAAAATCTCAACAAGATTTTTGATAAGTATGGACACGTACGTAACGTAATCATAGCAACCTATTCTAAATTAATCTCATCCATTGAATAATTATTAAATAATTTGATTCATACATGAAAAAATATATAAATCATTGTAAATAATAACTGCCAATAAGTGGAGAATGGTAAAAAGAAATCAAAGCAATTCACTATATAAGTGAATAGTAATTATCCCAATTCGACTGTAGGCTCAAATCCACCAAGAAAAGGTAAAATGCTGTGATAGCCATGTGACAAATCCTGGGAGTGCAAAAATGCATAAAAAGGTACTCAGAAAGTGCAGTCGAAGGAAGCACGTTTGTCGTTTATTTTAGTGTAACTAAGAATACATACATACATATTTGTGGTACAGCTCTTACAAAATAAAGATGGTAAAGGAACCAGAAAAGCTGTTAGATATCATTATCAACACCTTGAAAAGGTGTGTGTGAAAGAGGTAGCCTCCCTCGAGAGTCACCTCTCTATCAAACCATCACTAATCATGTATAGATCCCTAGCTGATTACCCACTCTTATAATAAGTGCTTAAAAAGTTACTCCTAAAACGTAGGAGAGAGGGCTAGAGTTACTTCCTCCTCTAAGCGTTTTATGGTAGATTTACAAAATTTTATACTTGCGGTCGAAACTATTCATATTATATAACTTAATAAGTAAAAATAATCTGTAAAGAAAAATTAATAAAATATAAAATTATTTGGTCATCAAATTATATAAACAAAAATATTACGTATCGTATGTCTGATTTGCTACAATTTACCATATGGCAATAGCCTACAACTTGGGTTGTTGCTTAAATTTTCCATTTTAATTCCTTTTTTTTTTCTTTTTCTTTCTCTTTAAATTCGTAGTTTCGTGGCCACATAGAAAAACTCACCCATGAATTATCAGTAGTCTCTGTCTGCACACGCACTGTACCTTTTTATGGACCAATAAGTAGTTACCTTATCTATAAACTTGCAAGAATACAAGTGGAGTCAAATTTCTGTGCCTGTGAAGTGTGGCTTTTGGTAGCGTTTATTGGGGTTTGGAATCTGGATGGAGATTGCAATGCAGAGTTTTGGGGATCGGCGGCAGCAGCACAAGTATCAGAACTGTGACAGCAACGGCCACAGCTAGAGTAAAAGCAACGCAACCAATACAATTTGCAGAAAATAATGCAAAACAAAAAGAGAGAATAATTACA |
| Pbr022498.1 | GGATGATGTTGAATTTCGGAGTTGATCCGTTCACGAAAGTTGTAAAGCTTGCATTACGAGCGATTGCATAATTTTTTTTTTTTTTATTTTTTTACATTTTTCACACTTCTACATAAATTATTATGAATTTTTATTACTAGTGCACTCAGACAAAAAACACTATTCATGAGGGTTTTGAAAATCTAAATGACGAAGTAACCATCTTAAGCGTAGGTGATGCAATCATGAGCGTTTATATATATTTTTTTTCTTCACCTTTTTCACACTTGTACATTAATTATTATGATTTTTTATTTATTTTGAACTCCCTCTAAAAACAATTTTCATGAGGGTTTGTAGGGTTTTAAAAACTCAAACGACGACGTACCACTCAAAGCGTAAAAGATGCGATCGCAAGTATTTGCATATTTTTTTCACATTTTTTATACTCGTAAATTAATTATTATGAATTTTTAATGTGCTTTTGTATTTTTAAATTGCTTTGTTACATTTAAAGTGCTGGAATTTTCAATCTCACTTATGTGAGATTAAATACTATGGGTATTATATTAACTAGTAAGTTTTTTTAGTAATATAAATTATCAAACTAATTTTCTTAACTGTTGAAACGGGTTACCTGCAGGTAAATCTGTTAAGAACAGATTCTTAACAGATGACTCAATAACGATCCCAATTAATTAACGTGTTAACTCAAAACCTGTTATGTCGTGTGTTTTCGTGTCGGATTATCAAATCATAAAAGAAATTGTGAAGTCTAAAATGACATAATCTTACAACCTATTCTAAATTAATTTCAATAATTGTTAAACAATTGAATCATCCACCAAAAATGGTGAAATGTTACGACAGCCATGTACAAATCTGGGGAGTGCAAAATGCGTATAAAGTACTGAGGAAGTGGAAGCCGAAGGACGCACGTCTGTCGTTTATTTTAGTTGTGAGAATACATATTTGTGTGGTTGAACTCTTGCAAAATAAAGATAGTAAAGGAACTAGAAAAGTTGTTGGATATCATTGTCAAAACCTTCAAAAGGTGTGCACGAAAGAGGTAGCCTCGACAGTCACCTCTCTATCAAACCATGACTCTAATCATAGATTCATAGCTAATTTACCTAAAACTCTTATAATAGCATGTGCTTGACAAGTTACTCCTAAGTAGCAAGAAAGATAGAGAGTTAGGACTACTCCAAGCATGAACTAGAATTTCTCTTAAAACGTTTTGTGGTCGGTTCAAGTTGTATAAACAAATATTACTAACTGTCTATCTATTTTTTTACAATTTATTGACTAAACAACATTTGTTTTGATTGATATTTTTGCATAAATAACTTTTACATAAGGATTTACAATATTAATAGTTTCGGTTATAAACACGAAGCTTTGTTAATCAATCACGTAACGCTTTGAGGAGAAATTTAAGTAACCAAACATTGTTATAAAGGAGTTGAGTTTGGTTGGGACCTGCTATTGTTATACGGGAAGAGATCTCATCCAAATCTCTCCTACCAAATTTTAAGGATCTGGAAATCCAAACCCTTAAAATTTAATCTAACGGTTACAATTATTATAATTTTTAGAGGTCTCCTGTTTGTAGCAGTTGGATTAAATTTTAAAAATTCCGATCTTTTAATCCTTAGAATTTGGCGGGAGAGATCTGGATGGGATCTCTTCCCTTGTTATACTGGTTAGTGGGGGTGAGATTTCTAATCTCCTCCTTTACACATTCATCAACACCCGTCTCTCTCTCTCTCTCTCTCTCTGTGTGGACCAAGCTAGGACTAGGAGTCACCGCCCCCTCTTCCTACATCTCAAATCTCATATTCCAGTTTCAATTTGCAGAATTTTGTTTTTCTACTTAGGACCCCTAAGTTCTCATGTGATTCTCTTTATTATTTCCCTTTTTCTTGATGGATATATTATATAATTTATACCCAATAAATACTAGCTACAACTCGGGTTGTTACTTAAATTTTCCATTTTAATTCATTTTCTGTTTTCATTCTCTTTAAATTCGGTAGGTCTGTGCCCACAGAAAAACTCACCCACCA |
| Pbr038238.1 | GAAATAATTTTTTTATATAAGTGATACAGAAATAAAAATTAAAAAGCTTTGAACAAGTCGGATTATAAAATTTATTAGATCAAAATATGTTATTTACAAAGAGAGAATACAGGTATTATCCAATTGTAAAAAAAAAAAGGGAGAGTTAAAAGGAATTGAAAGGAAGGGATGAGGATCCTCTTTATGAGGAGCCTAGGATTCTAATATCTTGACCGTTTATCGTATATTGTGTGGTCAGTTTTCGTTAGGTACTATTTGTGTTTAATTTTAAATAAAAAAATTCAAAATGATTTCTCGTTGCATAATGTACAATAAACGGTTATGATGTAAGAATTTGTAGAATCTTCACAAATTGAATTCGGATGGGATTCAAATATGAAAGAGGAATTAAATTTTGGAGCTGACTGATTGAAAGAAATGGAGAAAACCTAGAACAAAATAGTTTGATAATCTCCTACTTAAACGATTACGGTTAAGTTATATCAATATTTTATATTTTTTTATAAAAACAATAAAATTAAAAATAAAATAAAATATAAAAGGAGACGAAAGAAAGGAATGAAAATAAAAAGGAGGAGAATCTTACTTTTTTTTTTTTTTTTTTTTTTTTTTCNNNNNNNNNNNNNNNNNNNNNNNNNNNNNNNNNNNNNNNNNNNNNNNNNNNNNNNNNNNNNNNNNNNNNNNNNNNNNNNNNNNNNNNNNNNNNNCCCTTTCCGTGTATTTTGTATAGTTGAAGTGTTGTGAAGGAGGTGCAGATATATGGTACAATGAAAGCAGAGAGAGAGAGAGCAGACGCAATGATGAGATCACAGAGATCTTTAATCCTCTTTCTGACCAAACACTGCCACCCTTACCACTGCCATCATCAACTCCACCTGTACATATAGGAGGAGAAAACAATTTAAAGTGTGTAATTTTTCTCTCCTCTTCCACCTCTCCTCCCTTTAATTCCCCCAATTTCTCACTTTATTTCTCTAGAAATTGGGATTTAGTTTTCTTGTTGTTGGGTTTGCCTGCTCTGCTGGGTAGTGTTAACCCCTATATATAACCAGTTCTGGTAATTAAGTTAGTCAGTCCACATAATAAAGCTGAAGAAGTGTACAATTAAGAGGCACAAATACCAAAAGGAGAGAGAGAGAGAGAGAGAGAGAGAGAGAGATGGGTTAGGCTGGCAGGGGAGCTGGTTTAGCCTTTCTCTTTCAGTCTGGTTTGGGTTTAGATTATGGTGGTAGGGGTGGGGGTGTTCGTGATATGGAACTGAAAACATTGACTGACAGAGGCTTTTGGGAGAGCCAGAAGCAGGAAAAGCAGAAGCAGCTTCAGGGCTCAGAGGAGAGGTGGCAGCACAAGCATCAGGAGATCCCAGCGGCAAAGAAAAACCACTAGCAAAAAGAAGTCTATTTGTAAAAGTAATTGTTGTAGAAGCAGAAGCAGAAGCAGGTGTACTGTTCCATTAAAAACTGGAAGAGGAAAATCACAAGGGTCTGACTCTGGATCCTCAAAGGAATCACCACCATTATCTCCACCAAAACCCAATTCTCAATTTTAACCATCTTGTCCAAGGAGAAGAAGAAACCCAGCAACAAACCCAGCAGAATCACCACCAAGCTTTCAACTTTCCGTATTTCCAGCAAGACCCACAAACCCAGCACCAGCACCAGCATTTCTTTGGAGAAACCCAGCAGTTGCAGCTGCAAGAGCAGCAGCAAGCTCCAAAGAAACGCAGCTTCACTTCTTCTTCTTCTTCAACTTTTGGGGAACAGAGCATCGAATATGCAAGATCAAAA |
| Pbr025856.1 | TGTTCCAAGGTGTGTATAAGTCTATCGATCAAGATGAAAGCAAAACATAGTAAGGCTCATATCTTTGTGAGAGGTGTCTTAAAAATTAGGGTAATTAGCTGGCATGATCAATTTTTGACATTTGGTTGCAGAAATGATCATTTTTTAGTTATGCCTACATATCGACGACATAACAATCACACAACGATGACATAATGACAATGTGTAAAAACTAATCATTGTTTCAACCAACTCTTAAAAACTGAACATTCCAATTAATTACTGTAAAAACTATGTTATTCGCCCCAACTAAAGTGTTTTCCTTCGTAGAGAAGTGTTTTTTGTTTATACAATGACATTCTATTCTAAATGGGGGTATTTGCCTAACCTACATAATACATCAACCATAACGCTTAGGTTTCGAATACGCTATTTATGTGAGTCAAATTTGGACAATTGTACTTCCACATGAATGAAAAATATTAATATACCATAGGAGAGTTAAAGAAAAGGGTAAATAAGGGAAGGAGTCAAATTTTGGAGCTGACTGATTGAAATAAATGGAGAAAACCCAAAACAATAAAGTTTGGTCTTTTTCTTTCCTGTATTTTGTGTAGTTGTATGTAGAGAGTGAAACTAGTAGTAGTGTGGTGGAGGAGGTGTAGATCTACAGTAGAGAGAGAAGACGCAATGATGGGTTCACATAGATCTTTAATCCTCTTTCTGACCAACACTGCCACCCTTACCACTGCCATCATAAACTCCCCCTATACCTGTAGGAGGATACCAATTAAACTGTATATTTCACTCCTCTTCTCCCTTTAATTCCCCCCAATTTTCCACTTTCTCTCTCTAGAATTTGGGATTTGGTCTTCTTGTTGTTGGGTTTGCCTGCTCTGCTGGGTATTATTAACCCCCATAACCAGTTTTGGTAATTAGGTCAAGTCAGTCAGTCCACATAATAGAGCTGAAGAAGCAAGTGCACAATTAAGAGGCACAAATACCAAAAGTAGAGAGAGAGAGAGAGAGAGAGAGAGAGAGAGAGAGAGATGGGTTAGGCTGGCAAGGTAGCCGGTTTAGCCTCTCTCTTTTAGTCTGGTTTGGGTTTTAATTACGGGAGGACCCTTTTGGAAAAAATTGGTGGTGGTGGTGGGGTAGTTTGTGATATGGAACTGAAAACATTGAATGGCAGAGCCTTTTCGGAGAGCCAGAAGCAGAAGAAGCCGAACCAGGAAAAGCAGAAGCAGTTTCAGGGCTCAGAGGGGAGGTGGCAGCACAAGCATCAGGAGATCACACAAGCAAAGACAGACCACTAGCAAAAAAAAAAAGTCTATTAGCAAAAGTACTTGTTTGTAGAAGCAGAAGCAGTTGTACTGTTCCATTAAAAACTGGAAGAGGAAAATCACAAGGGTCTGACTCTGGATCCTCAAAGGAATCACCACCACCATTATCTCCACCAAAACCCAATTCTCAATTTTACCCATCTTGTCCAAGAAGAAGAAGAAGAAACCCAGCAACAAACCCAGCAGAATCACCACCAAGCTTTCAACTTTCCGTATCTCCACCAAGACCCACATACCCAGCAGGAGCACCAGCATTTCTTTGGAGAAACCCAGCAGCAAACCCAGCAGAGAGCTCCAAAGAAACGCAGCTGTACTTCTTCTTCTTCTTCAACTTTGGTGGAACAGAGCATCGAATATGCAAGATCAAAA |
| Pbr000450.1（10） | ATAGACATGAGTTTCAGAAAATAGAACTGAAAGAAATTGACTGAGGCCTCCTCTCTCCTTCCTTCCCCAATGCTACTTTTAAACCAATTCTTGCTGCATCATTTTCTTCTCCTTAAATCACCCACTAATAACCATTTAGTGATGACAATAAGTGAGAGGAAATGTAAAACAATTGTAACTCCTTGGGTAGCCTTTATGTCAATTACTCCCTCTTAATCCTCATCTTTAATTCCATTTCCTCTGATATTTGAATAGGTGTCAGCTGGTTTGTTCTTGATTGCGTCAGTTTTGGCTACTAGATTTATTGGATTTATTGCTTTCAATGTTTTTTTTGTCAGTTACAAACTGCTCAACCTCTTGGGAACCTTTCAGTGTTAAAGCGGCCATAACTTCTTCTAGAAAAATGATATTAACAATCCGCGAAATGCTCCAGAAAATAGACATCCGTAGCTTTCCAAAAATATAAGGCTTATTCTCTAATTCATTTTGAGTTGTCGGCAACTTGCTTCCAAAGTCAGCTGACCTGCACAGACAGTTTTGACGAATTTGTTACTTAAAATTCCACTTGTGTTATTTTTCTTTTCTTTACTTGACAAATCCTATAAAACACAAAAACAAAATAAATAACTCAAAAATATAAAGAACTAACTAAGAAAAGATAAGTGAATTTGATATAAAAAATATATAAATATAAGCTTATCACATATGCTAATATGGTGCAATATATTGATCTTAGTAGTTAGGGTTCACATCTTCTGGTGACTCACACTCTTGTATTGCACCGATATTGTCATCAACTTCACTACTTAGGTTTTGTAGATGACGTGATTAGGTAAATAATGGATATAAAATTTTATAGCAAAAGGCTTCAGTTCAAGGAGAACTAGCAACTATTTACATACTTTTTACGTATAATGATCTCTTCTTTGACGTGAGATAATTTGTCGTTCATCAATATTTCCTAACTCTAGTCTAGAGTTTAGAGGGTCCCATCGAAAAGGTTATATGTCTGGTTGACTAGCTTTTGAGGGCATTAGTTGCTTGCTTAATAAGCAAATGAGACATGAACTCCTCCCAAGAAGCAACCAGACCAAACCAACAATATGATTGACAGTCACTGTCCCTTCCGTTTGTGGAATCTTTAGTAAATGTTGTCTATCTTTACCAAGGTTTTACTTGTGACCAGCTTTCTGAAAATCCACCAAGAAAAAAACAGTGACTCGAAGTGGATAGTTGCAGCAGAAAAGGGTTAATTAATTAAGAGCTGTGACATTTGCATCAAGTCAGCAAGTAACCAGAACTAGGGTTGGCGCCATATTCCTGTTTCATACAAGAGTTTGCAGCTGAACAATAAAGAAGCGATTAGAAACCCTACTTTTCTGCATTAATGCTATGCTTAGCTTTTAACAGGCGATCTTTATGACGTCTTTTGCTTCCTCAGACTGTGTCATGCCATCTCTATTAAACGCATCTCTCATTTCGTAAATTTTGGAGTACATGGATATTAATATATTGTATGTTGTGTGATGAGTTTAACGACTGGATATGCACTAAAGACTTCGGGAAATATCAACAGATGAATCTGAGCAACTCAAAAGACCGTGTATAATTTACATTGATGTATAGTAGGACTCTTCATACCTATATTGCTGGACTATTTGCACTACTGTCTTCCATGAGAGAGAGAGAGAGAGAGAGAGAGAGAATGTTACGACAAAGAGAATCAAAGAATAAGGAAGAACAGAATGAAGATTATCAGCATGTTCACTACTTG |
| Pbr016172.1 | TTTCAAAAGTCTGAATTGTTTGATGAAGAAAATTTGATGGAAGGAATCTCGAGAATCCTTTCCACAATGCTGCACGTAAAGAGCAATAATATCAAGTGGTCTCCTCCCGCATGCAATATACCGTCTAAAGTTGCAGCAAGACAGTAGTACCGATACATTGTACCCAACTTCTTTTTCTTTACTCCTCTGTCGCAACTCAGCTGCGCATGCCGTCCTTTTGGTACAAACTAATTTCAGTGCTGACTGTTGTAGTCCTCGATCACAAGAGCCTCGGTTGAATACATTATCTCATACAGAACAAGCATTATACCTTCCAACCTTCCGTGTATATGCTATTATGGTGCAATACATATTGATCTTAGCAATTAGGGCTCACATCTTCTGGCGATTCCTTGATTGCACTGATATTGTTATCAATTTTACTACTTAGGGTCATAAATGGTGTGATTAGGTAAATAATCAGCGTGAATTTTATGACGAATAAAATTCAATTTAGAGAGTTTGCTAACATTTATATACATTTTACTCATAGTGATATTTTTTCGATATAAGATAATTTGCATTTGATCGATAGTTTCTAAACTCTAACTAAGAGAAATTCTTCAGTCCGGAGACCAAACCAAGTGGCTCATACGGGCCCACACACAAAAATCATCCGTTATCCCACTTCCTTATTTTTTCATGTTTACCTGGCTCTATTTGTGTCCCACCTCCACCTCACACAACACCAATGACCCATCATCAACAGTGGTCTCAGACAAGAACCTCTCCTAATCTAGAGTTTAGAGGGTCCCATCGAAAAGGTGATGTGTCTAGTTGACTAGCTTTTGAGGGCAAGAGTTGCTTGCTTAACAAGCAAATGGAGACATGAACTCCTCCCAAGAAGAAACCAGACCAAACCAACACATGATTGACAGTAGTGTCCCTTTTGTTTGTGGAATTTTTACTATCTTGTCTATCTTTACCACTGTAGGGTTTTACTTGTGACCAGCTTTCTGAAAATCCACCAAGAAAAAAACAATCTGACTCAAACTCGATAGTTAATTGCAGCATATCAGGTTAATTAAGAGGTGTGACATTTGCATGATCAAGTCAGCAAGTAATTAGAACTAGAGTTGGCGCCAATATTCCTGTTTCATGCAGGAGTTTGGAGCTGAACAAGTAGGAAACAATTTAGAAACCCTACTTTTCTGCATTAATGTTATGCTTAGCTTTTAAAAGGCTATCTTTATGATCTCTTTTACTTCCTCAGACTGTGTCATGCCTTCTTTATCAAACATATCTCTCGTTTCATAGCTCGAAAATTTAGAGTACGTAAATATTGCATGGTGTGTGATGAGTTTAACAGTTAGATGTGCGCTGAAAGATCCGAGAGAATTGAATGGATGAATCTGAGCAACTTTGAAAGCACTGTATAATGTACATGTATATATAGGACTTTTCACCCCTATATCGCTGGACTATTTGCACTAGTGTCTTCCATAAGAGAGAGAGAGAGAGAGAGAGAGAGAGAGAGAGAGAGAGAGAGAAAGTTACAACAAAGAGAATCAAAGAATGCGAAAGAAGAGAATGAAGATTATCACCATGTTCAAGACTTG |
| Pbr013244.1 | GTTTCTAAATTGCATCAATTTAACCTCTTTGTAACTTTAATCGTTTAATTAGACGTTAAGTGCTTACGTGACATAGATTGGGCTCACATTTTAAGCACACGTTAATATTTTTTTATTTTTTTGAGTACATCGATATTTTTACACTAAGAGGAGAAGTTTGGCAAAGCCACACAACGGACAACCTAATTTGGTATCGAATTCGCCATCTACGATATTCAAATTTAAGACCTCTCATTTCCAAGTAATGAGGAATACCATCAGACCATAGTACTGAATGACAAGCACACGTTAATATTAATGTCTAAAAAGAATTGAAAGAGTGAAGTGCTAAACATTCAAATTGACAGTCAACTAGTACTTAAAATGTGGGTCATGTCTATACTATTTCAGCACTAATTGTCTAATTAGACAATGAAATTGATGAAGATGCTAAATTAGTGCAATTAAAAAACTAAGTGGTGTAAAACACATAAATTGAAACCTCGTGATTCATTTGACAAATAATCTCCAAACATTATGGTGTAAAATGCAATTAACCCTATTGTTTTAACATTAAAAGAACATTGTATATAGATTTGGTTGTTAAAACATTGCATATAGATGTTAAATCGAGTCAGATAATGATAGGGACATTTGGTAAGCCAGTCAAGGCAAGAGTTGCCTTCCATGGTGGTAATTAAAAGAAGCTGGCTCTAAATATGGTACAATACCTGTAGCCTATTAGCCTATAGCCTCTCCTCTCTGTGTTTACTGTTACATTCATCAGAATTGTCCTCTTCTCCATCTATCTCTCTCTTTTTCTCTCTTTCTAGGGCACCTATACGTAGCCCTCTTAAAATTCGCATAGATTTATGAATAAGTTTTTCATGAATATCAAAATCATGAAAACAACTTTCATACAACGTATTTAACAACTTCTATGCCACGGGATTAAAAGTTAATTTCTTTTTATATCTTTCTCATCGAATACCATCCAATTATATAGACACAAACACAGAGGATTCTGAAAAAGGATGGCTAGGTTAGGTCGCTTTCTCGCTCCCACCCCTGCACTCACCCACGTGATGTGTCACCGTTCACTTACATGTTCAAATTAAGACTCAAGAGATATCATCAGTTGCACTACTAGCTAGATGTCTACCTTATATGTATGTGTCTGTGTGTATATATAGCTTTACCTAGCTATCCTCTGTCCCTCGTCTTCTTTCCGTAATTCGTTTCCACTCACTCACTTTCTTTCTTTCTTTCTCAACCCAGTGCTTCTACTGTTGAGGAGGTCACCATTTGTTGGTTGAATATGTAATAATTAACGGTGAAGCAAATACCAGTTTCCAGGTCTTGTTTTAGCTCTAAACCAACAACAAAACACCACCATCGCTCTCATCAGGTTGTGCGCCTTGTGTTTTTCTCTATGGCTTTTCATATCTAGCTGCGTCATTTCCTTTTAGGCTCTCTTGATATACATGTGGAAGAACAGTAACCCTCCAGGGCTTCCGCACAGTAGTCGATTTATTTCTTGCCCTTTCTCTTTACTACTACTGTTTGTGTTGTTTTTCCGGCGGTCAAAGCAGCACCGAATTAAATGAGACTTGTCATGCACCATCACAATCTTTAGGGTTTTGTGGAGCTAAAGAGTTGTCATATCCAATTATATCC |
| Pbr030633.1 | TGTAGTTTGTAAGTCTCAATTCATAGATCATCCTTACAAAAATTAAATTCAATCCAAAATTTTTTGCCTATTTAATTATAAAGATAAAATTTCATTGTTTCTTATATAACAAAGTATTCGTTAATTTTTTTTTTTTAAACCCAACTAGATGTCTTAAATATTTCTGATTTGGTTAATATTTTGGAAGAATGATCTATAAGGTGCAATTTGAAAAATAAATAGTTCAGATTGTTAAAATTCGATGTCGTGTGGACCCTACAACTAATCTCCATTTTTATTAAAAAAAAATAAAAATAAGAATCATTTCCCTTAAAAGATTCATCTGTTTGTAATGGTCATAATGAATTAGGCTCCCTTTTAAGTACGGACCCAAGAGTATATGTTAGATTAATTTGTCAACAATGGAGAAACTTTTGTGTATACACGATTTTTGTGATACGTAATGAATTAGAGAGAATACGTGTTATATTAACACCAATAAAAATTAACTTATTAAAATTAAAACACCATTTCATAAAAAGCTTACGCCAAAATTGAAGTTTACTTTTTAATTTTCAAATTCTACAAGAGGCATTATCTAGTTGGGGAATGATTTATATAGTCTTTTTTTTTTTCTCTCTTACTTTTTTTTTATAAGAATTTTTTATTCCTCTTACACATTCCTTTATTTTGACTTTTAAATTGAATAAATAAATAAAAATTAAGTCACAAAAATTATCTGAACCATGTAGGAAGAGAAAAAATGTGCTTGGAAATTATTATCTAGGCTGGGGTATTGCCTAACCTAACCCTAGTTAATTAAACATGGTCTTGCTCTTTTCTGGAAATTTTAACGGGTCTAAAAGTTGGGTTGTCAAATTTCCCATCAGTTTTGGCTCGGCATGGTCTTTCCACATTAAGTGGCACAAACCCACATATTTTGCGCCTTACAAGTCCGGTGACACATCACCCTCAATGAAACCCAAAATTTGGAATCCGGATTCTTTTTGTGAAGATTCCGAAAATTTATAAATTGTGTTTGTTCATCGTATATTATGCAATTAGTTTTTCTCATGTACTGTTTGTATTCAATTTTAAATAATAAAATTTTAAATAATTTTTTATCATACGATATACGATAGATGGACAAAATACGTAGATTCTCGGGATCCTCATCAAGAAGATCCGTAGAGGATCCTTATTCCAAATATTGTAGCTTCAAGACTCTGCCCAAGAAACAGTACACCGGAGGAAAAAAAAAAAAACAAAAAAAAACAAGATAAAAGAAGAACAACAGAAACAACCAAAAACCCATCTTTCAGATGTCTCCAACGCAAACAAAACAGTCCCCCACTTATTAGTGCTTCCGTGTTTATATATCCGATACTATGCATCTTTCACAGCAGAGCTCACTCCCCTTGATCTTCTTTTTAAGAACACAGATGGATCTGAAGTTGCAGTGAGAAAGTGAGTCTGAAAGAGAAGTCTTGAGAAGTTCAATGGGTATGAAGAAGCTCTCTTTGCCTGTTCGAAATTAGGATAATTTCTTTCTAGTGTTCAAAAGAAGAAGAGGCTCTCTTAACAAAGGTATTATATTGTAGAAGGACTGCCTGACGGCCTGAGGAAAAAGAAATC |
| Pbr027488.1 | GTTGCCCCTTTTACAAGCTCCATACCGAAACTCCTCCCTCCTGCCTTCTATTTAAATTGTCTTCGTTTTGAAAAACTTCCAACAAAACGTATAGTAGACGCGCGTGGTGTTTGAGAATAATTTGAAGACCAACGAGGGATCATATCCATATATATGCATGTGTGATCGAAACTAGCTAGGATATTATCTCACTGGTATATTATTTAAGTGAAGTGTGGAAGCAGAAAGACCAATATTATTGTTTTAATATTGAAAAGAATATTGTGTATATATAGATGTTGAATCGAGTCAGAGAATGATAGGGACATTTGGTAAGCCAGTCAAAGCAAGCGTTGCCTTCCATGGTGGTAATCAAAAGCAGCTGGCCCTAAATATTGTACAAGACCTGTAGCCTATGGCCCTCTATAGCCTATAACCTCTCGTCTCCCTGTGTTACACTGTTACATTTATCATATAATTGTCCTCTCCTCCATCGATCTCTTTCTCTTCCTCTCTCACTCTTGCCTCACTCACCCTTTCCAGGGCACCTATACATGTAGGCGGTCAGTTTCTAGCCCTCTAAAAATTCCGTAGGGATATGAATAAGTTTTTCAAAGAATGTCATATAATCAAGAGAGTAGCTTATATAAAACGTAATTAACTACTTACACACATTACAGGATCATCGAATATTGATTGATTAATTACTACACACCTCTTCCTTTCTTTCTTTATTCTTTCTCATAAAAAACAAAATAATGAATTATATTCACACACAAACACAAAAGATTGGCACGATGAAAATGTTGCTCTTTTATAATCGAAAAGTCATGGATTTGAATTGTAGGAACATCCTCTTTACAAAACAAGAGTTAAGCTGCATACGATAAACGTTCTTCTGACCCTTATAAAGCAAATTACCTTATTAGCACACAAACACAGAGGGTTGTGAAAAAGGATAGCTAGGTTCGGTCGCTTTCTTGCTCTCACCCCTGCACTCACCCACGTGATGTGTCACCATTCACTTACATGTTCAAATTAGGACCCAAGAGATATCATCAGTTGCACGACTAGCTAGATGTCTATCTTATATATACATGTATATAGCTTTACCTAGCTATCTCACTTATGTGTACTCTTCTCTCCGTCCCTCGTGCTCTTTCTATATTTCGTATCCACTCACTCTCTTTCTTTCTTTCTTTCTTTTGCAACCCGGTACTACTACTGTGGAGTACGGTGACATTTTCTTGCTTGAATATGTAATAATGGTGAAGCAAAAACCAGTTTCCAGGTCTTGGTTCAGCTCTACACTAGCAACAAAGCACCGCCATCGCCCTCATCAGACTCTGCACTTTGTGTTTTCTTTATAGCTCTTCATAACTAGTTGCGTCCTTTTCCTTTAAGGCTCTCTTATACATGTAGAAGAACAGTAACCCTCCAGCCAGGGCATCCACATAGTAGTCGATTTATTTCTTGCCCTTTCTCTTTACTACTATTGTTTGTGTTGTTTTTCCGGCGGTCAAAACAGCACCGAATTAAAAGAGACGTCATGCATCATCACAATCTTTAGGGTTTTGTGGAGCTAAAGAATTGTCATATACAATTATATATCC |
| Pbr013717.1（15） | TGTTATATCTCTGCACTATCACATAGCTTAGTGTATAAAATGAGTACTGTCCCATGTAATAAGTGAACAGATGTGTTCCATATTAAAAGTGAAAGAATAGTAGCACACTTATCATCTAATTATATCTTCACATGTACCATCTTTTTAAGACCTACTCTATTTTTTAGAGTAAAACTTAAATTTAAGGTTCTAAATTCATCTGAACCTACTCCAACCCTTGGGGCAAATATGAGTTTTAACCTAAAACTCATTTCTGGGGCAAATTTAGGCCAGGATTCCCCTCGAGTTAGTGGGCTAGCCTCCCATATATGTGTATTTTTTTTAGTTTTATATTTATAATTTCATTGAATCTAACGGCTAAGATCTATAAGATCAAATCCAATGGTAAAATAAATAGATCTAGCAGTCGAAATTTAAATCCAACAGCCAAAGTAATAAAAAAAAAAATCTTTCAGGTCTTTCATATTTGTTCATAGTGTTCCACGAGTTTTATGGTGTCAGTTTAGTGATTTTTCAATATTTTGTCGGAATTTAAATATTTTAGGTTAAAATTTCATAAATTAAATTAGGATATAGTCTACATAATTTTTTTTAAGTTACTTTAAGAAAAAAAATTATTCTAAATTCATTCTTTAATAATCCCGTCCTAAAAATTTAACCCAGAAATGTTGGAGCAGAAAACTTATTTCTGAGTTAAAACCTAAATTTTCTAGGTTAAAAATTTTAGGTTTTAATCCAAGGGTTGAAGATTGTCTAATAGAGGTAAGACCCACAAACGCATGTGGATATCATCTCTATTAGAGAGATGATACAAATAGTGGTATAACTAGATGGTAAAAATAACAATTTTGAAATGGGGTGTGCTATCCACACACCTCTTTTTACTTTTCTTACACCCCTTGTTAATTTCTGTCATTTGATCTTCTTTAATCATCAAATCCAACCGCTAAAAATTAAAAGAATATGTGAGAAGTAAAAAATGGTGTGTTGATAACACATCATCTTTGAAGGTGAAAAGATGTGCATGGAAATTATTATCTAGGCTAAGGTGTTGCCTAACCTAACCCTAGTTAATTAATTCAGTATGGTCTCTCTTTTCTGGAAATTTTAACGGGTCTAAAAGTTGGGTTGTTAAAATTTCCCATCAGTCTTTGGTTAGGTTATGGTGTTTCCACAGTAAATGGCACAAACATTTTGCGCTTCCCATGTCCAGTAGCACATCACCCTCAAACTCAGTTAATAATTTTGTCCACACCATTGTACGTTCAAGATTTTTCCCTGAGGATAACTATACTACCCACGAAAGACTTCACCGGAAAAAACAAAAATGAGAGAAAAGAAAAAAAAACAGAGACAGCCAAAAACCCATCTTTCAGCTGTCTCCAACACAAACAATCCCCCACTCATTAGTGCTTTTGTGTCTATACCAGATGATACCATCTATCACTGTAAAGTTCACTCCCCTTGATCTTCTTTTTTAGAACACAGATGGATCTGCAGTTGCACTGAGAGAGTGAGTCTGAAAAGCGGAAGTCATGAGAAGTTTAATGAGTATGAAGAAGCTCTCTTAATTTGACCTGTTTGAAATTAGGATAATTTCTTTCCGGTGTTCAAAAAGAAGACGAGGCTCTCTTAACAAAGGTATTATATTGTATAAGGACTGCCTGAGGAAAAAGATATC |
| Pbr003924.1 | TTTTAGAGTAAAACTTAAATTTAAGGTTCTAAATTCATCTGAACCTACTCCAACCCTTGGGGCAAATATGAGTTTTAACCTAAAACTCATTTCTGGGGCAAATTTAGGCCAGGATTCCCCTCGAGTTAGTGGGCTAGCCTCCCATATATGTGTATTTTTTTTAGTTTTATATTTATAATTTCATTGAATCTAACGGCTAAGATCTATAAGATCAAATCCAATGGTAAAATAAATAGATCTAGCAGTCGAAATTTAAATCCAACAGCCAAAGTAATAAAAAAAAAAATCTTTCAGGTCTTTCATATTTGTTCATAGTGTTCCACGAGTTTTATGGTGTCAGTTTAGTGATTTTTCAATATTTTGTCGGAATTTAAATATTTTAGGTTAAAATTTCATAAATTAAATTAGGATATAGTCTACATAATTTTTTTTAAGTTACTTTAAGAAAAAAAATTATTCTAAATTCATTCTTTAATAATCCCGTCCTAAAAATTTAACCCAGAAATGTTGGAGCAGAAAACTTATTTCTGAGTTAAAACCTAAATTTTCTAGGTTAAAAATTTTAGGTTTTAATCCAAGGGTTGAAGATTGTCTAATAGAGGTAAGACCCACAAACGCATGTGGATATCATCTCTATTAGAGAGATGATACAAATAGTGGTATAACTAGATGGTAAAAATAACAATTTTGAAATGGGGTGTGCTATCCACACACCTCTTTTTACTTTTCTTACACCCCTTGTTAATTTCTGTCATTTGATCTTCTTTAATCATCAAATCCAACCGCTAAAAATTAAAAGAATATGTGAGAAGTAAAAAATGGTGTGTTGATAACACATCATCTTTGAAGGTGAAAAGATGTGCATGGAAATTATTATCTAGGCTAAGGTGTTGCCTAACCTAACCCTAGTTAATTAATTCAGTATGGTCTCTCTTTTCTGGAAATTTTAACGGGTCTAAAAGTTGGGTTGTTAAAATTTCCCATCAGTCTTTGGTTAGGTTATGGTGTTTCCACAGTAAATGGCACAAACATTTTGCGCTTCCCATGTCCAGTAGCACATCACCCTCAAACTCAGTTAATAATTTTGTCCACACCATTGTACGTTCAAGATTTTTCCCTGAGGATAACTATACTACCCACGAAAGACTTCACCGGAAAAAACAAAAATGAGAGAAAAGAAAAAAAAACAGAGACAGCCAAAAACCCATCTTTCAGCTGTCTCCAACACAAACAATCCCCCACTCATTAGTGCTTTTGTGTCTATACCAGATGATACCATCTATCACTGTAAAGTTCACTCCCCTTGATCTTCTTTTTTAGAACACAGATGGATCTGCAGTTGCACTGAGAGAGTGAGTCTGAAAAGCGGAAGTCATGAGAAGTTTAATGAGTATGAAGAAGCTCTCTTAATTTGACCTGTTTGAAATTAGGATAATTTCTTTCCGGTGTTCAAAAAGAAGACGAGGCTCTCTTAACAAAGGTATTATATTGTATAAGGACTGCCTGAGGAAAAAGATATCATGTTTTCATCTTCCAGTTGTAATAGCAATACTGTAAGTCCCCAACCTAACTTTCCCTTTTCTTCTACAAATTACCATCCTCATCCTCCTCCTCCTCCTCCTCCTCCTCCTCCTTGTGTTAACCTAGTAACCTGCACTGGAGACATTTTGTTTCACCACATTCATGATTCCCTCTCTGGTCAATTTTCACACCAAAATATTTCTTTA |
| Pbr020246.1 | ATATGTCTCTGTCTTTATTTTTTTATTTTCTTTGATAGAGAATCTAGGTTATTGAATTAAGGGGTACTTTGATCCTGTTGTTGTTGAATTTAGACCCTTTTTTTACTTTTCAATTTTAATTTCATTTCTTAATTTATGACTATTTACTTAAAATTTTAATTTGTTTATTTTAATATGAAAATTAGACCACTTTGAAAATAGTTAAAAGAAAATTTATTTATCATCATATATGATACATGTACCATAGGTTGATAAAATACATTTAACATATGATAGAGGCATCAATGAAAATAGCTAAAAATTAATCTACCTATGATCCATGAAAATAGTTCAAAGAAATTTCATCTTCCAAAGGTTTATAAATTCATACTAGTATACATGTTGATATAATATAATAACTATAACATAAATTAGTTAACACAGTTAACTTGGGATGGAGGTAAAAGAAAAAATTAAGATACGAATTATCAGAAGGAAAAAGAAGAAACAATAATTAATACTTATATTTAATCCTTAAATGAAGTAAAAGATAAACCTATAAATAAAAGATGGTATTTATCATAATTTAATAAAAATATAATAAAAAACCTAAGTCAACAAAGTGGACTTAATCCAGTAATACCTAAGAACTTGGATTTATATCAAGTTTTCTTTTGAATGAATCTATACCAGGCAACCTGCAAACCACGTGAAGAGGTGGGGCAAATAGAAAGATTTTTATGTGTATTCGGAATTCAGGATGAAAAATCACATATTATTATACAATAGAATGAACATTTAAAAAATATTTTCATATAATTATATAATAATATATGATGTTTTAATACGTGTTTCTGACACATTAAAAAATCTTTTATGAAATTAGTACCAGGTGTTTTCCTATCCACTCAGTTGCTTCCACACATTATATTCTAAAGTGTAGAACCAACTTTTTGATGAAAAGACTAAGAAAAAGATAGCTCCTTGTCTCTGCAATAAATTGCCCCCTACTCTCCTTTGCTTCTTTACCTTACTACTTCCTTAATAGCTTCTCTCTCTCTCTCTCTCGCTCGCTCGCTCGCTCTGTGATTATATTCAACCCCTATGGACATATGGTGTTCTTGCTGTGCTGTCTTTCTCAACATCTCTATCTCCTAACCATGACGATCGATGGTAAATGTACGATCTCTCTCTCTGTCTATGTCTTTTTCTAGCTAGCCATAACCTTTTCTCTCTCGGGCAAACACAAAAGGAAACCAAATAAATACAGTACATATAGTTAAAGGTGTACATATTCATATTACTAATACGTACAATATATATACACATATGAATTATAAGTATATATAGCTATCTGAGTAGCACTGCAGGCAGCAGCTGTCTTTCTCAAGCGCTCAGCTGGCCGTAAATACAGTAACACACGGCTGTCTTTCTCAAAGCTGTCCTCCTATAATTTCTCTTACTACTTTTGCAGGAAAGGTGAAAAACAAGTGCACTGAAATCTCATTCAATGGCAATGGCTTCATCATCAAGTCGTTCTCAAAGCGCAAGCCAGGCCCAACGCCTAATTTCAGTCCAAACAATCAGTAAGCCGATATCCCCAAGAGTACAAATTGTACACTATAGATGGTAGCTAGCTAGGGTTAATTTGGAAATTGAAAGCAACCAAACAAGCTAAGAGTCTAAGAGGGAGATATAAC |
| Pbr007075.1 | GACAGCCACGGGGAGGTTCATACTTCATACATTAGTATCATTATGTATAACAAAAAAGACAAAAGAAGGAGAGAAGAGAAAACCTAGCTAAGCTTGGAGCAACACATATAGATTTTTTCTTATATTTTGCTTAGCTTTTGATATTGTTGACTTCTCATGTGAGTTGTGTATAATTAATACAAGGACTATTCTAACTAGTGAAAATTAGTGATGCAAACAATGTGACTTGTATGATGATATGCATCAAAACTGAAACTGTAAACAAATATCATATATGAGGAGTAATTAAACTTTTTTAAGAATTTGTAAGGTTGCTTAATTTTTCGATGTGGGACAACACATCACATTCTCACATGCCCGTTACATGTGGCAATGGTCAAATAAAACAGGTAGGCAATACATATTAGGTTGCATGAATCATGTGTAACCGTTGTGCTTCACGCGTGAGCCAATTGTCTCTAATAACATTTAAGAATTTTGAGGTTTCACTAAAAAATTAATTAGCAATATGGAGAGTAACCAGCTCGTTATAAGGACATGCAAGATCGCTGAACTTTTTGACGTGGGACATCACTCCACATGCAATATTCAATGAAGAAGAATAAGTGTTAGAACTCATTTGGATGTACTTTTAAAATGACTGAAAACACTTTTGGTGAAAATGTTTTTAGAACCAATCCTTAATAAACATGCAAGTAAATTCTGGAAAAGCACTAAAAGTGCTTTTGGAATTCAAAAATATTTTCTTTAAAAGTACTTTCAATTATTTTAAAAGCACATCCAAACGAGCCATTGATTATGGAATATTAGTTGAATCTTCCCAAAACAAACCACAAAATCCACTCTCCATCTCCATGTATGCGTAACACTATCTAGCTACTGTGGGGTGTGTCTATGCAAGGTCTTTATATTTTGAGATTGAGAAGCTAGGTTATTGAATTGATCTATACCGGGCGACCTGCAAACCACGTGGACTGGTGGTGCCAAATACTAGGTGTTTTCCTATCCACTCGCTTCCTTTCACACACAGTGATATTCTAAATGGTAGGGCTAACTTTTTGATGAAAAGATTAAGAAAAAGATAGCTACTCAGCAATAAATTGCCCCCTACCCTCCCTTGCTTCTTTACCATACCACTACCTTACTAGCTTCTCTCCCTCTCTTTCTCTCTCTCTCCCCCTGTGATTCAAGAGTTCAACCCCCATGGACATATGGTGTTCTTGCTGTGCTGTCTTTCTGAACCTCTCTATTTCCTACGATGACGACCGATGGTAAATGTACGATCTCTCTCTGTCTTTTTCTAGCTAGCTAGCCTTCTCTCTCTCTCTCTCGCGCACACACAAAAGAAACTAAATAAATACAGTACATGTAGTAAAGGTGTACATATATACATATACGTACAATATATATACATATATGTACAAGCATATAGAGGTATATATGAGTAGCACTGCAGGCAGCATCTGTCTTTCTTAAGCTAAGCTCAGCTGGCCGTAAATACGGTAACACTCGACTGTCTTTCTCAAAGCTATCCCTTCCCTTAATTTTGATTTACTACTCTTGCAGAAAAGGTGAAAAACAAGCGCACTGAAATCACATTCAGCGGCATTCGCTTCCTCATCAAGCATTTATAAAAGCGCAAGTCTCGATCATCAGTAGACCGATATCCCAAGCGTACCAATTGACCACTAGCTGCTAGCTAGCTAGGGTTAGTGGTGTGCAATTTCATTGAAAATTCAAAGCAACCAAACAAGCAAAGAGCCCAAGAGGGAGATATATAAC |
| Pbr021770.1 | ACAGAAACTACATTATCCAACCCCTCTTTTGGCTTCATCTCATTTGACCACTCGTAATCGCCAAAGAATTGGTCTAACAAACATACTATTGTTCTATCACCAAAATCAACAAAGAACTAAATTAGAATACCCTCCAATCTACATGCTCTTCTCAGTCACTTAAATTGGAGTGACGTTACACTTATCAATCAGTTTCTAATTTAATAATATTTCTCTTTGTCTTGTTAAATACTCTGATTTGGATTTTTTTCTTTTTCATTGATAGAGAGTAGTTACAACATCTGACACGTAATGTGAGTTGTTAGTTAATCGTAACGTGATATGGTACCACAATTGAGAAATATCTTTTTTTTCCGACATTGGTAAGTAAATTTTACCATTAATGGTCACGCTAATACGTTGGGTTTAGCTAGAAAGGAAAATTTGAAAAAGGTAGGAAGGCCTTTAGGATTTTGGCAGGGGCGATGGACCACATCGTGCGAGCAGTGGTAGATAGGCGATGCCAAATTGCCAAGTGCCGACCAACACCGGTGAAAAATCCATTCAAATGCAGCAAAGCAATTTTCCAGACCTTTCCGAACGGCTCCACCTCTGCCTATCCAATTCGTTTTGTGCCGCCCCACTGGAGCACCTTTTATTAGAATAGGTTAATGTCAAGGAAATTAAATTTATCGATTAAATTTTATAAATTAAATTATATGTAAGTTTATGATTGGTTTGTTATTTAAGCGTTGATAAATGTGCTCATTTATATTCGTAACACATCATTTAATTTGCAAATTTAGTCTCTAAATTTAATCTTAATTGCATTACCAATTGGAATAACAACTTATATACAACGGTAATGCTCAGATCTAATAATATATTATTACCGTTCTAATCTAATAATAGATTATTATGTAGAGAAAAACTTGCATATGACAATGGGATGAGGATCCTTTTAGGATTCTCTATGTGAGGATTCTAGAGATCTTCACATACTATCCGTTCATTGTACATCATACATCGTAAGGTCATTTTTCATTAGGTATTGTTTGTATTTAATTTTAAATAAAAATATACAAAATAATTTACGACTGCACGATATACAAACGACAAATAGGATGTGAGTAATCTTAAAATCCTTACAAAAAGAAGTCTGATGAGATCCAAATCCATGACAGTATATTAATGAACAATAAACACTATATGATAGTGGAGAAATTTTTTATTGGGACGGAAACACGGAGATACAGCACGTGTTTTTATGTAAGTTGTGAAATTTTTTATTTTTTTAAGTTATTAACTTTTTAATATACATCACATTATTAGTATAATGATATATAGTGTACCCTTCTATTTTCTAGTCCCACTGAAAAATCTCGCATCGTGTTTGTGAAATTTGCAGATATAAAAAATCAAAAATAGTGGGGCAGTGATGCGTGTGGGGCGGGGGGCCTCCATTCTTTTGATTTCTTCTCTTTCCGTTATATTTCTATATTTTACAGTTCGATCCCATTCTTCCTTCTTCGCGTGTTTGGATATTGGATAATATAGAACGGGAGAAAAAGAAACTGATCAAAACCCTTATCATTTTCTCGCAGTTTCTCGGCAACCAAACACTCCAATTCTCTCTCAAAGTTTCCTTCGATACAAATATTTTCCGTTTCTTCTGATTCAGTTTGCAAGA |
| Pbr001559.1（20） | GATTCATAGTATGAACGGTTCTGATCATATATACAAAACTTTGTGATTAAAACACTAAGAGTTTTGAGTAAGAATTTCGACTCATCTTTGAGTAGTCAAGTATAATTTAGTCTTCCTAACATTTAGGGGTGGAAAAAATTCCCGAAAATCCCGAACCGAACCAAAAAAATCCCGATCCCAAACCAAAAATTTCCCAAACCGAAATTCCCGAAATTTTCGGGATGCTATCCCAAACCAATATGGGATTCGGGATTGGCTTCTCGATATTTCGGGAATCCCATACCGAACCGAAAATATATAATATTAATTATTATATATATATATATTTATACATATATATATTATTTTTTTATAATTGATGCTAGTAGTTTCTAATTCATGCTCTGCAACCTGGAATGCCCTACACCTTGCATATTTTTCTTGTTCTGTTTGATATTCATGTGGATTTTATTATTGCTGCTGCCATGACTGATGATTTCAGATGGCTTAATTCTTTTGTCTCTCAACAGATTGCAAAAAGGGTTTAATTAATTTGAATTTTGACTATGATAAAAACAGTCAGGCATGTCAGTGTTCAATTAAATGGTAGTGTGAATGAAATGAAATTCCTAGTTCATGAATGTGTTCTTGAATTATATATTTGAAGAACAATTCATAATTTCATATTTCAATTTGGGATTCCCGATTTGTCCCAAAATCCCGAAAATATTTCGGGATTCCCGAAATTTGGGATTCCCGAAAATTTGGTTTGGGATTGGTCTTCAAATTTCCGTCCCGAAAAATTTTGGTTTGGGATTCGGATTGGTCTTCAAATTTCCGTCCCGAAAAATTTTGGTTTGGGATTCGGGATATTAGTTTCGGTATGGTATCCCATACCAAACCACTCCTACTAACATTACCCCAAAATAAAACCACTCACCATCGGAAAAATCCATTCAAATGCTGAAAAAGCACTTTTCTAGAACTTTTCGATCAACTCCACCTCTTTGCTGATCCACTTCATTTTGAACCCTCATTGGATCACCTTATATTGAGGAGAACAACTTACATAGATCAGTAATATCTTGATTCTATTGTACATTGTCATATGAGGAAAAATTTACACACAACGATGCTTTATTGGACAGAGAATACCTCATCGTGGTGAGAGATTTTTTAGTCTATTGGCACACAAGCGAATACACTTAATGTCATAGTAAATGTGGTGGGATATTTTTTTGTTAGGTGTTCAACCCCACGTCCGCACAGGGCCACCACCAAAGTAGGTCAAAACACGTGGCGCCATCTCCACCCTACGATCTCCATCACCCTCTTTCCATGATGTTTTTGTGTGTGAAATTTGAAGATATAAAAATAGGGGGCAGTGGTGTGTTGGGGGGCCTCCATTCTTTTAATTTCTTATCTTTCCGTTATATTACTATATTTACAGTTCCACCCCATGCTTCCTTCTTCTTCCCGGATAATATAGAACAGGAGAACAAGAAACTGATCAAAACCCTAATCATTTTCCCGGAGTTTCTTGGCAACCAAACACTCCAATCTCCTTTCCCTCTCACAACCCAAGAACCCATTTAGGTAAACGAGCTTCTCTACCGTAAATTTACACGTATGTTCGGTTTCTTCTGTTTCAGTTTGCAAAA |
| Pbr026562.3 | AATAAAATATGAGATTATTTAGTCATCAAATTGTATAAAAACAAAAGATTGGATATGAAAACATTATGAATTGTCTATCTATTTCGTACAATCGATGGATTAAGGGCGGATGTGATCTTCATAGAGTAATTTATAACATAAATAATTTGAATTATAAACATAAAAATCTGTGAATCGATCACAAAGTATCTTGAGCAGAAACACCTCGTTTATTTGAGGGAGCCAAGCATTCCTCTTTGCACTAACCAAAAGCTCTTACATATTAAATACAAGTTGCTAATTACATTTTAGCTAACAAACAAGAGGAACAGTTCATCTCTTTTTAAAGCAACAAGTAAGCAGTCGTCTTCCAACCCTCCACCGATCTCTATACATTAGAAATCAATGGGTTGGGATGAGCTGTCCTGCTAGTGCATTAGATGCTTTTATCTGCATGCTTACGCGCGTTGTCTAGCTGCTTGCTGCTTGCTTCCCTGCCCTCGACTGGGAGAAACTTGACTCACCCTCCCACTCCCTCCCTCCCTCCTATATCTGTAATTTACCATTTCACAACATATTCTGGTCTGACTCTGAAATTCTGCAGAATGGTAAAAGAAATATATGAGTGGAAAACTCTGATATTTATCTTGTTTATGAATGTGGGCACCTCTATTATTGTTAAAAGGGCCGCTGGAGGACTGTTGAGAAGACCAAGTTGCCCCTAGAACAGTGTTGGGTGGGTGGGTGACTTTGGGTTTTCAAAGCACAGAAAAAAAAAGATGGAAAATTAAGGAGAATGCTTATAGAGAGAGAGAGAGAGAGAGACTGTTGGATGTAAAACCAAAAAAATATGAATAAAATTACTGGGGACGAGGAGGGGTTCAGGCAGGCATAAATGTGAGTGTAAAAAAGAGAGGTGGATTCTTATCATTCCTTTTACCAGACCCCACGAGTGGAGGGCGGAGGGCGGAGGGCGGAGAGGTTTTCTCTTTCTCTCTCTCGTCTTACTTCCTTCCTCATAGCTAGTAGCTCCACCACAAAGGTCCCTCACCTGCTTGACCTAATTTCTCTCTCTAAAGAGTAAGTGCCACTTCTGGTGCTGCGCAGGGGAAGGACAAGAGTTAATCATAGACCAAGAAAGTAAGAACCCCACAAACTCAATCTTAGTCGGTCTTAGCTTGGTCCAACTCCAATCCAATCCCTGCAATATTTTTAAAGAGATTCTCACTCCACCCCAGCCCCTCTCTCTCTCTCTCTCTCTAAAGTCCAAAGTCCAACCCAGCTCAAGAGAGAGAAAGACAAGAGAGAAGAAAAAGAAAAGTACTATTGTGGATGACTGATGAGTGAGAGAGAGATGGAGTTGATCAAGATCAATCAGCCAACAGCAACTTAAAACCCAAGAAACAAAAAAACACATAAAAATAAAGATATCATCTCTCTCTCTCTTCCTCCAAGTCCAAACCCTTCCCTACCAA |
| Pbr018814.1 | ATAGATTTGAATGGGCTGACATTCTAGAAATCTGAGGTTTTCAATTATTTTTAAGAACAAAGCGACATAACTATCCTCACATGCATATACTTCCATTTAATAGAAATTGGATTGAATGAATGAAAAACTACCAGCAGGGTGAAATTGGCCAAAAAAATGAGCTTAAGTTCTTAATTTTACAATGAAAAATTTTAGGGAGAATAAAAAAGTTAAGTAAAAGTTCAAAGATGAAATTGGCAACTTACTCTACTCCCAAACAAACAAAAATTGAGTAACTTTTAGACCATCTTCAACTCTTGGATGAAAACCTATAATTTTTAACTTAGAAACAGTTTTTCTCCTCCAACCCTTATGAATTAAATTTTTAGCCTGAAATTATTAAAGAATGAATTTAGGATATTTTTTTTTCTTAAAGTAATTTAAAAAAAAAATTATGTTGACTATCCTAATTTAATTTTATAAACATTTTAACTTACAAATATTTAGATTCCAATAAATATTAAGAAATCACTAAACCGGCACCATAAAACTCGTGGAACACTACGAAAGAGTATGAAACACATAAAATAATTTTTTTAATTACTTTAGCCATTGGATTAAAATTTGGACCATTAGATTTTTATTTTTTTATTTTTATTTTTTACTGTTGTATTTGATTAAATTAGATCTTAGCCATTGGATTCAATGAATTTATAAATATAAAACTAAAAAACATACACATATATAATGAGCTAGCCCCATTAACCCAGGGGAATCTTGGGCTAAATTTGTCCCATAAATTAGTTTTGGGTTTTAACTCATATTTGCTCCAAAAGTTAGAACAAGTTAGAATAAATTTAAAACCTAAAATTTGAGTTTTATTCCAAGAGTTAGAGTAGTTTTTAAGAGTGCATTTCTCATTGGGATCCATCAAACACCTTGACTCAACCCGAACAGATAGGACAAGGATCCTCTTCGGATTCTCTTTGTTAGGATCCAAGGGATCAATCAATCGGGTCTGTTTATTGTACATCGTGCGGTCAGTTTTCGTTAGGTACTGTTTATATTCAATTTTAAATTTTAAATTTTAAATAATTTATAACCGCACAATATACAATGAACGACCATGATTAATTGATCTCTTAGATCCTTGTAAAGAGAATCCGAAGAGGATCCTATTCCTAACAGATAAACCCTACTAAACGGTCCCACCACTACTCATCACAAGTGTGGCTCCCACATATCGGTGCTGGAAAGGAGAAGAAACCGTAGATTATTTCTCAAATATTTTCTTTCATATTTATTTGTAATCAAATTAATCTAATCAAATCCACTAATATAAATACCGGAGAATATCCCCCCAAACTTGTTGGTTTAAGCACATTGAGAAAATTCTTCGTCTTCAAGCTTTTCCCTCCACTCTTTGTCCTGCCCCCACAGTTTTTCTTTCTGCAACTTCAAATCTCTGATTCTACCTGCTGGGACACGCCGAGTTTGCTTCTTCTCAACTGGGTTCCCTTGATTTTTTTCTGCAAAC |
| Pbr039926.1 | AGCAACTATAATTATATACTTATTAAATTCGTCATTCATGAAAATTCAATCGCAGAGACCTCTCAATTATAAGTTAAAAAACGAATACAACTAAGTAGTACTACTTGTCACACCACTTGTGAACTAATGTTTGATCAACTTTCTTCCTCAAACGAGACTGATTTGGAGGAATCAACTCTCTAACTAAGTCAATAAATATGAAATCGTTTAATCATTAAACTGTATAAAAATATATGATCGGATTGAAAAAGTATTATCTATTTGCTACAGTTGATTGACTAAATGACCATTGTTTTAATTTATTTTTTTTGCAAAAATGATCTTTACATATTGATTTACAATATTAACGGATCCGATCAAAAGCATGAAGTTATGTGAATTGGTCACGAAACATTTTGAGAAGGAATTCTCCCTCATTCTTGAGTAGCCAACCATTGTTCCTTTTCCAGTAGGAATTAACTTGGCTCCTCAATGGATGAGTAGGAGGAATTCCTCCTCAAGATGTTTTAATTATAACTTATTCACATAACTTCATACTTGTGTTAAAAACTGTTCATATTATAAATCACTATGTAAAAATCATCTTTGCAAAAATTCAATAAAATATGAGATTATTTAGTCATCAAATTGTATAAAAACAAAAGATTGGATATGAAAACATTATGAATTGTCTATCTATTTCGTACAATCGATGGATTAAGGGCGGATGTGATCTTCATAGAGTAATTTATAACATAAATAATTTGAATTATAAACATAAAAATCTGTGAATCGATCACAAAGTATCTTGAGCAGAAACACCTCGTTTATTTGAGGGAGCCAAGCATTCCTCTTTGCACTAACCAAAAGCTCTTACATATTAAATACAAGTTGCTAATTACATTTTAGCTAACAAACAAGAGGAACAGTTCATCTCTTTTTAAAGCAACAAGTAAGCAGTCGTCTTCCAACCCTCCACCGATCTCTATACATTAGAAATCAATGGGTTGGGATGAGCTGTCCTGCTAGTGCATTAGATGCTTTTATCTGCATGCTTACGCGCGTTGTCTAGCTGCTTGCTGCTTGCTTCCCTGCCCTCGACTGGGAGAAACTTGACTCACCCTCCCACTCCCTCCCTCCCTCCTATATCTGTAATTTACCATTTCACAACATATTCTGGTCTGACTCTGAAATTCTGCAGAATGGTAAAAGAAATATATGAGTGGAAAACTCTGATATTTATCTTGTTTATGAATGTGGGCACCTCTATTATTGTTAAAAGGGCCGCTGGAGGACTGTTGAGAAGACCAAGTTGCCCCTAGAACAGTGTTGGGTGGGTGGGTGACTTTGGGTTTTCAAAGCACAGAAAAAAAAAGATGGAAAATTAAGGAGAATGCTTATAGAGAGAGAGAGAGAGAGAGACTGTTGGATGTAAAACCAAAAAAATATGAATAAAATTACTGGGGACGAGGAGGGGTTCAGGCAGGCATAAATGTGAGTGTAAAAAAGAGAGGTGGATTCTTATCATTCCTTTTACCAGACCCCACGAGTGGAGGGCGGAGGGCGGAGGGCGGAGAGGTTTTCTCTTTCTCTCTCTCGTCTTACTTCCTTCCTCATAGCTAGTAGCTCCACCACAAAGGTCCCTCACCTGCTTGACCTAATTTCTCTCTCTAAAGAGTAAGTGCCACTTCTGGTGCTGCGCAGGGGAAGGACAAGAGTTAATCATAGACCAAGAAAGTAAGAACCCCACAAACTCAATCTTAGTCGGTCTTAGCTTGGTCCAACTCCAATCCAATCCCTGCAATATTTTTAAAGAGATTCTCACTCCACCCCAGCCCCTCTCTCTCTCTCTCTCTCTAAAGTCCAAAGTCCAACCCAGCTCAAGAGAGAGAAAGACAAGAGAGAAGAAAAAGAAAAGTACTATTGTGGATGACTGATGAGTGAGAGAGAGATGGAGTTGATCAAGATCAATCAGCCAACAGCAACTTAAAACCCAAGAAACAAAAAAACACATAAAAATAAAGATATCATCTCTCTCTCTCTTCCTCCAAGTCCAAACCCTTCCCTACCAA |
| Pbr020546.1 | TGAGTATCTGCGTCAACACGACAAATATGCAACCGGTGAGTTTCCAACTGAATTGGAAAGCTCGGCCATTGTTTACATCATTTGTTTCATGGGTAATCATTTACTTGCATCTTGCGCATTGGTGAATATACGATTGGATGCAGGGTGCTAAAGTTTTTGCTTGGTCATTGAACTTACTCATTTGAGTAGAGAGAGAGAGAGAGAGTGGTGCAGATTATTAGTTAGAAACAGAGCTCAAAAGATACATGTTCAATTTGCTTCTGGTTTCAGAGGCAGTCGGGCAACAATTCAGGTTTCGTAGTCAAGTATTTATACATTTCCGTGAGTAATAGCAGCAGCAGAAGAAGAATAGGGACCATAGGTTGTCTGTTTTTTGCTTCGACGGCCGTCAAATGCTTCACGTTTCATCGTTGAAATGATTGATCGAAGGACGGGTGATTACAATTTCTTTAGAGTAATAGGGTCCAGAATAGAACTTCGATAGTGATATCCAAACTTGAGGTTGAAGATGACAGCAGGATCGTTGCTTTGTCAAAGTCATCGATTTTCTTTGTTACCTTGGATTGGAGGGAATCGCACTCAGTCTATCCTCTTGTCCTCCCTCTCCGTTTTATTGGACTAAATTTATAAGAATAAATCGATTAAGTTGGACATTGGTGGGTGGGTCCATGGTTCCCTCTCCTCCTCTCATTCAATATATGTGCACTATTTCTCTAATAGATAGAGATGAGATGGAACTCACGCACGCTGACGGTTGATACGAATAGATGGTAAGTGTAGCAGTACTCAATGTTTAATCCAAATGACATCCTCATTTTATTCCATTTTAGTTGAACATATACAACTTAAATTTCCACCCTCATCACTACTTAATTACTAATTTACCACACAACCATTGATGAACTACAACGTAATTAAATCTAAACCACAAAAACCACTAACACAACTCCCCCTCTACACCAAAACACACCCCCCATCCCCATGTTGACTAAACTGTCTCCCCCCACCACCTCCGTTACACATGGCTGCTACAGCCCCCCACTGTTTCCTGCACCCTCTGCTTTTGATATACACACCCACCGCACCTACTCAGCACACCCACATTGTCCTTGTCTACTCATCATCCCCAATTCCAACTCCCACAACCTCCTCCTCCGCCTGCCCCTCCTCCACCTCACTCTCCCCCGTCTGCAGCAGCAGCGACGTGAACGGCATGTGCCGGTATCCCGCATTATAGTCCGCCCCCATCGGCTGGCACAAGTCCAGCTGCAGCTGCATCGCCTGATGAGCCTGGGCCGCCGCCGGGTACAAGAACTGCCCGGAGCTTCTGAGCGACTTGAGCGGCTGAACCCGGCAAGTGACGGACGGCATTTGTGACGACACGGACGGGGAGGTGGTCGAGATCGGCTCCGCCGGGAGTGTGCCGGAGCCGGTGGCAGCGATGATGGAGGGCTCGGCGTGGCGGAGGAGCCACTCGATGGTCTCGCCGTCGGAGCGGTGTCCGAGCTCCCGGGTCAGCTGGAAGATGCGGGCGGCGCACATGGCCGGCATCCGGACGCGGCGGCCCCGTCCATTGACTTTTGTATGGCGGTCCCTAGTGTTCCGCCGGCTGACCTGTTTCTGGGGCTTGGGCTGCGCCTGGAGTTGGAGGTCGGATAGTTGGGAGAGCGACGGCGCCGTTTGGATGGTGGCGGAAGTAAGAACGGCGGTGGGCAAGACGGTTGGCTTTCGGTCTTCGAGTTTGTGGAGTGGCTGGAGGGCCATCTCGGAGGCCAT |
| Pbr035636.1（25） | TCATTATCGGAAATATTTTTGTTCACTATTATCAAGTGATGACGATGATCATCATCTTATTTATCACTATTAGATAAGTTTAAATTTTAAGACTTGTGTAGTGGATAAACATAAATCTCAATATTTAAACTCATCCAACAATGATAAATAGAATGATGAGCATTACCACCCGTAGAGGATACATAATTCTAACATTGGTTGGTCTAATTATGGTACGATAAGCGCTAGTCGAGCGGCGAGTTAGGACCTAGGACCTAGCCCCTATACACCTAGTATTTTAGTGAATTTTATGGTATGAGATGAATACTCAAGTGATTACGAGTTCGAGTACTTTCAAAATATTTTCTAGAGATTGTATGAATTTATAGTAAATTTATATAAATAACGGTGTGAAACATATAGTACAAAAAGTGCTGGTTAAGTAATACACTATTTTTTTTATTAATTAAAATGCTAGGTGGCATAACTTGATTGGACAATCATTAAGAAAAATAGGAAATTGGTCACGTGGAAAACGTAGAGAGAGTCTTTCTTCTTAGTTGGAACCAAGGTTCAAGAACATCAAAGTCATAGTGGTCGTCTCTGTGTTCCTCTTCGTTTGAAAGTAAATATTTCTTCTTGGTTCAAAGCTTCTATTGCACAGTATTTGCACAATCATGATGGTCTTCTCTCTTCCACCATCTGCTCAAACCGTTAAATGGTCTTCGAAGGTCCTCACTTGTGCTTCTTCGAACTTTTAGGTGGGCCCGAGACAACTCGGGTCCATATATGTATCCGCCCCTAATTAGAGTTTTGAGCAAAAATGCACCCATTTAGATCAATGGAGCTTTGGCCTTGGCTGTATCTCCCATTTTTTGAAGATTGATTTGTCGATTAGTAAAAGAAAATAGGGACAATAATGCCTCATTGAAGAAGGCATGGCCTACTTAATTATTATTTGCACCAAACACTTTGACCATTCTGGAACCATTGTGTTGAGTTAGGTATTGTCAGTCTGGAATAGTCGGAACTCTGATGTTTCATTCATGTAGACTGAGCGACTGACCGTACATTTGCATTTACTAGCATGCCAAGCCAATCAGTTACTAGTTTTCAGGCACAACAGACGGAGTGATTTTTTGGTCTGATCGCCGCTGGTACGTGTTTTCATTAATTTATTCATGTTATAATACGTAAAGTTCTATTACAAAAATAAGAGATGCTTATATAAGAAGCATGATCAAAATAAATTGGATCAAGATTACCTGATAAACTCAAGTTGGCCAGCTCATTAACAACAAAGTTGATCTTGCCATAAATATTTCTGAATTCACATACTCAATTAATGTGTAAACGATCACGGCAAGTGCGCTCGCGATAAATTAGGTCTGATATATCCCACTATGTCATGTATTCCAATCGTAACATATAACTTGATAGTATAACTATCACACTCAAAAACTTTTCTGGACAACCTATTATCGTTGCCAAAAATTCAACTAGAAGACGGTGTGACAATGGACTATAAGACTTACAAATCACCAGAATCTAGTTGTTGATCTACAGCTGCAAATTACGACATTGTGAGATGTGGCATTACAAGTAGCATGAACTTTACTTGGAAACTTTATGGCTTCAATAATTGAAGTTGAAAATATTTCCATTGACAAATCTAGGCGTGCCAATAAAAATACAACCCCACCTTCAAAACCCAAAACTCAATCACCCCCAAAGTTTCCACTAAACCCTAAGAGCTTAGGGCTTATCATTACATCACCATCCACAAAGAGGTACCCACCATTTTTGGGTCCTCAGCACTGATCCCACAAAAGCAGAC |
| Pbr013906.1 | TGAGAAAGTGGGGATAATGTACAACTCGTAGCCTCGTAGTACTGCAAATTATTATACATAACCTACTAAAGGAATTTTTTTCCATAAAAAAATTGAAACTAGGGAACATTCTTATTAATTTCTTGCGACTCTTCGTAAATGCAAAGAAAATAAAACAAGAAATAGGGTGAATTTACTTCGAACTCATTTGTTCATATCAAAGGCAAAAAGGACTTATAGTTGTGCTCGTCCAGGCAGCCCGGAAGGATGCATACAAACCATACCCTTAGATTTTAGAGAACTTTAACAAAAAATTCTCGGTATTCACTTTAACGAAAAATCACATTTTTACACTAAAAAGTCAATTATGGTATTATTCACTTTACCTTTTATTTTGTATTTTCGTTAAAATTCAAAGTTTTCAAATCATTTTCATTAGTTTTTCTTATATTTGAGTCCAACTCACCTTTATCGATCACATCTCTTTGTGTTTGGTACATTAATAGGAGCATCCAAGTACAAAAATATAAGCATAGCAAGACCGAGTCATTAGTTAAGTGGACTTAAACGAATATGGGTTTTTTGAGCATGACCAAACTACATGCTATTCGGATTCTTAAAGCTCTTTTGAAAATACATTTAAAATGATTGCAGTATTTTTATGAAAAATAATTTTAGAACTAATTAGTAGTAAAAATGCTAGTGAATTTTACATAAAACACTTCAAGTGTTTTTGAATCACAAAAATAATTGAATCGAAAGCATTTTAGTCATTTAAAAAAAAAATTCAAACTTGCCCTTAAACTTTTATTGGCACTCATTAGGAATAAATTCTGACATATAACAACTATTTTTTCTACCGTACTTTTCAAGTGAATTGGTCATAATTTTTTTTTCTTTTTTCGCAAATTATTTTTTTTCTGACGAATCATCGTGTGGTTGTAACATTAAAGTTGTAGGCAAAGTTTTGAAAGCTAAAGGAAATGAAAATTGATGATTAGTTTATTAGTTACACATTGATAAACGTGTTTATTCCTATTAATAATATCATTTAATTTGTGAATTTAATTTGCTAATTTAATCCCTTTAGTACTACCGAGCGTGAAATATTAAGGCAATTTAAAACAAAACAAGGAAATAAAAATGGTCAGAATTTGGAAAGCATGTGATATGAGTGTTCTTGATGATCACGTGGCATGTTCTGATTGCTACGATCCTTTTCATCAAAATGAACGACCCTGCAGATGATCATCTGCATGTAAGCCCTAATCTGAAGGTACGGAACCCACAAAACAAGACTCAACAACGTGGCAGCATGTGCATGGGTCATGATGTGTGTACAAATTCCAGAACCGTTGATTGGATTCCCTTCTCCTATAAATAGATAGATAAAGCATTGCGGGGGGTCCCCCTCAGTGGTAGGGCTCGCAAGGAGAAAAAGGAGGAAAAGTGGGGGCTCTTCTGATTCTCTCTCTCTAGGGTTCGTTCCCTCTCTCCCTCCCTTTCATTTTACTGTTCCTTTTAAGTTTCATAATTGTTCAAGTATTTGATTTTTCTTTCCATAATTAGTTCATTAATCCAATATAATCCTAAAGTACGAACAGAGATTTAAAATCCCAGTAAAAAAAATCGTCCCCTGTTCTTGCACTGTTTTCTGCAACTAACCCAGATTCTTCCTGCTCTTAAATTCTTTTCAGTTCCTCGTTTTACTGCTTGATTTTGTGTTTTTCCATCTGGATTTTTTCTGGATTGTTCAATTTTTCTGAGATTCTTGAAGTCCGGCAGATTTTTGGAGAAG |
| Pbr041545.1 | ACGATAAACGGACACAATTACAGGATCCCCAAGAAAAAGGATCCTTGCTTTACATTCACAACAATTATATCACTGCTCATGTCTCTTCTTTGACCTCTCTAACTACATCTTTACAAATATTCTCACCCTGTCGATGGATAAAATTTTTAATCCATGATGATACAGCAGGTGTTAAAAGAGACCATAAATCCAAAAATCAATAAAAACCCAGAAAATCGGTCAAAAGCTCTAACGACTAAAAATCAATCAAAGCCCAGAAATCGATCAAGACCCTGTATAATTTCACGAATCTTCATAACAAAATCAAAGAAAGTGAAGAGAAAATGGAAAAAGGAAGCCATGCGAGACTTATGAGGACGCAGCAGTGCTTAGAGTGATGGTTTGGCGAAGTTGTGGAGCAGGACGAAGACGATGCGGGGAGTGGTGGGTGACTTCAAATTAGTCTAGACAGATAAGGAGTCGAGCAGGACGAGAGAATGGGCTTAACAATCCGTTGGTTTATGGGGGGGTCTCGCTAAGACCTTTTAGCTAGCCCTATAGTTGGGGCGAGTGAACTCTAGTCCCACTAAAGTTAATCCCTTCAGCCAACAAACATGTAACTAGCTAATTCTTAATCCAGTCTAGTGCAGTGGAACTTAAGGAAGGCAAACAAACGCACCTTTATAGTACAATGATTTATGATATATATTATTAGAACGATAGAATGTGTGTTTAATGATTTAAGAGAATTTAATGGGTTAATTAAAATTTAAGTGATAATGTAATATGATACATTGATGTTTGGTAGAAAGAATCGTCTCTTTTATCTTTATACATAAAGCCAAGGGTCCAATGAAAAGTATTTTTGGTGTCACAAGCTTCACAAAAAATAATTAGATAAAAATGCTCATCAAACAAAAAACTTCAAAAGCAACCTAAAATAATGCATCAAATATGGAATAGGTATTTTCGTCATTTTAATAGTGTTTTTTTAATAATTAATTTCTTTTGTACAAATTTTTTTTTTTATAATTAATACCTCACAATAATTTAATAATAATGTGATTTAATTAGTTTTTCATTAAATATTACTTTTTATATTTTTAAACACGTTCAAAACATTTTAAGAGCCGTGTAATGTCGGTTATAAAAAAGGTCTTTCGCATTTGGATAATAATGTGATTAAATTAGTTCTCAAATGATTGTTTTTTTTTTTTAACAAACAATATTATCTTCATTAAGGGGGTGGGGTGGGCTTAGCCTCACAATGAGTTATTAATAATGTGATTCAATCAGTTCTTTATTAAATATTATTTTCTCTATTCTTAATATAAATTCAATAGACACCGATTTTAATACGTGGTTTCAGCTGCTTTAATTAGTTTATGAGGGGTTCCTTCGATCTAAGATTATTTATTCACTTCAAATATTTGACTTTCATTTTGTCAAATACATTTATGCATATAAATATTTAAAACGTGTAAGTCGTGCGCAGCACGCCGTAATTAAAAATATATTTTCTACACTAACTTTATGTAAATTGCAGGAGGAGAATTCAAATTTTAAATACAGAGTAGAACACATTGTTCTTAAATACTTTCCTAAACTCATATTTGCTTTAGAAGGAATCTTATCTGACTAATCATGTATTGTCGTAAGTTGAACTTGTTTATATAAATGTGTAACTAATTCGAAGTACCGACACACCGATATTTCGAGACCGGAGAGTGAAAACACGAAGTTGGCAGTTGAAACTTGAAGTTGTGTTCATATCTGCCGAGTCAAACCCCGCTACAGTGTCAAAAGTGGCAAGTGAAAAAGTAAGAAAGGACGGACAGGTTGGAGAGAGAGAGAGAGAGAGTAAAAAAACAACCAAATGCAGGCTGCCGGGAGGAGAGAGTAGAATTTGACAATGGGATACGTTGGGATTTTAATATCTTGATGGTTTTGTCATCTCTTTCTTTACCAAAACAGTGATAACTCATGAAAGTGTAGGACTTTTCAGGTCACCTTCTTCAATCAAAGAGGACCCATTTTTCTCTTTTTTCATTTAATTTTTGTAATCCCCTGTTC |
| Pbr039105.1 | GATTATCCAATTGTTTACTATAAAGATAACTTTATTCTTCTTATCATACATTATTACATGCCATATATGCATGCTTCAACTATATGTAAAATTTTAACAAAGTCACAGTTTTTTTACAGCTTGTATATTAATCCTTTATGTGTGTTTGATAAATGTAATATTTGGAACTTTACTATGGTATATTATTATCCATTTATTGTTAGAAGATCCTCGAGGAGGTGGCATAAAATGGTGGAAAGTGGGAACCCTATTTCCAAAGGTAAAGGGCAAAGGTGCAAAGGCAAAACTGCAATGATTACATGAATGCGATTGGAGCCACTAGATAATCTTTTTCCTGTTTAAATTACCAATCAGTCTATTAGGGTTTTAAGAAATGTTAAGGGAATTTTCTTAAATGTGAGACTTTTTACAGATTCTCTACTATACTTCACAGTTTAACTTTAATTTTATTGTTAATATTATAAAATATTATGTAAAAGATTTGAGGATACAAAGAATTCATAAAAAATTATACTTCTAAGAGGGCTCCTTAATCTTTCTATGGGGATTTTGACTTGGAAGAGTGGGTCCCCACCTGCGGCCACCACCTTCTTCATGAATTGAATGGTAAAGAGCACCACACGTGGAGTCTGAATCAAGGAACTGTCCAACCACAATAAAAAGGACACAACTCAGCAAATTTAATTTATATTTTTGGTAGGAAATCAAACGAAAAAAAATGAGAAACGTCCAAAGTTGCAAACAAACTCTCATCCTTCTAATGAATGAAAGCAATAATTTCAATGGAGAAAACTATTGTTTTTAAGAAAAAAAGAGCGGTAATATTCCAAGAATTATAGAAACAAGTTCATGCAGACATAGAACATGATCAATAAATAAAAATAAATTTTAACGAAAAACTTTCGGTACTGTTTACTTTAACGAAAAATCATATTTTTACATTAAAAAGTCAATCATGATACTATTTATTTTACCATTTATTTTGTCCTTATCATTAAAACTCAAAGTTTTCAAGCCATTTTCATTAGTTTTCCTTAAATAAAAAATATTTTATCATAATGGTAAGAAACATCAATATATATGTAGAACAAACATGATAAAATTTGGATCATTAATATATATGTAGAACAAACATGATAATAAAAAATATTTTAAATTGTAATTGAATGATTCAACCATTAATCGTTCAAGTATTCTTTCAAGGATCATCCCTTATGTCTCATCTTACAAGGCGTCCCTCAAACTTATTTGATGAATTCTTCAATACAATAGGATTGTTAGATTATTAAAATAATTATTTTGGCCAATCCTTAACATCATATCGTCACGAATCTAAATGTAAATCTGAAATAATTACAACTTAAAACAAAGAGAGAAAATTGTTCTTTATAATATGCATGTATATATATTTTAGATTTTTATTATGGATCATAGGCCAACCGTATAATTCCCACTCATTATTTTTGTTGCTGAAAGCTTGCCTGGCCTCTTCCTTTGAGTCTTGACCCTTATCCTTTCTTTTCTGTTCTCTTCTCTTCTCTCTCAAACAAAAGAGAGAAAAAAACAACTCAACTCAACAGCTACTGAGCTGCCACCTTCCCCCTTCTGCAAAGAATTTCATGCTCCACAGTCCACACGAGAGAGACCCACTAGGCCAGGCCAGGCCACCACAAATTGATAGAAATTGCGCTGCTGGGTCAGCTTTCTACAGTTGTTTGTACCAGGAGAAACCAAGGAAAAAAAATAAAATAAAAAAATAAAAAAATAGTGAGTGATCGATCGTCCCCTCCAT |
| Pbr037196.1 | CTTCCTCGCCTCTTACAACCCTTTCAAACCCAAATCCCACAAAACTCGAAACAAAATAGCAAAACTTTTTCAATTTTTCCCTTTTTCAAAACCAAATCCAACTCCCAAAATTTGAACCTAGAAAATAAAAAAAAACCCAAAAAACCCAGAAACCCAAACCAGATTCAACCCCTATCTCCCACCTTCCAATAACACATGCATCCCGCACCCAACTCAAAATTCAACCCCTATCTCACACACGGAGGCAAAGAAAAAACAAAGGGAACAACAAAGCAAGGAGCAACCTGGTATAGAGAACAGGAGGGCGAAATAAGGTAGAAAAAGGGGAGAGGGGAGGAAGACAGTGGCGGAGAAAATAATAGGAAAACATGATTTTGAAAAAGACCAAAAGAAAAAGTAGAAGTAACAATATAATATTAATAAATATAAATTAAATTAATAATAAAATATTAATAGGTACAAATCAGTCCAGCTTAGTATATTCTAAGCTAATCTAACTTAATCTTTGAAACTAGTCCAGTCCGAGATAATTCGGTACAACAAATTCACTTTTAATGTACTAAGCCGTGTTCCCAACACCGATTTAATAATATAATATTATGTAAAATTACGTAACATCATATTTTTGAATTAAAATACTACTGCGGTCTTGTTCCATGCAAATTACTTATAAGTTGTATTTTGTGTAAGTTGCTCAACAGCATGCTTTGTGGTCTGTAAAAACTCCATATCATTAGCCTTGATGTTGCCGAATGCTGTTGATCCCATTTATATAATCATGTTCTTTCCTCTCTGTCTTTTTAAAGTTCCCATCAATTTTTATATCATATCTTAAGGATGGTCCTAGACCTAGAAGAGTGGGATACAAGCAAACCCCACCACCAGCCATTCATAGTTGCCAGGTGTCGAAATCTCAAGCTGTCCAGCTTGGAAGTTGAGTTCCTTATAAGAGATGGCATCATATGTTGGTTTGCTACTGCAATTATAGCATATGCAGGCAGCAGGAGAAATCAATAATGAAGAAGGGGGAGAAGGGGGAATCCAATCAATCCATAGACCATTGTCCATGTGACTCCATAGAATGTGGGGAAAATTAGAGAATGGGCTTTTGATTTGCCTGCTCTATTATTTTAGCGTACAATAATGTATAACGTATGTTATTGAATTTAATGATAAGATGTTTGTTTAATAATTTAAGAGAATTTAACGGGTTAATCCAAATCACTTAAAAGATAATGTGATATTACGGGAAAGAAATAATTGTCATGAGAGTAAAATTCAAATTTAAGCGCAGATAACTAAACTTGACAGAACTCATATTTACATAAAAAAAAAAACTTTATCTGACTAATCATATCTTGTTCTATGTCTAAGCTTATTAGCATTTAATATGTGATTAATTTAAAATATCGGCACATTAACATACTGACGGTGTGAAAACACGAAACTGGCAGTTGTGTTCATATCTGCCTAGTCAAACCCCGCTGCAGTGTCAAAAGTAACAAGAGATAAAGTAAGAAAGGAGGACAGCTCGAAGAGAGAGAGAGAGAGAGAGAGAGAGAGAGAGAGTAAAAAACAACCAAAGCCAGTCTACCCGGAGGAGAGAGTAACATTTGACAATGGGGTACGCTGGGATTTTAATAGTATTTTATGGGTTTTGTCTTCTCTTTCTTTGCCAAAAACAGTGAAAACTCATGAAAGTGTAGGACTTTTCAGGTCACCTTCTTCAATCAAAGAGGACCCATTTTTCTCCTTTTTTATTTTTAATTTTTTAATCCCCTGTTC |
| Pbr018420.1 | TCTATCAACAGTTGGGACCAGCTAGAAAGGGAATTCCTCAACCGTTTCTACAGCACTCGTCGTACTGTAAGCATGCTAGAGCTGACCAGCACGAAACAGTGGAAAGATGAACCTGTCGTCGACTACATAAATAGATGGCGTTCTTTAAGTCTGGATTGCAAAGATCGGCTTTCTGAAACCTCTGCCATTGAGATGTGCGTTCAAGGCATGCAGTGGGGACTACACTACATCCTTAAAGGCATCAAACCACGAACATTCGAGGAGTTAGCCACCCGCGCCCATGATATGGAGTTAAGCATCACCCGTCATGGGAAGAAAGAACAGATCGCCGACTACAAGAATGACAAAGTTTTGGGGCCAAAGGTGGATAAGGCTACGTGGAAACCCACCAAGGAAGCAATGACGGTCAACACAACTCCAGTCAAAATCCCTACACCAAGCAAGGCGATTCAAACCGAAGCTTTTCGTGATCAAGAGACACGTAGACGCACTTTGAAGGAGCTTGAGGAGAAGACTTATCCATTCCCCGACTCTGACGTGGTTGCCATGTTGAAAGACTTGCTTGACAAAAAGGTGATCAACCTCCCTCAGTGCAAACGGCCAGAAGATATGAACCGTACTGACAATCCAAGGTACTGTAAATTCCACCGCTTCATTAGTCATCCGACGGAAAAGTGCTTTGTGCTAAAAGATCTCATCATGAAGTTGGCTCAGAAAGGGATCATCGAGCTAGATCTTAATGATGTGGTGAAGACAAACTATACCACCGTCACTTCTGGCTCTTTGAACTCAAAATCTTCACCTCAACCGCTGGGGGCATGCTCCAAAACCATGTCAGTCAAGTCAAGTGAAGTTGAAGGATGGACTTATGTTACTCCAAAGAAAATGCACAAGAAACATAGGTCTCCTCCACAAGTTCACCAATCGGAAAGGGGGCAAAGCAGCTACCGTCAACCTTCAGAGCTACATGAAAGTGTTGAGGATGATGAAGTTATGACACAAAGATCGTCCGTTGCCATCACAATGCGCGATTTCTTCCCCAAAGACTTCTTCAATCACTTAGTCAAGACTCCTTGCTATGAAGATTGCAAGGAATGCCCCTCTAAGATCGTTTGACAAATTCAAAAAAGAGCTCCTCGTTCGCACGAGCCTAAAAGGCGACAACCAAAGCTCCTTGTCCGCACGAGCATAAAAGGCGACAACAAACGCTCCTTGCCTGCATGAGCTGAAAATGCAAACGGCACCACCAAACGCTCCTGGCCCGCAAGAGCATAAACTGTGTACGGCAAAATCATCATCAAAATCATCATCAAAATCATCACTCATTTGAACTACGTCATGACTTGATCTCTTCTTTGGAAGAGTACGTAGGCAACTTGAAACTTCAAAACTTCAAGTACAGTCACATCAAAATAAATAAATAAATGTTTCATTAAAGAAAGTCAATTTTATTACAAAAGAAAAGAAAAAAAAATACATATGTTACTATGTATTAGAAAAAAATTACATATTCTTTGTATAAACATATACAAATCCGCTTAGACCCTCTGGACCTTAAGGCCCTAGACAAAACGGACAGTGCAGATCCTCGTTGAAGCCCAAATCCGGGCCCAGCTTCAAAGCAAAGAAAAAGCTTCCATCTTCTTACTGTTTTACTGCTGGTATGGAAAAATTTGTGAGGGTAATTTCC |
| PPbr039901.1 | ATTTTGAGAAGGAATTCTCCCTCATTCTTGAGTAGCCAACCATTGTTCCTTTTCCAGTAGGAATTAACTTGGCTCCTCAATGGATGAGTAGGAGGAATTCCTCCTCAAGATGTTTTAATTATAACTTATTCACATAACTTCATACTTGTGTTAAAAACTGTTCATATTATAAATCACTATGTAAAAATCATCTTTGCAAAAATTCAATAAAATATGAGATTATTTAGTCATCAAATTGTATAAAAACAAAAGATTGGATATGAAAACATTATGAATTGTCTATCTATTTCGTACAATCGATGGATTAAGGGCGGATGTGATCTTCATAGAGTAATTTATAACATAAATAATTTGAATTATAAACATAAAAATCTGTGAATCGATCACAAAGTATCTTGAGCAGAAACACCTCGTTTATTTGAGGGAGCCAAGCATTCCTCTTTGCACTAACCAAAAGCTCTTACATATTAAATACAAGTTGCTAATTACATTTTAGCTAACAAACAAGAGGAACAGTTCATCTCTTTTTAAAGCAACAAGTAAGCAGTCGTCTTCCAACCCTCCACCGATCTCTATACATTAGAAATCAATGGGTTGGGATGAGCTGTCCTGCTAGTGCATTAGATGCTTTTATCTGCATGCTTACGCGCGTTGTCTAGCTGCTTGCTGCTTGCTTCCCTGCCCTCGACTGGGAGAAACTTGACTCACCCTCCCACTCCCTCCCTCCCTCCTATATCTGTAATTTACCATTTCACAACATATTCTGGTCTGACTCTGAAATTCTGCAGAATGGTAAAAGAAATATATGAGTGGAAAACTCTGATATTTATCTTGTTTATGAATGTGGGCACCTCTATTATTGTTAAAAGGGCCGCTGGAGGACTGTTGAGAAGACCAAGTTGCCCCTAGAACAGTGTTGGGTGGGTGGGTGACTTTGGGTTTTCAAAGCACAGAAAAAAAAAGATGGAAAATTAAGGAGAATGCTTATAGAGAGAGAGAGAGAGAGAGACTGTTGGATGTAAAACCAAAAAAATATGAATAAAATTACTGGGGACGAGGAGGGGTTCAGGCAGGCATAAATGTGAGTGTAAAAAAGAGAGGTGGATTCTTATCATTCCTTTTACCAGACCCCACGAGTGGAGGGCGGAGGGCGGAGGGCGGAGAGGTTTTCTCTTTCTCTCTCTCGTCTTACTTCCTTCCTCATAGCTAGTAGCTCCACCACAAAGGTCCCTCACCTGCTTGACCTAATTTCTCTCTCTAAAGAGTAAGTGCCACTTCTGGTGCTGCGCAGGGGAAGGACAAGAGTTAATCATAGACCAAGAAAGTAAGAACCCCACAAACTCAATCTTAGTCGGTCTTAGCTTGGTCCAACTCCAATCCAATCCCTGCAATATTTTTAAAGAGATTCTCACTCCACCCCAGCCCCTCTCTCTCTCTCTCTCTCTAAAGTCCAAAGTCCAACCCAGCTCAAGAGAGAGAAAGACAAGAGAGAAGAAAAAGAAAAGTACTATTGTGGATGACTGATGAGTGAGAGAGAGATGGAGTTGATCAAGATCAATCAGCCAACAGCAACTTAAAACCCAAGAAACAAAAAAACACATAAAAATAAAGATATCATCTCTCTCTCTCTTCCTCCAAGTCCAAACCCTTCCCTACCAA |
| Pbr011454.1 | CTAGAGCACTTATTTTTGTATCAGTGAAATTTTGAATTTGATTTCCGTCTTCGTGTCCTTTTTCTTAAATTTTTTTTTTAAAAAATCAATATTAAATTAAATCTATTTGATTTGTTTTTTGGTGTTTGATCATGATCTTTTCCTATTAAGAACGGAAAAATAAAAAAATTAAGGCGCTTATAGTTAAACCTTTATTCATGCGTTCGTAGTTCTATAAGTTTGATTTCAGCCTCCCTTAGGCCGTCTCCAACCGACCCGATCAAAGGATCATAGGGCTAAAAAGAACTCGAAATGACACAAAAACCGTCTCAAACCAAGGGCTCGTGGGCCCTACTAGGCCAAAACCACCAAATCAGGCCAGCGAGCTGGCCATTTCCAGCTAGCTCGATAAGCCCAGCCCCCTTTTTTTTTTTTTTGACTTAATTTTTTTTTTTACACCATTCCATACTATTTTATCTCATTTTATTCAATTCTTCACATCCTATTCTCTTCTAATAATATTTCCTTTAATTTTTTCAATAATTTTCAAATGGCCACCTCTTTCAATAATCTTCCAATATGTCCGAAAATCTTATTTTAATACAGTAGTTTAATTTAATTAAATTCAATTAAAATAATAAATTATGTTTGGCCTATGGCCCTTTAGCCCATTCGGTTGGGAATGGTATAAAAATATGGTATGTCACTATTCATTAAAATAAAATTTCTTACATGACTATAGGGCTAAATATAGCCTCTTGACACTCCTTCGATTAGAGATGGCCTTGGACCCAGCTTCTCCGCCCTCCCACTTCTCATACACTCTCATCCCCTCCAATTTTGTGCGGCCACGGTTAAGCTACGTCAACATTTTATATTGATTTTTTTTATAGAGATAATAAGACAAAAAATAATAGTAATATAAAATGTTGACGTGGCTTAACCGTGACCGCACAAATAGGAGGGGATGAGAGTGTATGGGAAGTGGGAGGGCAGAGAAGCCAGGACCGATGGCCTTACTATTATTTATATAATTTTAGATATATAATAAAAAAATTAAAATATAAAAGCTAGAAAAAGCTCAGCACAACAACAAAGCAATTTCTTTTCCTACAAAAAATTGCTTTTTAAAGCTCACCCATATAATTCCCACTCATTATCTTTGTTGCTTAAAGCTTACCTGCCCAGTGCCCAGCTCTCTCTCTCGCTCACTTCCTCCCTCCCTTATCTTCTCTCTCTCTCTCTCTTTCCAAAGGACCATTTTAGTCCATCCTTCTTTCTCTCTCTAGATATCTCTAGCGCTACTATACAATTCTTAACCCAAAACACATCACCTAAACCAAAATCTCAATTTTTTTCGTACACAAATTCCAGCCAACAAACAACCAGAGATTTTTATTTATTTATTTTAATAAAAACAGGCTTGTTCTGTAAAAATATAGTGCGAGTGAGTGACTGAGTGAGACTGAAGAGAGCTGAGAGTTGAGTTGATCAGCTGCTGAGTGAGAGACCCACCAAAACCCAATTATACTAAACTAAGCCCTCTCTCTCTCTTCCACTCTCTCTCTCTCTCTCTCTACTCTTCTCTCATAAAACCCTCCACAAGCTGATCATAATTCTCTTTCCCATGCGATC |
| Pbr006457.1 | CCTAGTTTTCTTTTGAAAAAAAAAATCCTTGTTTTCTTCTTTAAAAAAAAAGAGAGATCAGTTGGTGGCTTCATCACGAAATCAGTGGCATAGCTAGCATAGGGTCATTAGTTGTCCTTGACCCCAACAATTTTGAAAACTCCATGTATATTTGTTTATGTATTATATTTGACCCTTATAAACACCTAAGGCAAACTAAATTACAATCTCCCCCTCCTACTTCATGTGTGCGACTCTATTGTGCAATCATGTGCTCGATGAAATTCTTTAGTTTTTCTCTCTTCTTGTGTGCGATCATGTGCTCGATTAAATACCTTAGTTGAAAAGCCTAGGCAATCTTCAATATTACTCCACAGTATCATGCATCAACCCTAGACAAGCATTTACTAATAATAGTAGTTGTGATATACGTTAGCATAAACTTTCGACATATGTTGACAGGAATTGGCAGTGCTTCGTAAGTGCTCGATGAATGGCTAACGAAGTTTTTTTGGTTGATATAATGACCCCATTTTTCAAAATCATGGCTACGGCACTGACAACAACCCACTTTTTTCGAAGGTCAGGAAGACTTATAAAGACATTCAGCCTTCTCATAGTCATCATCATGTGATTTAATATCATAAAGTATCGTACTTAGAACACACCGTGTGAAATAAAAGTTTGAAATTCATCTTCAGTTAACTCCTAAATCATAGTTAATTAACTCCTATTTTGTCACAATTAATGATTATTTAACCTTATGCATGCGTGATGAGGAAATGATGAGATATCGACTAGCTACCATAGCTACACCCACATTCGTTTGAGTTGATGGTGAGAGTGCCACGTGGCATTGTATGTACCACTGCTTTATCTTTCCTTGGGCATGGTCGGTGGAATTCCGGCGCCTTACTTTTTCCTCTCAAACGGTTCTCTCTACTTCTTCTTCAACAAGTTTTATTCTCATATTCTCCACATACTTGCAGAATAATTTAATCTTCAAAAGTACAGACGGCTGTTTGGTTGATTTTAGTAGTTCTCTACTAAACTCACAAAGCCAATTTCAAGTTGAGAAAATCACAACCACATACCTTTCTTTGCCATTCTGTGTAATACTGTGTATTCAACTCACCAAGCACCGAAAACTGTCTGCAAAACAAGAAGCAGAAAATGTTTACTTTCATGTAAGTTGGGTCAATTTCAAGTAATATGTTGGTTGGGTAACCCTAGAATACTTCATAATTATAAAATAATTAGCATCACATACCTAGCTTTATATTTGTTGCTTTGCTTTAATTCCATTAATATATATACGCGAGACAAGTGAACTTGGAATTCCTTAACTAAACATTTCTACAGCTTGTGAATCGCTTCTTCTTTTGAGCTCCTTTTTAATTTCGTTCATGAAACAATTCATGAAAGTTGTATTAGTTTCGTTTTTAATTTTGTGGATCATCACATATATGTCATAGATATTAGCTAGCTTAATTAGTAGGAAACACTTTTTGATAATTTTTATTTTTTTTATAATTCATTTCTTTCATGATTGATGGTGTACCAGATTATGATAAAAAAAGAAAGCACATGAAGAAGAGAAGCATATCATGATTAATTCGTGAAGTTATCATTGCAAAGAGAATAGAAATCTGCGTGTTGAGCTGAAATGATGAACCAATGACGAGAGAGTCCAGAAG |
| Pbr007125.1 | ATTTTTTCTTATATTTTGCTTAGCTTTTGATATTGTTGACTTCTCATGTGAGTTGTGTATAATTAATACAAGGACTATTCTAACTAGTGAAAATTAGTGATGCAAACAATGTGACTTGTATGATGATATGCATCAAAACTGAAACTGTAAACAAATATCATATATGAGGAGTAATTAAACTTTTTTAAGAATTTGTAAGGTTGCTTAATTTTTCGATGTGGGACAACACATCACATTCTCACATGCCCGTTACATGTGGCAATGGTCAAATAAAACAGGTAGGCAATACATATTAGGTTGCATGAATCATGTGTAACCGTTGTGCTTCACGCGTGAGCCAATTGTCTCTAATAACATTTAAGAATTTTGAGGTTTCACTAAAAAATTAATTAGCAATATGGAGAGTAACCAGCTCGTTATAAGGACATGCAAGATCGCTGAACTTTTTGACGTGGGACATCACTCCACATGCAATATTCAATGAAGAAGAATAAGTGTTAGAACTCATTTGGATGTACTTTTAAAATGACTGAAAACACTTTTGGTGAAAATGTTTTTAGAACCAATCCTTAATAAACATGCAAGTAAATTCTGGAAAAGCACTAAAAGTGCTTTTGGAATTCAAAAATATTTTCTTTAAAAGTACTTTCAATTATTTTAAAAGCACATCCAAACGAGCCATTGATTATGGAATATTAGTTGAATCTTCCCAAAACAAACCACAAAATCCACTCTCCATCTCCATGTATGCGTAACACTATCTAGCTACTGTGGGGTGTGTCTATGCAAGGTCTTTATATTTTGAGATTGAGAAGCTAGGTTATTGAATTGATCTATACCGGGCGACCTGCAAACCACGTGGACTGGTGGTGCCAAATACTAGGTGTTTTCCTATCCACTCGCTTCCTTTCACACACAGTGATATTCTAAATGGTAGGGCTAACTTTTTGATGAAAAGATTAAGAAAAAGATAGCTACTCAGCAATAAATTGCCCCCTACCCTCCCTTGCTTCTTTACCATACCACTACCTTACTAGCTTCTCTCCCTCTCTTTCTCTCTCTCTCCCCCTGTGATTCAAGAGTTCAACCCCCATGGACATATGGTGTTCTTGCTGTGCTGTCTTTCTGAACCTCTCTATTTCCTACGATGACGACCGATGGTAAATGTACGATCTCTCTCTGTCTTTTTCTAGCTAGCTAGCCTTCTCTCTCTCTCTCTCGCGCACACACAAAAGAAACTAAATAAATACAGTACATGTAGTAAAGGTGTACATATATACATATACGTACAATATATATACATATATGTACAAGCATATAGAGGTATATATGAGTAGCACTGCAGGCAGCATCTGTCTTTCTTAAGCTAAGCTCAGCTGGCCGTAAATACGGTAACACTCGACTGTCTTTCTCAAAGCTATCCCTTCCCTTAATTTTGATTTACTACTCTTGCAGAAAAGGTGAAAAACAAGCGCACTGAAATCACATTCAGCGGCATTCGCTTCCTCATCAAGCATTTATAAAAGCGCAAGTCTCGATCATCAGTAGACCGATATCCCAAGCGTACCAATTGACCACTAGCTGCTAGCTAGCTAGGGTTAGTGGTGTGCAATTTCATTGAAAATTCAAAGCAACCAAACAAGCAAAGAGCCCAAGAGGGAGATATATAAC |
