# Supplementary material for: Comparative Genomic Analysis of TCP Genes in Six Rosaceae Species and Expression Pattern Analysis in Pyrus bretschneideri
Source: Front Genet. 2021 May 17;12:669959. doi: 10.3389/fgene.2021.669959 (PMC8165447; doi:10.3389/fgene.2021.669959)
Supplement: Supplementary file 16 [file Data_Sheet_1.PDF]

Editing Certificate

This document certifies that the manuscript

Genome-Wide Identification of TCP Family Members in Six Rosacee Species And...

prepared by the authors

Yu Zhao,Xueqiang Su,XinYa Wang,Mengna Wang,Xiaofeng Feng,Guo Hui ...

was edited for proper English language, grammar, punctuation, spelling, and overall style by one or more of the highly qualified native English speaking editors at AJE.

This certificate was issued on **March 22, 2021** and may be verified on the AJE website using the verification code **48CC-A9F0-7F3C-FFAC-C544**.

Neither the research content nor the authors' intentions were altered in any way during the editing process. Documents receiving this certification should be English-ready for publication; however, the author has the ability to accept or reject our suggestions and changes. To verify the final AJE edited version, please visit our verification page at aje.com/certificate. If you have any questions or concerns about this edited document, please contact AJE at support@aje.com.

AJE provides a range of editing, translation, and manuscript services for researchers and publishers around the world. For more information about our company, services, and partner discounts, please visit aje.com.
